# Machine-Learning Classifiers in Discrimination of Lesions Located in the Anterior Skull Base

**DOI:** 10.3389/fonc.2020.00752

**Published:** 2020-05-28

**Authors:** Yang Zhang, Lan Shang, Chaoyue Chen, Xuelei Ma, Xuejin Ou, Jian Wang, Fan Xia, Jianguo Xu

**Affiliations:** ^1^Department of Neurosurgery, West China Hospital, Sichuan University, Chengdu, China; ^2^Department of Radiology, Sichuan Provincial People's Hospital, Sichuan Academy of Medical Sciences, Chengdu, China; ^3^Department of Biotherapy, Cancer Center, West China Hospital, Sichuan University, Chengdu, China; ^4^State Key Laboratory of Biotherapy and Cancer Center, West China Hospital, Sichuan University and Collaborative Innovation Center for Biotherapy, Chengdu, China; ^5^West China School of Medicine, West China Hospital, Sichuan University, Chengdu, China; ^6^School of Computer Science, Nanjing University of Science and Technology, Nanjing, China

**Keywords:** pituitary adenoma, meningioma, craniopharyngioma, Rathke cleft cyst, anterior skull base, radiomics, machine learning

## Abstract

**Purpose:** The aim of this study was to investigate the diagnostic value of machine-learning models with radiomic features and clinical features in preoperative differentiation of common lesions located in the anterior skull base.

**Methods:** A total of 235 patients diagnosed with pituitary adenoma, meningioma, craniopharyngioma, or Rathke cleft cyst were enrolled in the current study. The discrimination was divided into three groups: pituitary adenoma vs. craniopharyngioma, meningioma vs. craniopharyngioma, and pituitary adenoma vs. Rathke cleft cyst. In each group, five selection methods were adopted to select suitable features for the classifier, and nine machine-learning classifiers were employed to build discriminative models. The diagnostic performance of each combination was evaluated with area under the receiver operating characteristic curve (AUC), accuracy, sensitivity, and specificity calculated for both the training group and the testing group.

**Results:** In each group, several classifiers combined with suitable selection methods represented feasible diagnostic performance with AUC of more than 0.80. Moreover, the combination of least absolute shrinkage and selection operator as the feature-selection method and linear discriminant analysis as the classification algorithm represented the best comprehensive discriminative ability.

**Conclusion:** Radiomics-based machine learning could potentially serve as a novel method to assist in discriminating common lesions in the anterior skull base prior to operation.

## Introduction

A variety of lesions are present in the anterior skull base. The most common types of tumors in this area are pituitary adenoma, craniopharyngioma, and meningioma (1, 2). Rathke cleft cyst is also taken as the common differential diagnosis for the sellar mass as a congenital lesion (3). The importance of early diagnosis for lesions in this region has been highlighted because even these benign lesions may be progressive and unrelenting if situated in an area where growth cannot be controlled, and some of them could show aggressive behavior (4). Magnetic resonance (MR) scan is highly recommended for preoperative evaluation of the anterior skull base lesion owing to the advantage of excellent soft tissue resolution. Descriptions of the four types of lesions in MR imaging (MRI) are characteristic (5). However, the diagnostic accuracy of MR images depends on experiences of radiologists, and in some cases, these lesions with similar MRI patterns may mimic each other and complicate the radiological diagnosis (6, 7). Therefore, new methods that could assist in preoperative differentiation may be of clinical value.

Radiomics could extract high-dimensional features from medical images and provide information associated with the pathophysiology of lesions that is difficult to be assessed by visual inspection (8–10). Moreover, mineable radiomic features of lesions could be analyzed with the novel machine-learning technology that has shown promising prospects in the biomedical domain (11). Radiomics-based machine learning has been applied in differential diagnosis of various brain tumors in previous studies, representing the potential to be utilized in clinical practice to facilitate diagnosis, and offer guidance for decision making (12–16). In the present study, we evaluated the ability of machine-learning technology combined with MRI radiomic features and clinical parameters in differentiating the four common types of lesions in the anterior skull base. Considering the epidemiology and position of lesions, the differential analysis was divided into three groups: pituitary adenoma vs. craniopharyngioma (the most common tumors in the sellar/suprasellar region), meningioma vs. craniopharyngiomas (the most common tumors in the parasellar region), and pituitary adenoma vs. Rathke cleft cyst (the most common lesions in the intrasellar region).

## Method

### Patient Selection

**Figure 1:**
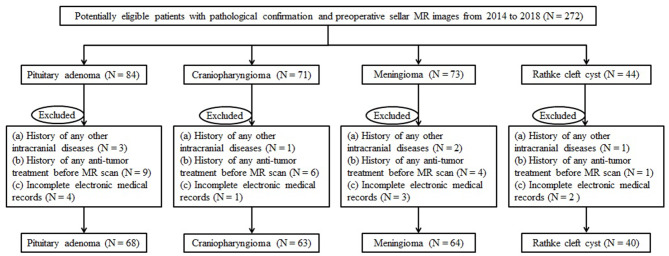
Workflow chart of the patient enrollment process.

Institution database was reviewed to search for patients treated at our neurosurgery department from November 2014 to June 2018. We initially selected the potentially qualified patients according to the following criteria: (a) with the pathological confirmation of pituitary adenoma, craniopharyngioma, meningioma, or Rathke cleft cyst; (b) the lesion was located at the anterior skull base; and (c) with preoperative sellar MR images. Exclusion criteria were as follows: (a) history of any other intracranial diseases, such as stroke and intracranial infection; (b) history of any anti-tumor treatment prior to MR scans, such as brain surgery, chemotherapy, or radiotherapy; and (c) incomplete electronic medical records. The flowchart of patient selection is shown in Figure 1. Clinical parameters were recorded, including age, gender, lesion size, and the time between MR scan and surgery. The lesion size was measured by the maximum diameter of the lesion that was collected from radiological reports. This retrospective study was approved by the institutional review board. The written informed consent was obtained from all participants (written informed consent for patients <16 years old was obtained from their parents or guardians).

### Image Acquisition

**Figure 2:**
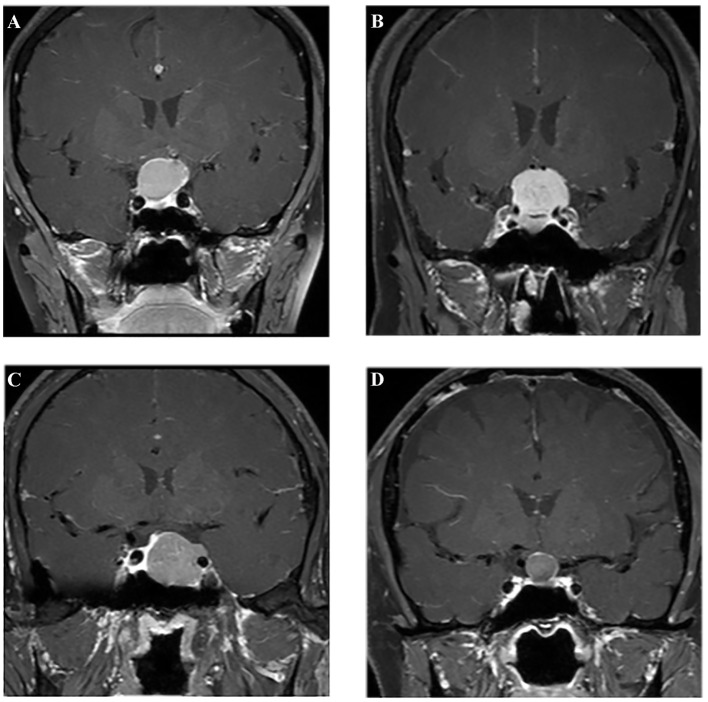
Examples of different lesions on contrast-enhanced T1-weighted image. **(A)** Craniopharyngioma; **(B)** meningioma; **(C)** pituitary adenoma; **(D)** Rathke cleft cyst.

All patients underwent MR scans *via* a 3.0-T GE scanner with an eight-channel phase array head coil. The parameters of the contrast-enhanced T1-weighted imaging were as follows: TR/TE = 552/10 ms, slice thickness = 5 mm, flip angle = 90°, field of view = 150 × 150 mm^2^, data matrix = 256 × 256, and voxel size = 1.0 × 1.0 × 1.0 mm^3^. The scanning was conducted within 200 s after injection of gadopentetate dimeglumine (0.1 mmol/kg) as the contrast agent. The preoperative MR images were collected from picture archiving and communication system (PACS) of our institutional radiology department (Figure 2).

### Feature Extraction

Texture features were extracted from MR images as radiomic parameters by two neurosurgeons together with the assistance of senior radiologists using LIFEx software (http://www.lifexsoft.org) (17). Following protocols of the software, the region of interest (ROI) was manually drawn within the border of the lesion in each slice. Considering the clear depiction of the boundary of lesions, ROI delineation was performed on the contrast-enhanced T1-weighted imaging, in which lesions were carefully separated from adjacent brain tissues through different enhancement patterns and surrounding anatomic structures. Any disagreements regarding the border of lesions were recorded and solved by senior radiologists. After the whole lesion was contoured slice by slice, three-dimensional radiomic parameters could be automatically calculated by the software with established formulas (Supplementary Material 1). A total of 40 features were obtained from two orders, including the first-order features from shape-based matrix and histogram-based matrix, and the second-order/higher-order features from gray-level co-occurrence matrix (GLCM), gray-level zone length matrix (GLZLM), neighborhood gray-level dependence matrix (NGLDM), and gray-level run length matrix (GLRLM) (Supplementary Material 2). Examples of ROI delineation are shown in Supplementary Material 3. Combined with two clinical parameters (age and gender), a dataset was built for further analysis.

### Machine-Learning Modeling

Given that there was a relatively large number of statistics and some parameters may not be associated with the differential process, optimal features should be selected first for the predictive model. The feature-selection method was important but complicated considering the sample size and efficiency in discrimination. Least absolute shrinkage and selection operator (LASSO) regression model was reported to be appropriate for high-dimensional data regression analysis (18, 19). Other selection methods were also evaluated by previous researchers and reported to represent good diagnostic performances (20, 21). To settle the dilemma, five different feature-selection methods were adopted, namely, distance correlation, random forest (RF), LASSO, eXtreme gradient boosting (Xgboost), and gradient boosting decision tree (GBDT). A similar predicament also needs to be solved in regard to the selection of machine-learning classifiers. We employed nine classification algorithms in this study, including linear discriminant analysis (LDA), support vector machine (SVM), RF, Adaboost, *k*-nearest neighbor (KNN), GaussianNB, logistic regression (LR), GBDT, and decision tree (DT). Patients were randomly divided into the training group and the testing group at the ratio of 4:1 on the basis of experiences from previous studies (22–24). The model was first created by the training group and then applied to the independent testing group, and this procedure was repeated over 100 times to conclude the realistic distribution of classification accuracies. Area under receiver operating characteristic (ROC) curve (AUC), accuracy, sensitivity, and specificity were calculated based on the confusion matrix to assess the discriminative ability of different models. Regular statistical analyses of this study were performed using SPSS (Version 22.0, IBM Corp. Armonk, NY, USA), and machine-learning algorithms were programmed with Python Programming Language and scikit-learn package.

## Results

### Patient Characteristic

A total number of 235 patients who underwent surgical resection of lesions in our neurosurgery department were enrolled in the current study. Among all participants involved, 68 patients were diagnosed with pituitary adenoma, 63 patients were diagnosed with craniopharyngioma, 64 patients were diagnosed with meningioma, and 40 patients were diagnosed with Rathke cleft cyst. The average age of patients was 49.16, 31.62, 49.19, and 44.23 years, respectively. The mean value of the maximum diameter of lesions was 23.21, 28.86, 20.41, and 19.87 mm, respectively. The characteristics of patients and lesions are summarized in Table 1.

**Table 1:** Characteristics of patients and lesions.

	**Craniopharyngioma**	**Meningioma**	**Pituitary adenoma**	**Rathke cleft cyst**	***P*-value**
Number	63	64	68	40	
Gender, *n* (%)					0.006
Male	37 (58.7)	18 (28.1)	32 (47.1)	18 (45.0)	
Female	26 (41.3)	46 (71.9)	36 (52.9)	22 (55.0)	
Age, *n* (%)					<0.001
≤ 18 years	21 (33.3)	2 (3.1)	1 (1.5)	0 (0.0)	
19~30 years	11 (17.5)	0 (0.0)	7 (10.3)	11 (27.5)	
31~60 years	27 (42.9)	51 (79.7)	43 (63.2)	26 (65.0)	
>60 years	4 (6.3)	11 (17.2)	17 (25.0)	3 (7.5)	
Mean age (range) (year)	31.62 (2~73)	49.19 (9~72)	49.16 (18~73)	44.23 (21~68)	
Maximum diameter (mm)	28.86 (12.5~52.4)	20.41 (8~40)	23.21 (7~50.5)	19.87 (8~38.3)	<0.001
Average time between MR scan and surgery (day)	6.2	7.5	5.3	6.4	0.321

*MR, magnetic resonance*.

### Machine-Learning Model Assessment

In each group, 45 diagnostic models were established through the combinations of five selection methods and nine classifiers. The combination of LASSO as the selection method and LDA as the classifier (LASSO + LDA) seemed to be the optimal model in differentiating common lesions in the anterior skull base with AUC of more than 0.80 in all three groups. It is worth noting that some combinations represented better performance than LASSO + LDA in a single group, but LASSO + LDA showed the best comprehensive discriminative ability.

Group 1 was the differentiation between pituitary adenoma and craniopharyngioma considering these are the most common tumors located in the sellar/suprasellar region. For LASSO + LDA, ROC analysis demonstrated that AUC, accuracy, sensitivity, and specificity in the training group were 0.845, 0.851, 0.897, and 0.820, respectively. In the testing group, this predictive model was proven to be feasible in discrimination with AUC of 0.804, accuracy of 0.800, sensitivity of 0.888, and specificity of 0.734 (Table 2). Besides, other models like RF + RF (AUC = 0.811 in the testing group) and GBDT + RF (AUC = 0.837 in the testing group) also represented feasible ability in distinguishing pituitary adenoma from craniopharyngioma (Table 3).

**Table 2:** Results of the discriminative model of LASSO + LDA in distinguishing lesions in the training group and the testing group.

	**Training group**				**Testing group**			
	**AUC**	**Accuracy**	**Sensitivity**	**Specificity**	**AUC**	**Accuracy**	**Sensitivity**	**Specificity**
Pituitary adenoma vs. craniopharyngioma	0.845	0.851	0.897	0.820	0.804	0.800	0.888	0.734
Meningioma vs. craniopharyngioma	0.882	0.881	0.944	0.832	0.807	0.819	0.863	0.794
Pituitary adenoma vs. Rathke cleft cyst	0.873	0.887	0.861	0.901	0.816	0.836	0.829	0.840

*AUC, area under curve; LASSO, least absolute shrinkage and selection operator; LDA, linear discriminant analysis*.

**Table 3:** Results of discriminative models in distinguishing pituitary adenoma from craniopharyngioma in the testing group.

	**Distance correlation**		**RF**		**LASSO**		**Xgboost**		**GBDT**	
	**Accuracy**	**AUC**	**Accuracy**	**AUC**	**Accuracy**	**AUC**	**Accuracy**	**AUC**	**Accuracy**	**AUC**
LDA	0.719	0.727	0.778	0.782	0.800	0.804	0.730	0.734	0.793	0.799
SVM	0.696	0.712	/	/	0.700	0.717	0.700	0.696	/	/
RF	0.727	0.747	0.804	0.811	0.770	0.780	0.781	0.786	0.841	0.837
Adaboost	0.796	0.799	0.833	0.837	0.785	0.784	0.770	0.774	0.833	0.831
KNN	0.800	0.800	0.689	0.690	0.756	0.765	0.689	0.694	0.722	0.727
GaussianNB	0.744	0.750	0.726	0.730	0.670	0.681	0.715	0.724	0.737	0.741
LR	0.752	0.758	0.822	0.819	0.774	0.783	0.693	0.705	0.767	0.771
GBDT	0.796	0.796	0.859	0.857	0.874	0.866	0.811	0.809	0.844	0.840
DT	0.752	0.754	0.800	0.798	0.767	0.766	0.763	0.757	0.785	0.783

*RF, random forest; LASSO, least absolute shrinkage and selection operator; Xgboost, eXtreme gradient boosting; GBDT, gradient boosting decision tree; LDA, linear discriminant analysis; SVM, support vector machine; KNN, k-nearest neighbor; LR, logistic regression; DT, decision tree; AUC, area under curve*.

*/, over-fitting*.

Group 2 was the differentiation between meningioma and craniopharyngioma, given that they are the most common tumors located in the parasellar region. ROC analysis illustrated the differential ability of LASSO + LDA with AUC of 0.882, accuracy of 0.881, sensitivity of 0.944, and specificity of 0.832 in the training group. In the testing group, AUC of LASSO + LDA was 0.807, accuracy was 0.819, sensitivity was 0.863, and specificity was 0.794 (Table 2). Besides, distance correlation + LDA (AUC = 0.843 in the testing group), RF + LDA (AUC = 0.842 in the testing group), GBDT + LDA (AUC = 0.809 in the testing group), and distance correlation + KNN (AUC = 0.846 in the testing group) also represented reliable diagnostic performance in discrimination between meningioma and craniopharyngioma (Table 4).

**Table 4:** Results of discriminative models in distinguishing meningioma from craniopharyngioma in the testing group.

	**Distance correlation**		**RF**		**LASSO**		**Xgboost**		**GBDT**	
	**Accuracy**	**AUC**	**Accuracy**	**AUC**	**Accuracy**	**AUC**	**Accuracy**	**AUC**	**Accuracy**	**AUC**
LDA	0.846	0.843	0.850	0.842	0.819	0.807	0.800	0.792	0.815	0.809
SVM	0.807	0.804	/	/	0.712	0.732	/	/	/	/
RF	0.769	0.777	0.773	0.780	0.735	0.744	0.796	0.798	0.812	0.822
Adaboost	0.753	0.766	0.746	0.758	0.777	0.784	0.784	0.790	0.773	0.781
KNN	0.838	0.846	0.708	0.713	0.742	0.746	0.669	0.663	0.650	0.656
GaussianNB	0.762	0.753	0.777	0.778	0.700	0.681	0.715	0.691	0.804	0.787
LR	0.796	0.800	0.777	0.783	0.765	0.763	0.735	0.725	0.785	0.780
GBDT	0.769	0.773	0.769	0.774	0.773	0.782	0.765	0.769	0.812	0.816
DT	0.742	0.744	0.723	0.722	0.712	0.710	0.719	0.726	0.765	0.767

*RF, random forest; LASSO, least absolute shrinkage and selection operator; Xgboost, eXtreme gradient boosting; GBDT, gradient boosting decision tree; LDA, linear discriminant analysis; SVM, support vector machine; KNN, k-nearest neighbor; LR, logistic regression; DT, decision tree; AUC, area under curve*.

*/, over-fitting*.

Group 3 was the differentiation between pituitary adenoma and Rathke cleft cyst, which are the most common lesions in the intrasellar region. In the training group, ROC analysis demonstrated that AUC of LASSO + LDA was 0.873 with accuracy of 0.887, sensitivity of 0.861, and specificity of 0.901. In the testing group, this model also represented feasible discriminative ability with AUC of 0.816, accuracy of 0.836, sensitivity of 0.829, and specificity of 0.840 (Table 2). In addition, distance correlation + RF also represented good performance in differentiating pituitary adenoma from Rathke cleft cyst with AUC of 0.825 in the testing group (Table 5).

**Table 5:** Results of discriminative models in distinguishing pituitary adenoma from Rathke cleft cyst in the testing group.

	**Distance correlation**		**RF**		**LASSO**		**Xgboost**		**GBDT**	
	**Accuracy**	**AUC**	**Accuracy**	**AUC**	**Accuracy**	**AUC**	**Accuracy**	**AUC**	**Accuracy**	**AUC**
LDA	0.841	0.803	0.827	0.804	0.836	0.816	0.754	0.682	0.786	0.767
SVM	0.754	0.678	/	/	0.627	0.500	0.645	0.544	/	/
RF	0.863	0.825	0.768	0.714	0.777	0.710	0.855	0.813	0.823	0.775
Adaboost	0.813	0.794	0.804	0.774	0.818	0.778	0.818	0.778	0.836	0.809
KNN	0.786	0.735	0.745	0.696	0.732	0.666	0.759	0.706	0.768	0.723
GaussianNB	0.841	0.806	0.814	0.786	0.677	0.655	0.800	0.745	0.827	0.792
LR	0.800	0.736	0.823	0.781	0.755	0.683	0.818	0.764	0.805	0.769
GBDT	0.845	0.808	0.832	0.798	0.786	0.743	0.818	0.776	0.850	0.821
DT	0.832	0.798	0.791	0.761	0.736	0.700	0.786	0.757	0.809	0.780

*RF, random forest; LASSO, least absolute shrinkage and selection operator; Xgboost, eXtreme gradient boosting; GBDT, gradient boosting decision tree; LDA, linear discriminant analysis; SVM, support vector machine; KNN, k-nearest neighbor; LR, logistic regression; DT, decision tree; AUC, area under curve*.

*/, over-fitting*.

**Figure 3:**
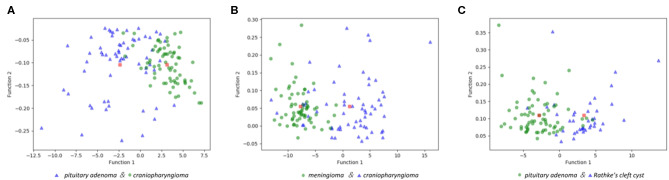
Relationships between the discriminant functions for different lesions in the three groups and for the group centroids. **(A)** Pituitary adenoma vs. craniopharyngioma; **(B)** meningioma vs. craniopharyngioma; **(C)** pituitary adenoma vs. Rathke cleft cyst.

**Figure 4:**
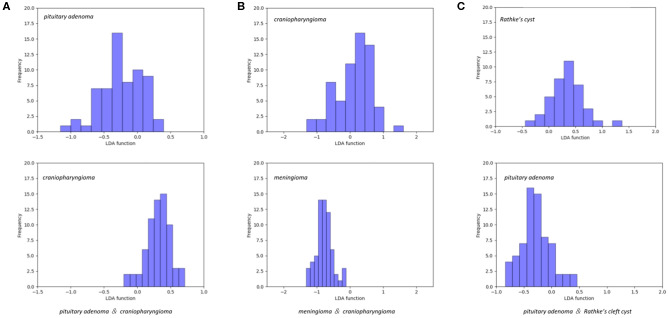
Examples of distributions of the linear discriminant analysis (LDA) function determined for the lesions for one cycle. **(A)** Pituitary adenoma vs. craniopharyngioma; **(B)** meningioma vs. craniopharyngioma; **(C)** pituitary adenoma vs. Rathke cleft cyst.

The features selected into LASSO + LDA model are listed in Table 6. The association between discriminant functions for LASSO + LDA model is represented in Figure 3, in which minimal overlap between two clusters was observed in each group. Figure 4 represents examples of distributions of the direct LDA function for lesions for one of the 100 independent cycles. In group 1, a shift of the LDA function values for craniopharyngioma toward positive values was shown while predominantly negative values for pituitary adenoma. Similar trends could be observed in group 2 and group 3, suggesting that the LASSO + LDA model had feasible discriminative ability in the three groups.

**Table 6:** Parameters selected in the discriminative model of LASSO + LDA.

**Group 1**	**Group 2**	**Group 3**
Age minValue meanvalue maxValue SHAPE_Volume GLCM_Contrast GLRLM_HGRE GLRLM_SRHGE GLRLM_LRHGE GLRLM_GLNU GLRLM_RLNU GLZLM_LZE GLZLM_SZHGE GLZLM_LZLGE GLZLM_LZHGE GLZLM_GLNU GLZLM_ZLNU	Age minValue meanValue stdValue maxValue SHAPE_Volume GLCM_Contrast GLRLM_HGRE GLRLM_LRHGE GLRLM_GLNU GLRLM_RLNU GLZLM_LZE GLZLM_HGZE GLZLM_SZHGE GLZLM_LZHGE GLZLM_GLNU GLZLM_ZLNU	minValue meanValue maxValue SHAPE_Volume GLRLM_SRHGE GLRLM_LRHGE GLRLM_GLNU GLRLM_RLNU GLZLM_LZE GLZLM_HGZE GLZLM_SZHGE GLZLM_LZHGE GLZLM_ZLNU

*GLCM, gray-level co-occurrence matrix; GLZLM, gray-level zone length matrix; NGLDM, neighborhood gray-level dependence matrix; GLRLM, gray-level run length matrix; LASSO, least absolute shrinkage and selection operator; LDA, linear discriminant analysis*.

## Discussion

In the present study, a series of clinical parameters and radiomic features were utilized in differentiating four types of lesions in the anterior skull base. The predictive models were built using five feature-selection methods (distance correlation, RF, LASSO, Xgboost, and GBDT) and nine machine-learning classification algorithms (LDA, SVM, RF, Adaboost, KNN, GaussianNB, LR, GBDT, and DT). The combination of LASSO as the feature-selection method and LDA as the classification algorithm represented the optimal comprehensive performance with AUC of over 0.80 in all of the training groups and the testing groups. Moreover, several models also showed reliable discriminative ability between two types of lesions in a single group. Considering the features we selected could be extracted from routine MR images, the predictive model has the potential to be utilized as a novel, convenient tool in clinical practice.

The most important result of our research was to identify suitable discriminative models for lesions located in the anterior skull base. In previous studies, researchers investigated various combinations and tried to identify the optimal diagnostic or prognostic model. For instance, one study on CT-based survival prediction of non-small cell lung cancer involved models with four selection and classification methods (25). Another study made evaluations on models with 14 selection and 12 classification methods in predicting the overall survival of lung cancer patients (26). Similar studies were performed in bone tumor and head and neck cancer, implicating that the machine-learning model could potentially be a reliable method in differential diagnosis and prognosis prediction (27–30). However, it brought our attention and further investigation that various classifiers were used but that unanimous results on which one could be taken as the universal method were not reached. Considering that the purpose of clinical application of machine learning is to lessen the workload for doctors, simple discriminative models between two types of lesions are relatively meaningless because of complicated and elusive situations in clinical practice. Based on this idea, not only different combinations were tested, but also analyses on four types of lesions were performed simultaneously in three groups in the present study.

LASSO is a brilliant feature-selection method that tries to retain useful features in both ridge regression and subset selection (31). With the characteristics of avoiding over-fitting, it is suitable for large sets of radiomic features when a relatively small number of samples are involved (28). LDA is a machine-learning classification algorithm that could find a linear model with the best discriminative ability for two classes. The mechanism of LDA is to identify the boundaries around clusters of two classes and to project the statistics into a lower-dimensional space with good discriminative power based on the distance to a centroid of each cluster (32). LDA was reported to be able to reduce the dimensionality and to preserve the class discrimination information as much as possible. The combination of LASSO and LDA showed optimal comprehensive results in all three groups with AUC of more than 0.80 in each group. However, the results of our study were not as good as those of others. One study on the prediction of ATRX mutation in low-grade gliomas represented brilliant results with AUC of 0.925 in the validation group (19). Another study on differentiating sacral chordoma from sacral giant cell tumor represented AUC of 0.984 in the validation group (30). Future researches with more feature-selection methods and machine-learning classifiers are required to verify our results and to explore the optimal model with higher reliability.

There were some limitations in the present study. First, this study was performed in a single center, and only patients with resectable tumors were enrolled. Second, the study cohort, especially the testing cohort, was relatively small, which was a common limitation of other similar studies. Multicenter studies with larger sample sizes are required to validate our results. Third, only the contrast-enhanced T1-weighted imaging was used in radiomic analysis considering that this sequence was most suitable and available for the evaluation of lesions in the anterior skull base. Multi-model imaging statistics need to be integrated into the model to improve its performance in future studies. Fourth, images acquired from different MR scanners may possibly result in the model performance discrepancy. Standardized imaging quality and consistent protocols are required if the predictive models are put into clinical work.

In conclusion, we utilized MRI radiomics and clinical parameters to build predictive models *via* the combinations of selection methods and machine-learning classifiers. Our results indicated that radiomics-based machine learning could preoperatively differentiate common lesions in the anterior skull base with feasible diagnostic performance and facilitate clinical decision making.

## Data Availability Statement

The raw data supporting the conclusions of this article will be made available by the authors, without undue reservation, to any qualified researcher.

## Ethics Statement

The studies involving human participants were reviewed and approved by Ethics Committee of Sichuan University. The written informed consent was obtained from participants enrolled in this study.

## Author Contributions

YZ participated in data interpretation and feature illustration, and revised the manuscript for important intellectual content. LS participated in image evaluation and manuscript revision. CC collected MR images, participated in feature extraction, and drafted the manuscript. XM participated in conceptualization and revised some intellectual content in manuscript. XO collected MR images and participated in feature extraction. JW deployed the machine-learning algorithm and participated in statistical analysis. FX assisted in feature extraction and data collection. JX participated in conceptualization and revised some intellectual content in manuscript. All authors read and approved the submitted version.

## Conflict of Interest

The authors declare that the research was conducted in the absence of any commercial or financial relationships that could be construed as a potential conflict of interest.

## Supplementary Material

Supplementary Material 1 Explanations and formulas of radiomic features.

Supplementary Material 2 Original data of extracted radiomic features.

Supplementary Material 3 Examples of ROI delineation in MR images.
