# Supplementary material for: Machine-Learning Classifiers in Discrimination of Lesions Located in the Anterior Skull Base
Source: Front Oncol. 2020 May 28;10:752. doi: 10.3389/fonc.2020.00752 (PMC7270197; doi:10.3389/fonc.2020.00752)
Supplement: Supplementary Material 1 — Explanations and formulas of radiomic features. [file Data_Sheet_1.PDF]

Supplementary material 1: Explanations and formulas of radiomic features.

Radiomic features from the first order:

Feature	Description	Formula
HISTO_Skewness	Asymmetry of the grey-level distribution in the histogram	$HISTO\_Skewness = \frac{\frac{1}{E}\sum_i (HISTO(i) - \overline{HISTO})^3}{(\sqrt{\frac{1}{E}\sum_i (HISTO(i) - \overline{HISTO})^2})^3}$
HISTO_Kurtosis	Shape of the grey-level distribution relative to a normal distribution	$HISTO\_Kurtosis = \frac{\frac{1}{E}\sum_i (HISTO(i) - \overline{HISTO})^4}{(\frac{1}{E}\sum_i (HISTO(i) - \overline{HISTO})^2)^2}$
HISTO_Entropy_log10	Randomness of the distribution	$HISTO\_Entropy_{log10} = -\sum_i p(i) \cdot log_{10}(p(i) + \varepsilon)$
HISTO_Energy	Uniformity of the distribution	$HISTO\_Energy = \sum_i p(i)^2$
SHAPE_Volume	Volume of Interest in mL and in voxels	$SHAPE\_Volume = \sum_i V_i$

E: the total number of voxels in the Volume of Interest; $HISTO(i)$: the number of voxels with intensity I; $\overline{HISTO}$: the average of grey-levels in the histogram; $p(i)$: the probability of occurrence of voxels with intensity I; $\varepsilon = 2e - 16$; V_i: the volume of voxel i of the Volume Of Interest.

Radiomic features from grey level co-occurrence matrix (GLCM):

Feature	Description	Formula
GLCM	Takes into account the arrangements of pairs of voxels to calculate textural indices	$GLCM_{\Delta x,\Delta y}(i,j) = \frac{1}{Pairs_{ROI}} \sum_{p=1}^{N-\Delta x} \sum_{q=1}^{M-\Delta y} \begin{cases} 1 \text{ if } (I(p,q) = i, I(p+\Delta x, q+\Delta y) = j) \\ \text{and } I(p,q), I(p+\Delta x, q+\Delta y) \in ROI \\ 0 \text{ otherwise} \end{cases}$
Homogeneity	Homogeneity of grey-level voxel pairs	$GLCM_{Homogeneity} = Average\ over\ 13\ directions(\sum_i \sum_j \frac{GLCM(i,j)}{1+\lvert i-j\rvert})$
Energy	Uniformity of grey-level voxel pairs	$GLCM_{Energy} = Average\ over\ 13\ directions(\sum_i \sum_j GLCM(i,j)^2)$
Contrast	Local variations in the GLCM	$GLCM_{Contrast} = Average\ over\ 13\ directions(\sum_i \sum_j (i-j)^2 \cdot GLCM(i,j))$
Correlation	Linear dependency of grey-levels in GLCM	$GLCM_{Correlation} = Average\ over\ 13\ directions(\sum_i \sum_j \frac{(i-\mu_i)\cdot(j-\mu_j)\cdot GLCM(i,j)}{\sigma_i \cdot \sigma_j})$
Entropy_log10	Randomness of grey-level voxel pairs	$GLCM_{Entropy_{log10}} = Average\ over\ 13\ directions(-\sum_i \sum_j GLCM(i,j)\cdot log_{10}(GLCM(i,j)+\epsilon))$
Dissimilarity	Variation of grey-level voxel pairs	$GLCM_{Dissimilarity} = Average\ over\ 13\ directions(\sum_i \sum_j \lvert i-j\rvert \cdot GLCM(i,j))$

$I(p,q)$ corresponds to voxel(p,q) in an image (I) of size $N{*}M$; $Pairs_{ROI}$ corresponds to the number of all voxel pairs belonging to the region of interest (ROI); μ_i: the average on row i; μ_j: the average on column j; σ_i: the variance on row i; σ_j: the variance on column j; $\varepsilon = 2e-16$.

Radiomic features from grey-level zone length matrix (GLZLM):

Feature	Description	Formula
GLZLM_SZE<br>(short-zone emphasis)	Distribution of the short homogeneous zones in an image	$GLZLM\_SZE = \frac{1}{H}\sum_{i}\sum_{j}\frac{GLZLM(i,j)}{j^2}$
GLZLM_LZE<br>(long-zone emphasis)	Distribution of the long homogeneous zones in an image	$GLZLM\_LZE = \frac{1}{H}\sum_{i}\sum_{j} GLZLM(i,j)\cdot j^2$
GLZLM_LGZE<br>(low grey-level zone emphasis)	Distribution of the low grey-level zones.	$GLZLM\_LGZE = \frac{1}{H}\sum_{i}\sum_{j}\frac{GLZLM(i,j)}{i^2}$
GLZLM_HGZE<br>(high grey-level zone emphasis)	Distribution of the high grey-level zones	$GLZLM\_HGZE = \frac{1}{H}\sum_{i}\sum_{j} GLZLM(i,j)\cdot i^2$
GLZLM_SZLGE<br>(short-zone low grey-level emphasis)	Distribution of the short homogeneous zones with low grey-levels	$GLZLM\_SZLGE = \frac{1}{H}\sum_{i}\sum_{j}\frac{GLZLM(i,j)}{i^2\cdot j^2}$
GLZLM_SZHGE<br>(short-zone high grey-level emphasis)	Distribution of the short homogeneous zones with high grey-levels	$GLZLM\_SZHGE = \frac{1}{H}\sum_{i}\sum_{j}\frac{GLZLM(i,j)\cdot i^2}{j^2}$
GLZLM_LZLGE<br>(long-zone low grey-level emphasis)	Distribution of the long homogeneous zones with low grey-levels	$GLZLM\_LZLGE = \frac{1}{H}\sum_{i}\sum_{j}\frac{GLZLM(i,j)\cdot j^2}{i^2}$
GLZLM_LZHGE<br>(long-zone high grey-level emphasis)	Distribution of the long homogeneous zones with high grey-levels	$GLZLM\_LZHGE = \frac{1}{H}\sum_{i}\sum_{j} GLZLM(i,j)\cdot i^2\cdot j^2$
GLZLM_GLNUz<br>(grey-level non-uniformity for zone)	Non-uniformity of the grey-levels of the homogeneous zones	$GLZLM\_GLNUz = \frac{1}{H}\sum_{i}(\sum_{j} GLZLM(i,j))^2$
GLZLM_ZLNU<br>(zone length non-uniformity)	Non-uniformity of the length of the homogeneous zones	$GLZLM\_ZLNU = \frac{1}{H}\sum_{j}(\sum_{i} GLZLM(i,j))^2$
GLZLM_ZP (zone percentage)	Homogeneity of the homogeneous zones	$GLZLM\_ZP = \frac{H}{\sum_i \sum_j (j\cdot GLZLM(i,j))}$

$GLZLM(i,j)$: the number of homogeneous zones of j voxels with intensity i in an image; H: the number of homogeneous zones in the Volume of Interest.

Radiomic features from neighborhood grey-level different matrix (NGLDM):

Feature	Description	Formula
NGLDM	Difference of grey-level between one voxel and its 26 neighbours in 3 dimensions	$NGLDM(i,2) = \sum_p \sum_q \{ \|\overline{M}(p,q) - i\| \text{~if } I(p,q) = i; \ 0\text{~else}$
Coarseness	Level of spatial rate of change in intensity	$NGLDM\_Coarseness = \frac{1}{\sum_i NGLDM(i,1) \cdot NGLDM(i,2)}$
Contrast	Intensity difference between neighbouring regions	$NGLDM\_Contrast = [\sum_i \sum_j NGLDM(i,1) \cdot NGLDM(j,1) \cdot (i-j)^2] \cdot \frac{\sum_i NGLDM(i,2)}{E \cdot G \cdot (G-1)}$
Busyness	Spatial frequency of changes in intensity	$NGLDM\_Busyness = \frac{\sum_i NGLDM(i,1) \cdot NGLDM(i,2)}{\sum_i \sum_j \|i \cdot NGLDM(i,1) - j \cdot NGLDM(j,1)\|}$ $with\ NGLDM(i,1) \neq 0, NGLDM(j,1) \neq 0$

$\overline{M}(p,q)$: the average of intensities over the 26 neighbour voxels of voxel (p,q); E: the number of voxels in the Volume of Interest; G: the number of grey-levels.

Radiomic features from grey-level run length matrix (GLRLM):

Feature	Description	Formula
GLRLM_SRE (short-run emphasis)	Distribution of the short homogeneous runs in an image	$GLRLM_{SRE} = Average\ over\ 13\ directions(\frac{1}{H}\sum_{i}\sum_{j}\frac{GLRLM(i,j)}{j^2})$
GLRLM_LRE (long-run emphasis)	Distribution of the long homogeneous runs in an image	$GLRLM_{LRE} = Average\ over\ 13\ directions(\frac{1}{H}\sum_{i}\sum_{j} GLRLM(i,j) \cdot j^2)$
GLRLM_LGRE (low grey-level run emphasis)	Distribution of the low grey-level runs	$GLRLM_{LGRE} = Average\ over\ 13\ directions(\frac{1}{H}\sum_{i}\sum_{j}\frac{GLRLM(i,j)}{i^2})$
GLRLM_HGRE (high grey-level run emphasis)	Distribution of the high grey-level runs	$GLRLM_{HGRE} = Average\ over\ 13\ directions(\frac{1}{H}\sum_{i}\sum_{j} GLRLM(i,j) \cdot i^2)$
GLRLM_SRLGE (short-run low grey-level)	Distribution of the short homogeneous runs with low grey-levels	$GLRLM_{SRLGE} = Average\ over\ 13\ directions(\frac{1}{H}\sum_{i}\sum_{j}\frac{GLRLM(i,j)}{i^2 \cdot j^2})$
GLRLM_SRHGE (short-run high grey-level)	Distribution of the short homogeneous runs with high grey-levels	$GLRLM_{SRHGE} = Average\ over\ 13\ directions(\frac{1}{H}\sum_{i}\sum_{j}\frac{GLRLM(i,j) \cdot i^2}{j^2})$
GLRLM_LRLGE (long-run low grey-level)	Distribution of the long homogeneous runs with low grey-levels	$GLRLM_{LRLGE} = Average\ over\ 13\ directions(\frac{1}{H}\sum_{i}\sum_{j}\frac{GLRLM(i,j) \cdot j^2}{i^2})$
GLRLM_LRHGE (long-run high grey-level)	Distribution of the long homogeneous runs with high grey-levels	$GLRLM_{LRHGE} = Average\ over\ 13\ directions(\frac{1}{H}\sum_{i}\sum_{j} GLRLM(i,j) \cdot i^2 \cdot j^2)$
GLRLM_GLNUr (grey-level non-uniformity for run)	Non-uniformity of the grey-levels of the homogeneous runs	$GLRLM_{GLNUr} = Average\ over\ 13\ directions(\frac{1}{H}\sum_{i}(\sum_{j} GLRLM(i,j))^2)$
GLRLM_RLNU (run length non-uniformity)	Non-uniformity of the length of the homogeneous runs	$GLRLM_{RLNU} = Average\ over\ 13\ directions(\frac{1}{H}\sum_{j}(\sum_{i} GLRLM(i,j))^2)$
GLRLM_RP (run percentage)	Homogeneity of the homogeneous runs	$GLRLM_{RP} = Average\ over\ 13\ directions(\frac{H}{\sum_i \sum_j (j \cdot GLRLM(i,j))})$

$GLRLM(i,j)$: the number of homogeneous runs of j voxels with intensity i in an image; H: the number of homogeneous runs in the Volume of Interest.
