# Supplementary material for: Machine-Learning Classifiers in Discrimination of Lesions Located in the Anterior Skull Base
Source: Front Oncol. 2020 May 28;10:752. doi: 10.3389/fonc.2020.00752 (PMC7270197; doi:10.3389/fonc.2020.00752)
Supplement: Supplementary Material 2 — Original data of extracted radiomic features. [file Data_Sheet_2.PDF]

Patients with pituitary adenoma	1	2	3	4	5	6	7	8	9	10
minVaule	33	149	119	5	53	86	11	167	77	187
meanValue	641	413	675	505	871	577	327	918	692	382
stdValue	127	78	83.7	87.8	138	98.6	58.8	182	113	75.8
maxValue	1500	783	1250	926	1480	1200	511	1900	1050	661
HISTO_Skewness	0.377	0.176	-0.193	-0.381	0.419	-0.0836	-1.19	0.804	-0.49	0.535
HISTO_Kurtosis	4.84	4.06	5.4	4.25	5.79	4.4	6.56	4.51	3.92	2.78
HISTO_Entropy_log10	1.35	1.49	1.28	1.37	1.38	1.36	1.45	1.41	1.48	1.59
HISTO_Energy	0.0549	0.0399	0.0653	0.0528	0.0538	0.0546	0.0453	0.0474	0.0392	0.0299
SHAPE_Volume	4.38	4.1	9.25	6.86	4.35	8.01	0.84	9.43	2.67	1
GLCM_Homogeneity	0.331	0.328	0.467	0.427	0.313	0.377	0.333	0.325	0.292	0.23
GLCM_Energy	0.00417	0.0036	0.0101	0.0071	0.00412	0.00536	0.00407	0.00382	0.00249	0.00193
GLCM_Contrast	27	41.4	12.7	19.5	39.3	22.6	40.8	42.5	41.1	101
GLCM_Correlation	0.51	0.626	0.655	0.713	0.413	0.617	0.55	0.458	0.578	0.473
GLCM_Entropy_log10	2.55	2.7	2.25	2.45	2.58	2.51	2.6	2.63	2.72	2.81
GLCM_Dissimilarity	3.88	4.62	2.36	2.94	4.42	3.35	4.4	4.63	4.78	7.47
GLRLM_SRE	0.94	0.935	0.867	0.888	0.946	0.919	0.94	0.935	0.954	0.97
GLRLM_LRE	1.28	1.45	2.11	1.92	1.25	1.49	1.35	1.35	1.21	1.13
GLRLM_LGRE	0.00184	0.00212	0.00114	0.00101	0.00107	0.00162	0.00169	0.00165	0.0011	0.0034
GLRLM_HGRE	763	800	1050	1280	1430	857	1730	852	1720	830
GLRLM_SRLGE	0.00175	0.002	0.00101	0.000908	0.00103	0.0015	0.00163	0.00155	0.00107	0.00333
GLRLM_SRHGE	719	748	910	1140	1360	787	1620	803	1640	811
GLRLM_LRLGE	0.00226	0.00292	0.00228	0.00183	0.00127	0.00234	0.00201	0.00221	0.00123	0.00372
GLRLM_LRHGE	965	1160	2200	2440	1780	1270	2350	1110	2080	911
GLRLM_GLNUr	226	360	2370	1840	217	2380	215	1150	98.9	29
GLRLM_RLNU	3560	7970	28100	28300	3580	36600	4210	21100	2250	908
GLRLM_RP	0.921	0.907	0.819	0.842	0.927	0.887	0.917	0.913	0.939	0.96
NGLDM_Coarseness	0.00187	0.000876	0.00023	0.000259	0.00183	0.000199	0.00172	0.000281	0.00339	0.00799
NGLDM_Contrast	0.0642	0.108	0.0224	0.0401	0.0707	0.045	0.0843	0.0782	0.123	0.338
NGLDM_Busyness	0.339	0.55	1.48	1.3	0.213	2.17	0.159	1.49	0.134	0.11
GLZLM_SZE	0.663	0.58	0.529	0.565	0.669	0.579	0.634	0.611	0.689	0.768
GLZLM_LZE	88.5	77	14900	5080	88.9	1000	48.3	114	22.4	5.98
GLZLM_LGZE	0.00242	0.0026	0.00179	0.00131	0.00158	0.00193	0.00246	0.0018	0.00166	0.00403
GLZLM_HGZE	800	817	1060	1220	1490	878	1620	970	1690	940
GLZLM_SZLGE	0.00189	0.00174	0.00118	0.000728	0.00132	0.00112	0.00167	0.00118	0.00146	0.00354
GLZLM_SZHGE	536	477	554	673	1000	517	972	610	1140	760
GLZLM_LZLGE	0.136	0.118	15.1	4.52	0.0686	1.2	0.034	0.199	0.0149	0.0173
GLZLM_LZHGE	60900	62500	15000000	5880000	119000	859000	88400	69900	38300	3660
GLZLM_GLNUz	61.1	92	169	230	59.4	413	56.4	313	38.8	15.8
GLZLM_ZLNU	597	924	1190	1910	653	3380	665	2930	507	327
GLZLM_ZP	0.324	0.288	0.0975	0.147	0.355	0.214	0.336	0.311	0.427	0.583

Patients with pituitary adenoma	11	12	13	14	15	16	17	18	19	20
minVaule	179	151	131	223	136	34	92	118	26	250
meanValue	614	573	386	850	636	450	477	450	776	688
stdValue	204	153	96	167	162	87.5	89.5	167	105	95.6
maxValue	1020	1270	825	1620	1440	1170	998	1100	1580	1080
HISTO_Skewness	-0.21	0.112	0.857	0.498	0.242	0.524	0.53	0.257	-0.0366	-0.00729
HISTO_Kurtosis	1.91	3.13	3.33	4.38	3.56	4.69	4.38	2	6.47	4.29
HISTO_Entropy_log10	1.75	1.54	1.51	1.48	1.5	1.3	1.41	1.57	1.23	1.47
HISTO_Energy	0.019	0.0326	0.0391	0.0413	0.039	0.0589	0.0469	0.0311	0.071	0.0424
SHAPE_Volume	0.922	2.65	2.78	10.6	4.48	8.89	5.23	6.21	11.5	1.06
GLCM_Homogeneity	0.265	0.282	0.362	0.35	0.35	0.425	0.311	0.337	0.491	0.278
GLCM_Energy	0.00121	0.00186	0.00453	0.00388	0.00342	0.00718	0.00331	0.00336	0.0112	0.00352
GLCM_Contrast	99.9	46	40.5	44.7	40.9	14.2	39.3	54.4	8.44	60.1
GLCM_Correlation	0.811	0.687	0.696	0.569	0.661	0.682	0.463	0.774	0.745	0.273
GLCM_Entropy_log10	3.08	2.83	2.67	2.66	2.72	2.34	2.66	2.82	2.16	2.63
GLCM_Dissimilarity	7.1	5.11	4.32	4.5	4.46	2.69	4.62	5.11	1.98	5.77
GLRLM_SRE	0.958	0.956	0.916	0.93	0.922	0.891	0.945	0.925	0.855	0.955
GLRLM_LRE	1.25	1.2	1.62	1.44	1.55	1.84	1.28	1.57	2.2	1.24
GLRLM_LGRE	0.00466	0.00369	0.00296	0.00185	0.00267	0.00205	0.00163	0.00474	0.00123	0.00155
GLRLM_HGRE	1340	691	675	927	700	598	811	629	1010	1240
GLRLM_SRLGE	0.00447	0.00355	0.00268	0.00173	0.00246	0.00183	0.00154	0.00419	0.00106	0.00151
GLRLM_SRHGE	1280	663	631	869	650	535	769	593	861	1190
GLRLM_LRLGE	0.00601	0.00434	0.00494	0.00257	0.00445	0.00372	0.00206	0.00947	0.00255	0.00178
GLRLM_LRHGE	1720	813	996	1270	1030	1080	1020	861	2220	1530
GLRLM_GLNUr	101	83.2	583	316	974	2200	794	1020	4040	102
GLRLM_RLNU	4870	2270	13000	6670	21000	29500	14900	28900	41800	2210
GLRLM_RP	0.939	0.941	0.881	0.903	0.888	0.848	0.927	0.888	0.803	0.939
NGLDM_Coarseness	0.0024	0.00358	0.000613	0.00115	0.000339	0.000223	0.000429	0.000251	0.000158	0.00285
NGLDM_Contrast	0.481	0.157	0.116	0.0887	0.103	0.0245	0.0634	0.215	0.0128	0.129
NGLDM_Busyness	0.225	0.172	0.97	0.388	1.52	2.12	0.989	2.79	1.94	0.164
GLZLM_SZE	0.663	0.686	0.607	0.617	0.569	0.509	0.611	0.611	0.553	0.654
GLZLM_LZE	17.2	17.6	621	79	288	4820	30.4	1110	35900	12.8
GLZLM_LGZE	0.00439	0.00445	0.00257	0.00214	0.00253	0.00232	0.00174	0.00307	0.00211	0.00221
GLZLM_HGZE	1190	743	848	1060	775	658	868	721	944	1270
GLZLM_SZLGE	0.00261	0.00361	0.00136	0.00125	0.00141	0.00126	0.00112	0.00182	0.00134	0.00186
GLZLM_SZHGE	745	526	525	679	448	347	547	438	501	837
GLZLM_LZLGE	0.0884	0.0668	1.82	0.125	0.937	9.41	0.0498	13.7	36.3	0.0131
GLZLM_LZHGE	26600	10100	228000	55800	145000	2640000	21300	116000	36400000	15400
GLZLM_GLNUz	48.9	38.6	131	80.8	223	222	266	327	222	39.1
GLZLM_ZLNU	1020	522	1520	948	1980	1190	2340	3840	1550	453
GLZLM_ZP	0.438	0.443	0.248	0.309	0.231	0.109	0.364	0.284	0.0744	0.438

Patients with pituitary adenoma	21	22	23	24	25	26	27	28	29	30
minVaule	145	122	76	71	115	38	9	193	59	110
meanValue	324	930	617	491	557	599	476	579	391	423
stdValue	47.2	191	120	70.6	88.5	201	105	56	73.3	71.1
maxValue	652	1760	1410	1200	1100	1140	956	1200	694	647
HISTO_Skewness	0.816	0.32	0.913	0.4	0.349	-0.487	0.0571	-0.234	-0.314	-0.545
HISTO_Kurtosis	3.46	3.7	5.95	8.24	3.9	2.17	3	8.64	3.86	4
HISTO_Entropy_log10	1.29	1.48	1.35	1.2	1.37	1.6	1.46	1.12	1.47	1.51
HISTO_Energy	0.0712	0.0406	0.0574	0.0803	0.0518	0.0293	0.0393	0.103	0.041	0.0402
SHAPE_Volume	13.4	22.2	8.74	6.44	5.53	8.64	4.94	13.1	4.66	0.618
GLCM_Homogeneity	0.427	0.355	0.4	0.475	0.34	0.288	0.339	0.541	0.338	0.336
GLCM_Energy	0.0128	0.00373	0.00644	0.0129	0.00429	0.00172	0.00339	0.0227	0.00326	0.00366
GLCM_Contrast	22.2	33.1	18.7	10.2	30.6	65.7	34.2	6.64	34.2	37.5
GLCM_Correlation	0.636	0.663	0.633	0.621	0.482	0.764	0.617	0.663	0.631	0.705
GLCM_Entropy_log10	2.3	2.69	2.42	2.13	2.56	2.96	2.65	1.94	2.69	2.7
GLCM_Dissimilarity	3.05	4.03	3.01	2.16	4.01	5.7	4.28	1.65	4.17	4.37
GLRLM_SRE	0.896	0.919	0.909	0.865	0.932	0.948	0.927	0.817	0.934	0.934
GLRLM_LRE	1.64	1.49	1.52	2.13	1.39	1.24	1.5	2.88	1.38	1.38
GLRLM_LGRE	0.00231	0.00122	0.0018	0.00207	0.00139	0.00184	0.00178	0.00183	0.0012	0.00162
GLRLM_HGRE	587	1100	746	610	888	1240	1080	647	1210	1500
GLRLM_SRLGE	0.00206	0.00113	0.00164	0.00183	0.0013	0.00173	0.00164	0.00152	0.00114	0.00155
GLRLM_SRHGE	537	1020	684	529	830	1180	1000	529	1130	1400
GLRLM_LRLGE	0.00388	0.0018	0.00267	0.00419	0.00192	0.00241	0.00274	0.00495	0.00159	0.002
GLRLM_LRHGE	885	1590	1100	1280	1210	1500	1610	1840	1670	2070
GLRLM_GLNUr	975	2660	1440	2530	1600	269	1080	4720	1070	136
GLRLM_RLNU	11900	55600	20700	24200	26600	8070	23400	33200	22500	2990
GLRLM_RP	0.858	0.891	0.879	0.816	0.906	0.93	0.897	0.757	0.909	0.908
NGLDM_Coarseness	0.000477	0.000127	0.000332	0.000244	0.00024	0.00107	0.000281	0.000156	0.000343	0.0028
NGLDM_Contrast	0.0536	0.0828	0.0371	0.013	0.0526	0.258	0.0843	0.00914	0.0781	0.107
NGLDM_Busyness	1.24	3.09	1.24	1.68	1.7	0.41	1.42	2.58	1	0.123
GLZLM_SZE	0.621	0.592	0.59	0.566	0.581	0.718	0.539	0.562	0.597	0.635
GLZLM_LZE	3300	783	1890	20100	153	53.4	166	84600	106	71.6
GLZLM_LGZE	0.00241	0.00135	0.00215	0.00335	0.00153	0.00169	0.00196	0.00315	0.00157	0.00226
GLZLM_HGZE	700	1200	887	618	961	1300	1110	667	1190	1510
GLZLM_SZLGE	0.00167	0.000855	0.00136	0.00219	0.000983	0.00129	0.00113	0.00215	0.00109	0.00131
GLZLM_SZHGE	434	710	545	359	564	913	594	368	702	957
GLZLM_LZLGE	8.83	1.02	3.11	35.1	0.211	0.186	0.226	134	0.0935	0.0539
GLZLM_LZHGE	1250000	630000	1210000	11800000	117000	40500	165000	54200000	132000	111000
GLZLM_GLNUz	173	578	226	139	372	113	248	156	277	36.1
GLZLM_ZLNU	1480	6240	1940	1040	2890	1980	1950	1160	2730	475
GLZLM_ZP	0.228	0.252	0.202	0.0891	0.263	0.418	0.23	0.0604	0.284	0.328

Patients with pituitary adenoma	31	32	33	34	35	36	37	38	39	40
minVaule	94	92	37	29	105	52	83	207	264	65
meanValue	249	326	658	383	407	137	666	399	511	592
stdValue	61.7	58.8	102	74.9	167	42.3	184	52.4	70.5	93
maxValue	452	684	1280	733	1140	405	1290	664	748	982
HISTO_Skewness	0.0243	0.905	0.516	-0.177	0.907	1.67	0.0673	0.706	-0.352	0.588
HISTO_Kurtosis	1.78	4.31	6.06	3.77	2.98	4.97	2.64	4.79	3.53	5.01
HISTO_Entropy_log10	1.55	1.37	1.31	1.44	1.51	1.23	1.6	1.46	1.57	1.39
HISTO_Energy	0.0333	0.0557	0.0614	0.0433	0.0404	0.101	0.0283	0.0444	0.0322	0.0542
SHAPE_Volume	8.3	3.14	15.6	7.13	5.08	13.5	1.96	1.25	0.546	5.25
GLCM_Homogeneity	0.366	0.386	0.343	0.331	0.298	0.527	0.243	0.324	0.264	0.366
GLCM_Energy	0.00403	0.00658	0.00573	0.00344	0.00366	0.0296	0.00141	0.00373	0.00215	0.00532
GLCM_Contrast	54.5	23.1	29.3	32.4	59.2	21.4	109	47.3	92.4	28
GLCM_Correlation	0.774	0.675	0.37	0.594	0.707	0.765	0.41	0.44	0.368	0.582
GLCM_Entropy_log10	2.76	2.46	2.45	2.65	2.77	2.06	2.99	2.66	2.84	2.53
GLCM_Dissimilarity	4.79	3.33	3.94	4.14	5.41	2.54	7.67	4.67	6.92	3.69
GLRLM_SRE	0.911	0.914	0.931	0.937	0.947	0.822	0.96	0.941	0.955	0.921
GLRLM_LRE	1.68	1.57	1.38	1.34	1.25	3.07	1.19	1.35	1.27	1.47
GLRLM_LGRE	0.00213	0.00198	0.00115	0.00124	0.00612	0.00589	0.00192	0.00197	0.00399	0.000863
GLRLM_HGRE	953	717	1090	1110	492	362	1090	810	1190	1440
GLRLM_SRLGE	0.00188	0.00182	0.00107	0.00118	0.00575	0.00461	0.00184	0.00187	0.00382	0.000801
GLRLM_SRHGE	887	661	1020	1040	476	325	1050	765	1130	1330
GLRLM_LRLGE	0.00426	0.00303	0.00156	0.00158	0.0079	0.0212	0.00228	0.00252	0.00495	0.00123
GLRLM_LRHGE	1400	1070	1480	1510	562	725	1280	1080	1510	2100
GLRLM_GLNUr	1410	916	2220	622	207	4850	180	309	102	1540
GLRLM_RLNU	36700	14000	31400	12500	4710	41800	5800	6170	2870	24000
GLRLM_RP	0.871	0.88	0.907	0.914	0.929	0.743	0.947	0.917	0.936	0.891
NGLDM_Coarseness	0.000191	0.000487	0.000175	0.000605	0.00154	0.000108	0.00109	0.00117	0.00221	0.000277
NGLDM_Contrast	0.207	0.0497	0.0394	0.0648	0.199	0.0635	0.255	0.0934	0.219	0.0517
NGLDM_Busyness	2.63	0.926	1.88	0.577	0.541	7.49	0.43	0.39	0.183	1.04
GLZLM_SZE	0.607	0.578	0.59	0.6	0.737	0.586	0.667	0.648	0.661	0.591
GLZLM_LZE	2220	880	154	79.4	133	53600	9.15	98.2	17.9	654
GLZLM_LGZE	0.00147	0.00262	0.00124	0.00167	0.00537	0.00295	0.00196	0.00247	0.00447	0.0011
GLZLM_HGZE	1150	833	1170	1050	682	671	1150	879	1180	1520
GLZLM_SZLGE	0.000882	0.00193	0.000735	0.0012	0.0041	0.00151	0.00124	0.00178	0.003	0.000765
GLZLM_SZHGE	703	482	704	618	528	406	787	595	771	903
GLZLM_LZLGE	9.24	1.73	0.16	0.0672	1.04	384	0.0179	0.137	0.0389	0.51
GLZLM_LZHGE	611000	462000	152000	101000	19600	7640000	8260	75100	21700	850000
GLZLM_GLNUz	353	150	511	172	63.5	363	92.3	86.5	39.8	264
GLZLM_ZLNU	4190	1290	3780	1620	1210	3540	1380	1030	581	2390
GLZLM_ZP	0.236	0.211	0.284	0.303	0.412	0.13	0.496	0.342	0.421	0.226

Patients with pituitary adenoma	41	42	43	44	45	46	47	48	49	50
minVaule	36	1	10	33	39	141	146	178	183	11
meanValue	270	209	304	281	400	459	542	345	602	436
stdValue	36.7	85.4	48.6	133	70.8	97	111	41.1	108	73.4
maxValue	453	632	551	792	869	914	1100	848	1080	915
HISTO_Skewness	-0.0879	0.751	0.147	0.785	-0.00746	0.502	-0.0368	4.67	0.893	0.274
HISTO_Kurtosis	4.41	3.34	5.08	4.31	4.47	3.69	4.37	41.3	4.17	6.51
HISTO_Entropy_log10	1.36	1.52	1.35	1.6	1.34	1.51	1.47	0.998	1.46	1.29
HISTO_Energy	0.0535	0.0354	0.0568	0.0287	0.0552	0.0366	0.0434	0.144	0.042	0.0682
SHAPE_Volume	5.45	6.8	5.31	0.495	2.3	1.93	2.49	3.28	1.39	6.57
GLCM_Homogeneity	0.349	0.312	0.384	0.206	0.363	0.317	0.321	0.527	0.267	0.428
GLCM_Energy	0.00462	0.0026	0.00614	0.00262	0.00574	0.00271	0.00339	0.0328	0.00277	0.00921
GLCM_Contrast	27.7	41.1	24.9	162	26.3	48.7	32.5	7.71	54.4	18.5
GLCM_Correlation	0.514	0.704	0.551	0.392	0.479	0.582	0.663	0.387	0.503	0.565
GLCM_Entropy_log10	2.54	2.8	2.47	2.64	2.46	2.77	2.65	1.76	2.68	2.32
GLCM_Dissimilarity	3.82	4.68	3.42	9.1	3.73	4.86	4.16	1.73	5.54	2.87
GLRLM_SRE	0.928	0.942	0.915	0.978	0.92	0.94	0.947	0.844	0.961	0.889
GLRLM_LRE	1.41	1.31	1.54	1.09	1.56	1.35	1.25	2.3	1.17	1.83
GLRLM_LGRE	0.000905	0.00393	0.000928	0.0203	0.00152	0.00212	0.00359	0.00447	0.00202	0.00135
GLRLM_HGRE	1360	551	1280	586	835	792	787	293	995	969
GLRLM_SRLGE	0.000845	0.00369	0.000855	0.0201	0.00142	0.002	0.00349	0.00383	0.00197	0.00122
GLRLM_SRHGE	1260	525	1170	574	767	750	746	253	962	866
GLRLM_LRLGE	0.00125	0.00524	0.00139	0.0215	0.00228	0.00283	0.00401	0.00977	0.00226	0.00232
GLRLM_LRHGE	1910	683	1950	634	1290	1040	978	627	1140	1730
GLRLM_GLNUr	1670	483	1620	14	695	400	102	2100	55.7	2260
GLRLM_RLNU	26600	12000	23900	465	10800	9580	2080	11200	1210	26800
GLRLM_RP	0.901	0.921	0.883	0.971	0.888	0.917	0.928	0.788	0.947	0.845
NGLDM_Coarseness	0.000238	0.000713	0.000274	0.0157	0.000609	0.00077	0.00391	0.000373	0.00531	0.000233
NGLDM_Contrast	0.045	0.139	0.0454	0.451	0.0475	0.108	0.099	0.00986	0.196	0.0271
NGLDM_Busyness	1.36	1.11	1.4	0.0641	0.791	0.626	0.131	1.46	0.209	1.43
GLZLM_SZE	0.568	0.626	0.584	0.802	0.573	0.606	0.686	0.618	0.725	0.595
GLZLM_LZE	188	67.1	1070	3.09	400	61.8	33.5	21800	13.8	7550
GLZLM_LGZE	0.00105	0.00365	0.0011	0.0232	0.00194	0.00224	0.0061	0.00609	0.00266	0.00197
GLZLM_HGZE	1380	674	1320	607	841	898	809	488	1100	1050
GLZLM_SZLGE	0.000599	0.00206	0.000714	0.0208	0.00129	0.00129	0.00538	0.0041	0.00232	0.00127
GLZLM_SZHGE	782	440	779	503	485	573	563	357	819	608
GLZLM_LZLGE	0.149	0.312	0.854	0.0491	0.494	0.125	0.0526	87.2	0.0214	8.79
GLZLM_LZHGE	247000	19100	1370000	1730	335000	38300	26500	5510000	10700	6560000
GLZLM_GLNUz	351	160	263	9.45	130	121	31.6	94.4	24.3	213
GLZLM_ZLNU	2620	1920	2240	210	965	1330	432	733	338	2040
GLZLM_ZP	0.245	0.348	0.212	0.69	0.217	0.321	0.385	0.101	0.488	0.151

Patients with pituitary adenoma	51	52	53	54	55	56	57	58	59	60
minVaule	184	83	35	48	42	36	90	105	34	259
meanValue	634	517	986	642	767	735	720	611	414	670
stdValue	142	96.6	211	153	376	189	114	154	87.8	73
maxValue	1010	892	2130	1190	1420	1530	1460	1180	933	1010
HISTO_Skewness	-0.564	0.234	-1.1	0.0714	-0.644	0.98	-0.593	-0.0756	1.01	0.319
HISTO_Kurtosis	3.15	3.98	5.77	2.89	2.08	4.42	4.89	4.42	5.2	4.83
HISTO_Entropy_log10	1.57	1.48	1.36	1.54	1.62	1.47	1.33	1.52	1.37	1.39
HISTO_Energy	0.0341	0.0419	0.0577	0.0335	0.0286	0.044	0.0559	0.0417	0.0517	0.0508
SHAPE_Volume	0.586	0.994	36.3	3.34	0.382	1.24	13	2.94	7.52	3.73
GLCM_Homogeneity	0.273	0.334	0.327	0.294	0.255	0.328	0.337	0.29	0.352	0.293
GLCM_Energy	0.00377	0.00372	0.0043	0.00206	0.0035	0.0043	0.00428	0.00306	0.00469	0.00348
GLCM_Contrast	51.5	47.6	30.2	39.5	148	51.8	22.5	49.7	26.1	37.9
GLCM_Correlation	0.649	0.538	0.637	0.722	0.741	0.518	0.543	0.681	0.627	0.452
GLCM_Entropy_log10	2.53	2.68	2.59	2.82	2.53	2.55	2.51	2.76	2.54	2.63
GLCM_Dissimilarity	5.38	4.68	4.05	4.74	8.13	4.69	3.62	5.12	3.68	4.68
GLRLM_SRE	0.965	0.937	0.937	0.952	0.966	0.945	0.935	0.951	0.93	0.951
GLRLM_LRE	1.15	1.39	1.3	1.22	1.15	1.26	1.3	1.22	1.34	1.22
GLRLM_LGRE	0.00328	0.00119	0.00185	0.0014	0.0194	0.0022	0.00178	0.00329	0.00164	0.00123
GLRLM_HGRE	1350	1280	922	1230	1480	1010	922	1030	811	1310
GLRLM_SRLGE	0.00325	0.00113	0.00172	0.00135	0.019	0.00212	0.0017	0.00316	0.00153	0.00119
GLRLM_SRHGE	1300	1200	866	1180	1430	963	860	984	763	1250
GLRLM_LRLGE	0.00341	0.00154	0.00248	0.00166	0.021	0.00256	0.00214	0.00383	0.00223	0.00141
GLRLM_LRHGE	1580	1760	1180	1470	1680	1210	1210	1230	1040	1580
GLRLM_GLNUr	19	230	1990	120	10.5	50.1	751	129	393	177
GLRLM_RLNU	522	4870	29700	3180	342	1020	11500	2800	6510	3140
GLRLM_RP	0.954	0.911	0.915	0.936	0.955	0.926	0.914	0.936	0.906	0.936
NGLDM_Coarseness	0.0163	0.00144	0.000215	0.00285	0.0203	0.00751	0.000587	0.00259	0.00108	0.00199
NGLDM_Contrast	0.29	0.101	0.069	0.139	0.773	0.162	0.0533	0.157	0.055	0.0939
NGLDM_Busyness	0.0312	0.277	1.86	0.184	0.0227	0.105	0.782	0.165	0.457	0.275
GLZLM_SZE	0.758	0.623	0.679	0.666	0.735	0.745	0.625	0.697	0.659	0.692
GLZLM_LZE	7.5	75.6	669	27.4	6.32	39.4	226	41.3	377	38.2
GLZLM_LGZE	0.00501	0.00167	0.00195	0.00173	0.0257	0.00325	0.00299	0.00466	0.00183	0.00172
GLZLM_HGZE	1250	1290	962	1350	1490	1220	875	1120	960	1360
GLZLM_SZLGE	0.00473	0.00129	0.00132	0.00139	0.0232	0.00294	0.00249	0.00405	0.00138	0.00146
GLZLM_SZHGE	907	799	655	918	1070	958	556	793	648	953
GLZLM_LZLGE	0.01	0.0659	0.925	0.0347	0.0942	0.0614	0.245	0.0698	0.754	0.0329
GLZLM_LZHGE	11400	92800	566000	27700	8300	28100	221000	38800	194000	47100
GLZLM_GLNUz	8.11	59.9	460	49.7	5.3	15.9	168	39.5	101	58.3
GLZLM_ZLNU	176	718	4920	660	109	265	1510	622	1030	678
GLZLM_ZP	0.552	0.321	0.301	0.417	0.556	0.403	0.285	0.407	0.298	0.403

Patients with pituitary adenoma	61	62	63	64	65	66	67	68
minVaule	59	155	12	96	23	66	187	35
meanValue	598	763	605	604	558	511	635	841
stdValue	96.3	118	100	88.2	86	70.2	208	139
maxValue	1430	1320	970	1480	1160	858	1590	1520
HISTO_Skewness	0.705	0.589	-0.514	0.203	-0.0884	-0.488	-0.0493	0.119
HISTO_Kurtosis	6.1	5.5	4.47	6.62	5.19	4.85	1.7	6.22
HISTO_Entropy_log10	1.24	1.4	1.43	1.2	1.29	1.36	1.41	1.36
HISTO_Energy	0.0715	0.0506	0.0459	0.0829	0.0619	0.0542	0.0509	0.0563
SHAPE_Volume	11	6.99	24.5	5.63	3.32	6	14.5	2.22
GLCM_Homogeneity	0.364	0.288	0.355	0.463	0.43	0.367	0.459	0.316
GLCM_Energy	0.00667	0.00355	0.00407	0.0136	0.00828	0.00532	0.0123	0.00471
GLCM_Contrast	19.2	46.8	31.6	12.1	13.5	24.9	27.3	39.2
GLCM_Correlation	0.454	0.319	0.613	0.532	0.646	0.544	0.85	0.317
GLCM_Entropy_log10	2.36	2.62	2.63	2.14	2.29	2.49	2.43	2.51
GLCM_Dissimilarity	3.24	4.98	3.96	2.37	2.63	3.57	3.22	4.4
GLRLM_SRE	0.926	0.956	0.923	0.872	0.89	0.921	0.868	0.945
GLRLM_LRE	1.36	1.2	1.49	2.14	1.89	1.5	2.46	1.26
GLRLM_LGRE	0.00191	0.00148	0.000786	0.00207	0.00136	0.000888	0.00449	0.0014
GLRLM_HGRE	681	1190	1650	597	951	1360	572	1290
GLRLM_SRLGE	0.00178	0.00144	0.000734	0.00183	0.00124	0.000827	0.00359	0.00136
GLRLM_SRHGE	633	1140	1520	521	843	1250	514	1220
GLRLM_LRLGE	0.00251	0.00168	0.00112	0.00415	0.00232	0.00128	0.017	0.00162
GLRLM_LRHGE	906	1410	2470	1270	1830	2050	1040	1600
GLRLM_GLNUr	694	223	4950	2250	1060	1800	2810	114
GLRLM_RLNU	8220	4010	91400	21600	13700	28200	49100	1800
GLRLM_RP	0.902	0.941	0.893	0.821	0.847	0.891	0.797	0.926
NGLDM_Coarseness	0.000714	0.00155	7.53E-05	0.000251	0.000503	0.000229	0	0.00341
NGLDM_Contrast	0.0362	0.0786	0.0596	0.0212	0.0239	0.0433	0	0.0848
NGLDM_Busyness	0.793	0.246	3.63	2	0.716	1.35	0	0.155
GLZLM_SZE	0.658	0.686	0.575	0.526	0.533	0.571	0.578	0.692
GLZLM_LZE	788	28	571	16000	2530	449	11500	75.7
GLZLM_LGZE	0.00257	0.00207	0.00101	0.00329	0.00262	0.0012	0.00323	0.00228
GLZLM_HGZE	759	1280	1580	607	873	1310	592	1330
GLZLM_SZLGE	0.00202	0.00177	0.000644	0.00213	0.002	0.000822	0.00186	0.00202
GLZLM_SZHGE	510	909	889	315	453	722	334	914
GLZLM_LZLGE	1.34	0.0289	0.376	28.2	2.46	0.332	121	0.0637
GLZLM_LZHGE	473000	29300	891000	9190000	2680000	625000	1910000	93300
GLZLM_GLNUz	126	78.3	998	156	115	304	462	31.5
GLZLM_ZLNU	1090	903	8720	921	699	2470	4060	360
GLZLM_ZP	0.246	0.435	0.229	0.1	0.126	0.212	0.156	0.363

Patients with craniopharyngioma	1	2	3	4	5	6	7	8	9	10
minVaule	249	1330	94	130	68	104	193	869	518	131
meanValue	567	4660	396	340	275	289	573	1730	4140	773
stdValue	144	1640	140	117	41.4	60.2	89.2	597	939	164
maxValue	1170	10000	824	856	503	612	832	6380	7490	1200
HISTO_Skewness	1.31	0.502	0.0645	1.22	-0.953	0.544	-0.672	4.3	0.121	-1.04
HISTO_Kurtosis	4.42	2.7	2.26	4.04	5.93	3.93	3.55	26.2	3.17	4.6
HISTO_Entropy_log10	1.5	1.67	1.69	1.52	1.33	1.48	1.53	1.14	1.53	1.56
HISTO_Energy	0.0446	0.0239	0.0222	0.0399	0.0646	0.0388	0.0385	0.107	0.0376	0.0347
SHAPE_Volume	1.54	13.4	1.71	2.04	2.22	7.25	16.5	15.8	24.5	8.04
GLCM_Homogeneity	0.323	0.18	0.259	0.302	0.39	0.407	0.326	0.4	0.262	0.308
GLCM_Energy	0.00472	0.000895	0.00117	0.00373	0.00811	0.00489	0.0043	0.0154	0.0026	0.00256
GLCM_Contrast	81.5	213	117	110	29.6	20.5	78.2	64.1	105	60.5
GLCM_Correlation	0.571	0.293	0.628	0.421	0.48	0.8	0.368	0.227	0.281	0.634
GLCM_Entropy_log10	2.75	3.14	3.1	2.79	2.42	2.57	2.74	2.1	2.91	2.86
GLCM_Dissimilarity	5.96	11.1	7.82	7.01	3.52	3.16	5.93	3.89	7.35	5.37
GLRLM_SRE	0.933	0.979	0.956	0.938	0.914	0.894	0.931	0.921	0.955	0.94
GLRLM_LRE	1.44	1.09	1.27	1.42	1.56	2.02	1.54	1.41	1.22	1.37
GLRLM_LGRE	0.00347	0.00561	0.00499	0.00598	0.00159	0.00264	0.00111	0.0155	0.00145	0.00156
GLRLM_HGRE	644	782	890	490	989	627	1550	166	1220	1610
GLRLM_SRLGE	0.00322	0.00549	0.00455	0.00552	0.00149	0.00238	0.00106	0.0143	0.0014	0.00147
GLRLM_SRHGE	616	769	856	473	900	562	1430	158	1170	1510
GLRLM_LRLGE	0.0051	0.00618	0.00782	0.00911	0.00222	0.00508	0.00147	0.0216	0.0017	0.00218
GLRLM_LRHGE	808	835	1080	596	1570	1240	2490	203	1460	2230
GLRLM_GLNUr	360	81.9	223	448	746	1050	401	473	260	1550
GLRLM_RLNU	7420	3260	8990	10300	9940	22300	9550	3830	6450	39600
GLRLM_RP	0.903	0.972	0.936	0.91	0.881	0.851	0.898	0.897	0.939	0.915
NGLDM_Coarseness	0.000741	0.0018	0.000827	0.000465	0.000665	0.000428	0.000603	0.00109	0.000936	0.000185
NGLDM_Contrast	0.202	0.531	0.387	0.275	0.0466	0.0609	0.177	0.0659	0.208	0.18
NGLDM_Busyness	0.86	0.428	0.771	1.92	0.512	1.3	0.51	0.975	0.472	1.69
GLZLM_SZE	0.692	0.793	0.651	0.685	0.612	0.554	0.66	0.644	0.736	0.622
GLZLM_LZE	169	3.13	23.8	122	1130	1640	342	437	92.5	255
GLZLM_LGZE	0.00317	0.00572	0.00298	0.00455	0.00241	0.00312	0.00167	0.0161	0.00175	0.00158
GLZLM_HGZE	885	848	970	720	922	728	1380	320	1280	1510
GLZLM_SZLGE	0.00198	0.00466	0.00172	0.00261	0.0015	0.00178	0.00132	0.0104	0.00146	0.000945
GLZLM_SZHGE	650	707	645	537	551	426	873	248	945	907
GLZLM_LZLGE	0.627	0.0198	0.543	0.954	1.06	3.08	0.196	6.3	0.106	0.18
GLZLM_LZHGE	49200	2000	10300	19300	1220000	1070000	609000	31900	83200	453000
GLZLM_GLNUz	90.5	56.2	111	123	108	131	106	67.7	113	461
GLZLM_ZLNU	1530	1430	1800	2030	1110	1250	1640	512	1960	5960
GLZLM_ZP	0.357	0.693	0.43	0.358	0.23	0.135	0.328	0.256	0.508	0.331

Patients with craniopharyngioma	11	12	13	14	15	16	17	18	19	20
minVaule	50	0	187	931	496	179	217	125	104	138
meanValue	214	214	367	2090	2450	742	439	393	583	285
stdValue	93.7	98.7	88.3	829	895	96.8	127	216	93	42.4
maxValue	622	782	840	6020	6490	1090	959	1210	1130	614
HISTO_Skewness	0.955	1.73	1.39	1.81	0.374	-0.838	0.665	1.04	-0.0072	1.29
HISTO_Kurtosis	3.18	5.48	4.83	5.93	3.19	5.14	2.91	2.72	4.38	8.56
HISTO_Entropy_log10	1.52	1.25	1.45	1.41	1.57	1.42	1.59	1.43	1.37	1.33
HISTO_Energy	0.0413	0.105	0.0459	0.062	0.0304	0.0501	0.0302	0.0703	0.0529	0.0579
SHAPE_Volume	6.53	8.26	1.63	12.5	0.8	4.36	5.21	11	16.3	2.71
GLCM_Homogeneity	0.349	0.483	0.318	0.322	0.183	0.373	0.279	0.451	0.42	0.341
GLCM_Energy	0.00641	0.0294	0.00437	0.0074	0.0019	0.00523	0.00255	0.0214	0.00671	0.00557
GLCM_Contrast	87.1	37	66.8	105	159	30	98	53.3	19.2	36.3
GLCM_Correlation	0.62	0.712	0.504	0.515	0.164	0.595	0.585	0.844	0.674	0.332
GLCM_Entropy_log10	2.77	2.17	2.68	2.53	2.79	2.56	2.88	2.47	2.45	2.44
GLCM_Dissimilarity	6.05	3.27	5.52	6.16	9.8	3.69	6.87	4.15	2.96	4.19
GLRLM_SRE	0.919	0.863	0.936	0.941	0.982	0.918	0.956	0.873	0.889	0.935
GLRLM_LRE	1.79	2.2	1.41	1.29	1.08	1.52	1.24	2.65	1.9	1.37
GLRLM_LGRE	0.0062	0.00627	0.00528	0.0113	0.00985	0.000782	0.00829	0.0124	0.00131	0.00405
GLRLM_HGRE	505	430	419	350	552	1650	521	494	957	452
GLRLM_SRLGE	0.00545	0.00544	0.00487	0.0106	0.00965	0.000731	0.00776	0.00987	0.00118	0.00383
GLRLM_SRHGE	483	395	402	339	545	1510	508	461	849	426
GLRLM_LRLGE	0.0142	0.0134	0.00795	0.0149	0.0107	0.0011	0.0114	0.0508	0.00232	0.00529
GLRLM_LRHGE	661	709	519	399	585	2570	585	738	1850	596
GLRLM_GLNUr	848	985	409	236	25.1	1170	131	2590	3000	184
GLRLM_RLNU	19600	8710	8020	3530	792	19900	4030	40700	45600	2780
GLRLM_RP	0.872	0.8	0.908	0.921	0.976	0.886	0.938	0.791	0.846	0.912
NGLDM_Coarseness	0.000282	0.000598	0.000675	0.00119	0.0076	0.000342	0.00184	0.000115	0.000155	0.0021
NGLDM_Contrast	0.219	0.0858	0.184	0.281	0.434	0.0659	0.311	0.24	0.0356	0.065
NGLDM_Busyness	3.11	1.17	1.44	0.996	0.129	0.891	0.548	8.72	2.26	0.332
GLZLM_SZE	0.646	0.676	0.66	0.719	0.802	0.606	0.726	0.616	0.563	0.627
GLZLM_LZE	1010	5360	187	193	2.59	964	32.5	18600	5630	60.3
GLZLM_LGZE	0.00363	0.00569	0.00398	0.0102	0.00949	0.00115	0.00655	0.00534	0.00173	0.00518
GLZLM_HGZE	710	680	605	533	593	1460	654	670	945	530
GLZLM_SZLGE	0.00198	0.00302	0.00217	0.00699	0.00696	0.00083	0.0047	0.00266	0.00106	0.00337
GLZLM_SZHGE	482	478	438	403	505	835	508	417	535	349
GLZLM_LZLGE	11.1	27	1.37	2.3	0.025	0.522	0.519	485	5.53	0.209
GLZLM_LZHGE	98900	1080000	29200	18000	1280	1800000	4040	820000	5910000	20300
GLZLM_GLNUz	240	96.8	108	50	17.2	200	63	354	342	48
GLZLM_ZLNU	3290	1420	1400	812	368	2110	1180	4820	2930	404
GLZLM_ZP	0.314	0.222	0.337	0.382	0.724	0.226	0.505	0.198	0.143	0.312

Patients with craniopharyngioma	21	22	23	24	25	26	27	28	29	30
minVaule	48	93	848	207	62	159	53	250	234	102
meanValue	238	495	1490	323	442	597	193	871	654	250
stdValue	61.8	360	428	72.3	91.6	43.3	86.5	373	92.6	144
maxValue	861	1500	5980	851	943	891	617	2360	1590	829
HISTO_Skewness	4.2	0.841	4.91	2.73	-0.308	-1.67	1.72	0.65	1.44	1.97
HISTO_Kurtosis	27.1	2.09	34.3	12.1	3.47	17.3	5.65	2.47	12.7	6.02
HISTO_Entropy_log10	1.02	1.43	1	1.23	1.39	1.05	1.32	1.58	1.22	1.32
HISTO_Energy	0.147	0.0726	0.146	0.0905	0.0517	0.132	0.0888	0.0331	0.0754	0.0967
SHAPE_Volume	12.8	19.7	21.1	14	11.9	15.1	14.3	3.44	9.97	5.35
GLCM_Homogeneity	0.523	0.405	0.477	0.49	0.415	0.505	0.425	0.254	0.45	0.462
GLCM_Energy	0.0357	0.0148	0.029	0.0193	0.00713	0.0293	0.0213	0.00257	0.0104	0.0265
GLCM_Contrast	19.8	48.8	13.8	29.7	30.5	10.7	78.1	109	11.5	62.1
GLCM_Correlation	0.492	0.912	0.34	0.615	0.633	0.401	0.638	0.546	0.632	0.804
GLCM_Entropy_log10	1.83	2.54	1.79	2.14	2.5	1.88	2.37	2.91	2.19	2.3
GLCM_Dissimilarity	2.24	4.16	2.18	2.81	3.51	2.02	4.84	7.49	2.29	4.19
GLRLM_SRE	0.836	0.897	0.894	0.851	0.896	0.845	0.894	0.96	0.883	0.862
GLRLM_LRE	2.56	1.64	1.62	2.4	1.87	2.37	1.97	1.19	1.72	2.41
GLRLM_LGRE	0.00517	0.0153	0.0217	0.0116	0.00169	0.000747	0.00678	0.0102	0.0029	0.0175
GLRLM_HGRE	278	685	109	224	838	1520	405	515	437	409
GLRLM_SRLGE	0.00433	0.013	0.0195	0.00969	0.00152	0.000643	0.00584	0.00977	0.00257	0.0142
GLRLM_SRHGE	242	654	102	207	751	1290	385	504	391	388
GLRLM_LRLGE	0.0131	0.0301	0.0344	0.0301	0.00321	0.00166	0.0157	0.0124	0.00496	0.0513
GLRLM_LRHGE	607	829	146	370	1550	3610	552	567	726	552
GLRLM_GLNUr	5330	857	654	3700	2080	8760	751	118	1700	1900
GLRLM_RLNU	28600	11400	3720	33200	33400	52300	8300	3370	17600	19100
GLRLM_RP	0.777	0.853	0.858	0.792	0.849	0.785	0.841	0.945	0.846	0.792
NGLDM_Coarseness	0.000122	0.000592	0.00115	0.00014	0.000205	7.78E-05	0.000551	0.00173	0.00035	0.000199
NGLDM_Contrast	0.0192	0.332	0.0283	0.0539	0.0543	0.0114	0.147	0.412	0.0157	0.233
NGLDM_Busyness	4.92	1.43	1.13	6.76	1.85	3.06	1.52	0.677	1.37	7.18
GLZLM_SZE	0.602	0.708	0.717	0.64	0.599	0.605	0.665	0.764	0.589	0.686
GLZLM_LZE	57200	1820	2740	26000	3400	90000	2740	37.1	7210	12100
GLZLM_LGZE	0.00513	0.00885	0.0226	0.00682	0.00193	0.00116	0.00425	0.01	0.00341	0.00815
GLZLM_HGZE	555	983	259	547	854	1440	716	643	565	752
GLZLM_SZLGE	0.00252	0.00643	0.0153	0.00343	0.00117	0.000822	0.00241	0.00803	0.00232	0.00411
GLZLM_SZHGE	396	667	219	399	521	827	524	512	354	528
GLZLM_LZLGE	285	46.1	51.2	330	4.19	59.5	26.1	0.537	19.8	293
GLZLM_LZHGE	11600000	88300	154000	2210000	3270000	1.36E+08	292000	4300	2760000	529000
GLZLM_GLNUz	224	103	64.3	251	412	405	88.5	52.6	185	174
GLZLM_ZLNU	1850	2290	518	3180	3650	3440	1340	1130	1220	3100
GLZLM_ZP	0.104	0.284	0.193	0.143	0.217	0.106	0.26	0.529	0.136	0.214

Patients with craniopharyngioma	31	32	33	34	35	36	37	38	39	40
minVaule	147	259	157	355	577	39	5	102	1940	191
meanValue	532	993	410	589	1650	779	784	637	2620	524
stdValue	180	189	46.6	139	747	242	149	178	402	63.2
maxValue	1260	1430	932	1920	6280	1480	1600	1400	5990	1130
HISTO_Skewness	0.388	-0.9	2.72	2.2	2.62	-0.457	-1.15	0.63	3.84	2.47
HISTO_Kurtosis	2.43	3.41	21.5	10.1	10.6	2.32	5.05	3.36	23.7	13.4
HISTO_Entropy_log10	1.6	1.58	0.945	1.19	1.24	1.61	1.25	1.54	1.18	1.05
HISTO_Energy	0.0279	0.0318	0.183	0.0964	0.0946	0.0284	0.0925	0.0346	0.0936	0.149
SHAPE_Volume	13.5	2.93	25.9	19.2	19	12.5	8.2	27.2	4.59	50
GLCM_Homogeneity	0.273	0.278	0.58	0.439	0.382	0.302	0.513	0.332	0.374	0.523
GLCM_Energy	0.00172	0.00215	0.0572	0.022	0.0149	0.00226	0.0285	0.00277	0.0131	0.0413
GLCM_Contrast	97.1	87	10.2	40.8	66.7	87	22	52.2	33.6	18.5
GLCM_Correlation	0.555	0.526	0.422	0.29	0.516	0.608	0.659	0.671	0.453	0.405
GLCM_Entropy_log10	3	2.92	1.61	2.12	2.26	2.96	2.18	2.85	2.08	1.87
GLCM_Dissimilarity	7.03	6.49	1.62	3.73	4.56	6.39	2.78	5.03	3.49	2.37
GLRLM_SRE	0.949	0.951	0.813	0.882	0.924	0.937	0.843	0.928	0.948	0.841
GLRLM_LRE	1.33	1.28	2.86	2.11	1.42	1.41	3.79	1.59	1.24	2.5
GLRLM_LGRE	0.00429	0.00146	0.00268	0.0176	0.0119	0.00174	0.00161	0.00202	0.015	0.00205
GLRLM_HGRE	637	1740	483	149	242	1210	992	818	172	581
GLRLM_SRLGE	0.00397	0.00142	0.00224	0.015	0.0109	0.00165	0.00144	0.00186	0.0143	0.00172
GLRLM_SRHGE	612	1640	400	140	233	1120	813	766	166	501
GLRLM_LRLGE	0.00682	0.00179	0.00699	0.0426	0.0173	0.00224	0.00414	0.00342	0.0179	0.00518
GLRLM_LRHGE	774	2290	1310	221	288	1830	4290	1190	195	1320
GLRLM_GLNUr	1460	532	2730	1160	458	747	2050	3440	98.5	4120
GLRLM_RLNU	46700	15200	11500	11000	4350	23500	21200	85900	964	23400
GLRLM_RP	0.925	0.931	0.748	0.831	0.898	0.909	0.742	0.891	0.932	0.773
NGLDM_Coarseness	0.00014	0.000454	0.000335	0.000321	0.00103	0.000286	0.000211	8.34E-05	0.00549	0.000149
NGLDM_Contrast	0.264	0.249	0.0101	0.0588	0.108	0.276	0.0373	0.128	0.0737	0.0169
NGLDM_Busyness	5.13	0.684	1.3	4.12	1	1.4	1.47	6.07	0.248	2.67
GLZLM_SZE	0.653	0.66	0.625	0.616	0.68	0.687	0.607	0.577	0.73	0.633
GLZLM_LZE	112	35	33000	4510	658	264	16300	651	40.2	29200
GLZLM_LGZE	0.00334	0.00175	0.00407	0.0117	0.0101	0.00191	0.00254	0.00186	0.0177	0.00208
GLZLM_HGZE	727	1500	648	276	449	1080	783	920	253	764
GLZLM_SZLGE	0.00209	0.00118	0.00288	0.00611	0.00603	0.00124	0.00154	0.00103	0.0141	0.00139
GLZLM_SZHGE	488	924	445	194	359	740	461	541	218	509
GLZLM_LZLGE	1.4	0.0231	74.7	110	8.08	0.173	13.6	1.79	0.475	61.5
GLZLM_LZHGE	14800	75600	14600000	191000	56000	440000	19600000	262000	3810	13900000
GLZLM_GLNUz	660	190	103	167	65.8	296	270	942	27.6	331
GLZLM_ZLNU	9330	3010	928	1270	745	5130	2190	9650	250	2720
GLZLM_ZP	0.413	0.405	0.109	0.21	0.299	0.391	0.156	0.271	0.428	0.165

Patients with craniopharyngioma	41	42	43	44	45	46	47	48	49	50
minVaule	1450	134	197	47	17	14	154	157	20	116
meanValue	4890	275	442	266	527	477	730	323	691	436
stdValue	1170	91	126	90.6	65.8	214	250	43.2	81.9	138
maxValue	7380	991	1110	812	1150	1330	1260	684	1250	901
HISTO_Skewness	-0.326	2.66	1.49	1.44	0.0749	0.344	-0.675	0.865	-0.412	0.764
HISTO_Kurtosis	2.54	11.7	5.11	4.7	12.8	2.51	2.01	8.28	10.1	2.78
HISTO_Entropy_log10	1.69	1.21	1.42	1.24	1.04	1.6	1.6	1.24	1.15	1.6
HISTO_Energy	0.0227	0.0954	0.0527	0.101	0.148	0.0277	0.0318	0.0913	0.107	0.0298
SHAPE_Volume	3.21	7.64	1.46	21.2	19.8	1.57	27.2	9.72	4.29	1.41
GLCM_Homogeneity	0.184	0.396	0.374	0.496	0.655	0.219	0.323	0.442	0.523	0.301
GLCM_Energy	0.00151	0.0144	0.00847	0.033	0.0594	0.00141	0.00311	0.0208	0.0255	0.00231
GLCM_Contrast	221	33.2	50.7	38.6	6.37	109	144	23.5	11.6	91.1
GLCM_Correlation	0.265	0.533	0.648	0.572	0.706	0.502	0.68	0.42	0.549	0.664
GLCM_Entropy_log10	2.89	2.22	2.53	2.1	1.68	2.95	2.92	2.14	1.99	2.96
GLCM_Dissimilarity	11.3	3.51	4.58	3.52	1.25	8	7.5	3.08	2.01	6.54
GLRLM_SRE	0.979	0.913	0.913	0.84	0.734	0.971	0.926	0.888	0.834	0.94
GLRLM_LRE	1.09	1.46	1.71	3.24	7.05	1.12	1.61	2.02	2.68	1.38
GLRLM_LGRE	0.00275	0.0155	0.00557	0.00367	0.00138	0.00508	0.00294	0.00359	0.00102	0.0024
GLRLM_HGRE	1570	179	423	471	867	641	1380	466	1280	861
GLRLM_SRLGE	0.00273	0.0142	0.00491	0.0029	0.00107	0.0049	0.00262	0.00326	0.000884	0.00222
GLRLM_SRHGE	1530	171	402	427	630	627	1280	420	1070	825
GLRLM_LRLGE	0.00283	0.0227	0.011	0.0149	0.00841	0.00584	0.00608	0.00648	0.00241	0.00362
GLRLM_LRHGE	1740	218	567	998	6170	701	2100	881	3410	1060
GLRLM_GLNUr	22.8	597	372	3670	6910	42.7	3030	566	1900	229
GLRLM_RLNU	951	5490	6460	35000	33700	1430	84200	5670	14100	6900
GLRLM_RP	0.971	0.881	0.87	0.756	0.611	0.961	0.887	0.838	0.769	0.913
NGLDM_Coarseness	0.00628	0.000855	0.000864	8.18E-05	0.000109	0.00482	5.69E-05	0.000868	0.000347	0.00106
NGLDM_Contrast	0.624	0.0704	0.145	0.083	0.00673	0.39	0.571	0.0385	0.0142	0.278
NGLDM_Busyness	0.0757	1.36	1.11	7.1	2.76	0.206	6.3	0.626	0.819	0.721
GLZLM_SZE	0.787	0.769	0.62	0.617	0.647	0.739	0.662	0.648	0.642	0.65
GLZLM_LZE	3.1	1850	669	32700	168000	5.67	1360	2240	17800	113
GLZLM_LGZE	0.0035	0.0154	0.00388	0.00227	0.00223	0.00501	0.00239	0.00481	0.00149	0.00201
GLZLM_HGZE	1500	358	631	747	710	702	1190	546	1270	1050
GLZLM_SZLGE	0.00328	0.012	0.002	0.00127	0.00147	0.00397	0.00148	0.00317	0.00088	0.0014
GLZLM_SZHGE	1140	303	432	480	445	541	728	363	790	721
GLZLM_LZLGE	0.00524	29	5.87	173	197	0.044	12.2	5.84	14	0.436
GLZLM_LZHGE	5360	124000	81300	6240000	1.44E+08	2430	736000	865000	22700000	36000
GLZLM_GLNUz	15.4	82.8	83.1	402	407	26.2	773	74.1	117	81.3
GLZLM_ZLNU	408	1150	934	3910	3100	489	13700	735	1240	1320
GLZLM_ZP	0.687	0.267	0.286	0.167	0.0779	0.6	0.299	0.218	0.121	0.396

Patients with craniopharyngioma	51	52	53	54	55	56	57	58	59	60
minVaule	9	91	11	912	64	1400	139	88	79	34
meanValue	534	370	717	5520	437	3660	347	617	317	252
stdValue	140	130	418	982	77.5	388	105	194	85.3	76
maxValue	1750	926	2170	7890	841	4770	1090	1090	761	894
HISTO_Skewness	2.62	0.84	0.0448	-0.693	-0.415	-1.2	1.2	-0.794	0.769	2.2
HISTO_Kurtosis	14.6	3.21	1.65	3.7	4.4	6.84	4.64	2.67	3.42	8.96
HISTO_Entropy_log10	1.12	1.45	1.58	1.51	1.4	1.42	1.38	1.62	1.49	1.14
HISTO_Energy	0.122	0.0651	0.0311	0.0398	0.0487	0.0527	0.0531	0.0282	0.0384	0.124
SHAPE_Volume	16.5	10.6	14.4	8.77	6.08	10.8	4.84	11.3	11.1	21.1
GLCM_Homogeneity	0.456	0.413	0.294	0.216	0.396	0.302	0.43	0.305	0.312	0.528
GLCM_Energy	0.0224	0.0221	0.00311	0.00475	0.00542	0.00515	0.00823	0.00197	0.00273	0.0351
GLCM_Contrast	23.3	78.3	76.9	114	21.2	47.9	27.1	73.9	59.8	18.2
GLCM_Correlation	0.506	0.59	0.756	0.167	0.691	0.307	0.681	0.759	0.549	0.615
GLCM_Entropy_log10	2.06	2.5	2.94	2.58	2.51	2.52	2.44	2.96	2.81	1.95
GLCM_Dissimilarity	2.73	5.4	6.2	8.51	3.22	4.93	3.26	5.81	5.41	2.34
GLRLM_SRE	0.882	0.899	0.942	0.961	0.904	0.957	0.888	0.939	0.937	0.824
GLRLM_LRE	1.68	2.02	1.31	1.23	1.7	1.2	1.92	1.37	1.39	2.95
GLRLM_LGRE	0.00316	0.00452	0.0106	0.00125	0.00134	0.00145	0.00876	0.00244	0.00277	0.00451
GLRLM_HGRE	428	628	633	1900	1020	1930	278	1340	598	343
GLRLM_SRLGE	0.00281	0.00406	0.0095	0.00123	0.00123	0.00143	0.00754	0.00222	0.00257	0.00364
GLRLM_SRHGE	385	590	608	1820	918	1840	257	1250	568	301
GLRLM_LRLGE	0.00512	0.0096	0.0173	0.00139	0.00213	0.00156	0.019	0.00401	0.00408	0.0142
GLRLM_LRHGE	677	952	745	2370	1720	2320	431	1810	766	783
GLRLM_GLNUr	1630	379	426	103	1550	130	1230	1800	2040	9750
GLRLM_RLNU	10900	6370	12800	2430	26100	2300	19500	55400	46500	66300
GLRLM_RP	0.84	0.842	0.917	0.946	0.866	0.943	0.837	0.913	0.91	0.748
NGLDM_Coarseness	0.000443	0.00102	0.000599	0.00175	0.000287	0.00289	0.000345	0.000146	0.000138	5.41E-05
NGLDM_Contrast	0.0291	0.202	0.402	0.255	0.0437	0.119	0.0785	0.3	0.118	0.0303
NGLDM_Busyness	1.22	0.669	1.48	0.186	1.19	0.115	3.14	2.54	4.24	11.3
GLZLM_SZE	0.718	0.685	0.714	0.682	0.592	0.738	0.623	0.642	0.626	0.58
GLZLM_LZE	8920	1290	288	12.4	2200	27.9	3950	141	375	111000
GLZLM_LGZE	0.00366	0.00333	0.00748	0.00186	0.00192	0.0023	0.00547	0.00217	0.00244	0.00313
GLZLM_HGZE	610	854	695	1810	966	1870	464	1200	722	606
GLZLM_SZLGE	0.00271	0.00212	0.00562	0.00162	0.00128	0.00215	0.00267	0.00139	0.00153	0.00154
GLZLM_SZHGE	471	609	486	1200	554	1360	312	728	472	380
GLZLM_LZLGE	24.7	4.97	8.78	0.00807	2.2	0.016	51	0.854	1.55	549
GLZLM_LZHGE	3250000	357000	17500	25500	2320000	55300	368000	174000	96300	22800000
GLZLM_GLNUz	140	80.4	182	40.6	206	45.8	201	575	679	623
GLZLM_ZLNU	1590	1350	3390	575	2070	670	2000	9150	7380	4950
GLZLM_ZP	0.189	0.325	0.443	0.481	0.168	0.489	0.183	0.339	0.339	0.119

Patients with craniopharyngioma	61	62	63
minVaule	152	60	44
meanValue	424	287	449
stdValue	165	104	84.5
maxValue	1230	807	777
HISTO_Skewness	1.33	1.46	-1.39
HISTO_Kurtosis	4.35	5.16	4.87
HISTO_Entropy_log10	1.4	1.4	1.33
HISTO_Energy	0.0655	0.0653	0.0733
SHAPE_Volume	9.07	6.49	16.3
GLCM_Homogeneity	0.421	0.348	0.469
GLCM_Energy	0.0165	0.01	0.0152
GLCM_Contrast	68.9	65.7	22.3
GLCM_Correlation	0.601	0.593	0.768
GLCM_Entropy_log10	2.46	2.59	2.33
GLCM_Dissimilarity	4.87	5.18	2.81
GLRLM_SRE	0.885	0.927	0.867
GLRLM_LRE	2.22	1.4	2.16
GLRLM_LGRE	0.00681	0.0047	0.00128
GLRLM_HGRE	422	500	1310
GLRLM_SRLGE	0.00561	0.00438	0.00116
GLRLM_SRHGE	400	478	1110
GLRLM_LRLGE	0.0204	0.00648	0.00218
GLRLM_LRHGE	604	614	3050
GLRLM_GLNUr	1540	340	5090
GLRLM_RLNU	23800	4980	59500
GLRLM_RP	0.82	0.897	0.807
NGLDM_Coarseness	0.000172	0.0012	0.000109
NGLDM_Contrast	0.176	0.137	0.0515
NGLDM_Busyness	5.17	0.544	2.5
GLZLM_SZE	0.645	0.757	0.623
GLZLM_LZE	6360	663	25700
GLZLM_LGZE	0.0036	0.00535	0.00195
GLZLM_HGZE	668	680	1070
GLZLM_SZLGE	0.00202	0.0044	0.0012
GLZLM_SZHGE	457	532	655
GLZLM_LZLGE	77	3.02	16.6
GLZLM_LZHGE	541000	148000	40000000
GLZLM_GLNUz	278	75.4	559
GLZLM_ZLNU	3620	1380	6690
GLZLM_ZP	0.249	0.386	0.183

Patients with meningioma	1	2	3	4	5	6	7	8	9	10
minVaule	141	171	137	5	276	33	369	181	94	29
meanValue	646	361	367	421	652	165	722	872	573	547
stdValue	65.4	36.1	53.7	61.7	75.3	36.2	72.2	169	118	99.4
maxValue	1270	645	583	855	1070	349	1080	1330	865	934
HISTO_Skewness	-0.31	0.0961	-0.374	-0.143	0.0405	0.648	-0.51	-0.0712	-0.666	-1.14
HISTO_Kurtosis	11.8	6.94	3.63	6.06	5.42	3.65	6.52	3.02	3.07	6.45
HISTO_Entropy_log10	1.12	1.27	1.49	1.26	1.37	1.46	1.38	1.4	1.58	1.38
HISTO_Energy	0.104	0.0686	0.0392	0.0734	0.056	0.0415	0.056	0.0463	0.0307	0.0579
SHAPE_Volume	12.9	1.1	1.5	4.8	0.877	12	1.5	5.51	1.74	4.92
GLCM_Homogeneity	0.519	0.408	0.331	0.443	0.341	0.306	0.418	0.354	0.27	0.325
GLCM_Energy	0.0214	0.00846	0.00339	0.0103	0.00589	0.00285	0.00706	0.00376	0.00192	0.00476
GLCM_Contrast	9.63	17.3	42.5	13.2	33.8	45.4	28.5	21.9	90.1	41.2
GLCM_Correlation	0.562	0.556	0.564	0.635	0.376	0.566	0.62	0.681	0.445	0.58
GLCM_Entropy_log10	1.97	2.29	2.69	2.26	2.47	2.76	2.44	2.57	2.91	2.59
GLCM_Dissimilarity	1.91	2.9	4.57	2.51	4.14	4.92	3.24	3.47	6.65	4.42
GLRLM_SRE	0.836	0.908	0.935	0.887	0.935	0.943	0.904	0.937	0.957	0.939
GLRLM_LRE	2.61	1.6	1.39	1.83	1.39	1.32	1.65	1.3	1.24	1.29
GLRLM_LGRE	0.00137	0.00229	0.00138	0.00118	0.0015	0.00174	0.00253	0.00163	0.00108	0.00115
GLRLM_HGRE	862	709	1170	1030	982	796	1080	817	1710	1430
GLRLM_SRLGE	0.00118	0.00214	0.00132	0.00106	0.00143	0.00165	0.00233	0.00154	0.00104	0.0011
GLRLM_SRHGE	715	644	1090	909	921	753	975	765	1620	1350
GLRLM_LRLGE	0.00322	0.00326	0.00176	0.00201	0.00195	0.00228	0.00394	0.00204	0.00126	0.00142
GLRLM_LRHGE	2300	1130	1660	1910	1360	1030	1790	1060	2160	1830
GLRLM_GLNUr	4420	395	327	1810	274	1930	437	236	309	259
GLRLM_RLNU	31900	4800	7280	19700	4340	41000	6450	4390	9170	3940
GLRLM_RP	0.777	0.873	0.91	0.843	0.91	0.921	0.868	0.916	0.939	0.919
NGLDM_Coarseness	0.000154	0.00137	0.00108	0.000307	0.00138	0.000163	0.0011	0.00188	0.000737	0.00168
NGLDM_Contrast	0.00941	0.0343	0.0928	0.0227	0.0676	0.0941	0.0535	0.102	0.234	0.0857
NGLDM_Busyness	2	0.372	0.329	1.19	0.33	2.95	0.321	0.434	0.461	0.217
GLZLM_SZE	0.604	0.622	0.592	0.588	0.645	0.596	0.597	0.665	0.664	0.709
GLZLM_LZE	46900	501	80.2	5570	105	45.7	766	64.5	20.8	112
GLZLM_LGZE	0.00253	0.00405	0.00215	0.00167	0.00216	0.00183	0.00362	0.0021	0.00129	0.0016
GLZLM_HGZE	750	717	1060	941	1010	851	1020	766	1530	1500
GLZLM_SZLGE	0.0019	0.00275	0.00164	0.000998	0.00171	0.00112	0.00207	0.00159	0.000825	0.00133
GLZLM_SZHGE	439	449	598	534	644	519	594	491	969	1070
GLZLM_LZLGE	52.5	0.715	0.068	5.56	0.119	0.0778	0.818	0.0908	0.0128	0.0873
GLZLM_LZHGE	42200000	366000	105000	5640000	96200	31400	782000	51800	42200	153000
GLZLM_GLNUz	261	53.2	83.8	192	63.4	583	56.5	69.9	128	63.4
GLZLM_ZLNU	1850	482	891	1430	667	5630	595	750	1950	782
GLZLM_ZP	0.0924	0.196	0.291	0.145	0.311	0.33	0.192	0.326	0.44	0.334

Patients with meningioma	11	12	13	14	15	16	17	18	19	20
minVaule	169	167	204	88	25	219	141	179	41	1550
meanValue	498	601	796	723	671	711	563	354	226	2560
stdValue	45.6	97.2	87.8	119	70.4	49.4	76.9	43.7	27.9	316
maxValue	840	1390	1220	1520	1260	999	1060	643	387	3310
HISTO_Skewness	0.0733	0.66	-0.0956	-0.275	-0.18	-0.837	0.0907	-0.457	0.152	-0.968
HISTO_Kurtosis	4.44	6.67	6.27	5.5	11.2	8.46	6.2	4.14	6.49	3.58
HISTO_Entropy_log10	1.25	1.28	1.33	1.32	1.11	1.18	1.32	1.35	1.29	1.6
HISTO_Energy	0.0682	0.0706	0.0617	0.0617	0.111	0.0868	0.0613	0.0552	0.067	0.0307
SHAPE_Volume	8.37	5.95	4.96	20.4	5.55	7.87	4.08	3.9	4.52	2.76
GLCM_Homogeneity	0.424	0.422	0.414	0.364	0.524	0.447	0.379	0.302	0.461	0.209
GLCM_Energy	0.00833	0.00962	0.00775	0.00559	0.0236	0.0129	0.00647	0.00487	0.0102	0.00262
GLCM_Contrast	13.1	16.4	16.4	23.5	8.8	13.4	23.3	52	13	174
GLCM_Correlation	0.609	0.647	0.665	0.552	0.582	0.487	0.568	0.144	0.704	0.274
GLCM_Entropy_log10	2.28	2.32	2.36	2.48	1.94	2.15	2.43	2.5	2.27	2.65
GLCM_Dissimilarity	2.61	2.81	2.84	3.41	1.86	2.48	3.37	5.22	2.38	9.66
GLRLM_SRE	0.894	0.895	0.899	0.924	0.833	0.88	0.92	0.95	0.873	0.976
GLRLM_LRE	1.71	1.78	1.69	1.37	2.59	1.87	1.47	1.25	1.97	1.11
GLRLM_LGRE	0.00108	0.00233	0.000828	0.00147	0.00101	0.000681	0.00148	0.00282	0.000998	0.00367
GLRLM_HGRE	1040	564	1460	865	1170	1680	934	641	1230	1520
GLRLM_SRLGE	0.000967	0.00211	0.000756	0.00138	0.000867	0.000607	0.00138	0.00271	0.000889	0.00364
GLRLM_SRHGE	933	507	1310	799	972	1470	861	606	1080	1480
GLRLM_LRLGE	0.00182	0.00402	0.00131	0.00195	0.00238	0.00121	0.00204	0.00334	0.00183	0.00379
GLRLM_LRHGE	1750	980	2490	1180	3060	3170	1370	818	2430	1710
GLRLM_GLNUr	2990	1700	1610	1150	2690	3400	302	189	1490	21.2
GLRLM_RLNU	34800	19900	21300	15700	18400	31000	4200	3130	17400	657
GLRLM_RP	0.855	0.855	0.861	0.899	0.773	0.836	0.89	0.933	0.826	0.968
NGLDM_Coarseness	0.000176	0.000314	0.000309	0.000438	0.000246	0.000161	0.00156	0.00167	0.000385	0.00961
NGLDM_Contrast	0.0223	0.0277	0.0306	0.0373	0.0101	0.0175	0.0397	0.112	0.0239	0.472
NGLDM_Busyness	2.19	1.65	0.908	0.974	1.2	1.77	0.266	0.372	0.836	0.0418
GLZLM_SZE	0.546	0.535	0.557	0.682	0.588	0.582	0.611	0.664	0.582	0.771
GLZLM_LZE	7030	4890	3170	1040	33900	18300	285	30.1	7520	3.83
GLZLM_LGZE	0.00134	0.00275	0.00132	0.00192	0.00207	0.000963	0.00218	0.00337	0.00174	0.00482
GLZLM_HGZE	1080	650	1330	855	1050	1550	969	596	1130	1450
GLZLM_SZLGE	0.000853	0.00136	0.000937	0.00143	0.00163	0.000557	0.00166	0.00214	0.00127	0.00444
GLZLM_SZHGE	582	378	692	578	595	860	604	388	609	1080
GLZLM_LZLGE	7.31	9.3	2.22	1.25	28.7	10.6	0.321	0.0465	6.36	0.00758
GLZLM_LZHGE	6860000	2610000	4590000	883000	40200000	31700000	258000	22800	9040000	6340
GLZLM_GLNUz	304	150	173	203	138	254	57.6	66.6	126	12.7
GLZLM_ZLNU	1880	1090	1360	2250	936	1880	497	659	1080	261
GLZLM_ZP	0.127	0.136	0.15	0.247	0.0841	0.121	0.249	0.426	0.12	0.654

Patients with meningioma	21	22	23	24	25	26	27	28	29	30
minVaule	199	25	149	261	188	113	226	1280	335	86
meanValue	410	897	585	537	345	675	551	2780	624	644
stdValue	52.7	111	64.6	47.6	46.7	93.2	55.2	351	80	120
maxValue	838	1430	896	963	729	1320	905	4160	1080	1020
HISTO_Skewness	1.19	-0.385	-0.627	1.02	1.45	-0.394	0.436	0.0478	0.519	-0.647
HISTO_Kurtosis	7.79	5.36	5.33	8.92	7.04	7.34	5	4.91	4.68	4.53
HISTO_Entropy_log10	1.28	1.31	1.34	1.19	1.3	1.27	1.32	1.45	1.44	1.51
HISTO_Energy	0.071	0.059	0.056	0.0892	0.0643	0.068	0.059	0.0508	0.0446	0.0381
SHAPE_Volume	2.26	3.27	21.4	5.64	13.2	6.36	3.9	4.17	2.06	12.7
GLCM_Homogeneity	0.406	0.466	0.396	0.483	0.413	0.417	0.385	0.33	0.314	0.277
GLCM_Energy	0.00923	0.0087	0.00562	0.0161	0.00883	0.00806	0.00628	0.00577	0.00347	0.00206
GLCM_Contrast	21.8	14.1	14.4	10.1	24.7	13.5	20	66.5	44.1	56.3
GLCM_Correlation	0.532	0.694	0.727	0.661	0.495	0.647	0.583	0.21	0.488	0.568
GLCM_Entropy_log10	2.33	2.3	2.44	2.09	2.35	2.29	2.41	2.53	2.67	2.88
GLCM_Dissimilarity	3.13	2.42	2.79	2.12	3.24	2.58	3.23	5.35	4.85	5.47
GLRLM_SRE	0.907	0.869	0.912	0.86	0.895	0.908	0.914	0.941	0.939	0.953
GLRLM_LRE	1.61	2.14	1.44	2.18	1.91	1.47	1.55	1.35	1.36	1.21
GLRLM_LGRE	0.00299	0.000747	0.000894	0.00177	0.00353	0.00157	0.0012	0.0017	0.00232	0.00124
GLRLM_HGRE	502	1650	1460	686	407	939	1000	1210	693	1570
GLRLM_SRLGE	0.00275	0.000663	0.000831	0.00154	0.00314	0.00147	0.0011	0.00163	0.00219	0.0012
GLRLM_SRHGE	459	1430	1330	595	371	851	915	1140	655	1500
GLRLM_LRLGE	0.00457	0.00147	0.00121	0.00373	0.00699	0.00209	0.00181	0.00211	0.00308	0.0014
GLRLM_LRHGE	775	3520	2120	1450	701	1390	1540	1620	923	1910
GLRLM_GLNUr	830	995	1050	2140	2880	376	921	185	503	498
GLRLM_RLNU	9900	13000	15300	18800	37300	4510	13100	3320	9940	11700
GLRLM_RP	0.871	0.82	0.882	0.808	0.85	0.877	0.882	0.919	0.916	0.938
NGLDM_Coarseness	0.000621	0.000539	0.000486	0.000284	0.000146	0.00151	0.000471	0.00206	0.000619	0.00068
NGLDM_Contrast	0.0353	0.0246	0.0333	0.0167	0.0431	0.0268	0.0381	0.114	0.0824	0.116
NGLDM_Busyness	0.89	0.56	0.62	1.52	4.26	0.247	1	0.185	0.742	0.451
GLZLM_SZE	0.604	0.58	0.703	0.554	0.56	0.73	0.573	0.638	0.592	0.664
GLZLM_LZE	1440	4900	2740	19500	6880	1050	758	62.1	52.8	23.4
GLZLM_LGZE	0.00397	0.00142	0.00148	0.00236	0.00318	0.00304	0.0015	0.00222	0.00268	0.00178
GLZLM_HGZE	602	1540	1400	791	584	889	1020	1250	765	1540
GLZLM_SZLGE	0.00227	0.00111	0.00123	0.00128	0.00161	0.00268	0.00101	0.0013	0.00171	0.00146
GLZLM_SZHGE	383	846	975	452	354	644	590	791	467	1000
GLZLM_LZLGE	3.42	3.11	1.87	32.6	26.7	1.07	0.831	0.0608	0.107	0.0172
GLZLM_LZHGE	617000	7910000	4080000	11800000	1820000	1050000	710000	69600	30800	36900
GLZLM_GLNUz	102	90.4	153	125	333	52.9	155	44.7	139	182
GLZLM_ZLNU	950	734	1980	912	2470	668	1120	546	1280	2440
GLZLM_ZP	0.197	0.111	0.199	0.0988	0.151	0.206	0.201	0.349	0.312	0.423

Patients with meningioma	31	32	33	34	35	36	37	38	39	40
minVaule	46	282	201	196	424	166	234	78	497	167
meanValue	915	741	476	694	793	545	667	412	908	520
stdValue	202	96.7	59.6	60	161	39.8	66.5	58.1	163	70.9
maxValue	1740	1300	816	1020	1690	672	995	604	1600	944
HISTO_Skewness	-0.397	-0.126	0.638	-0.605	1.97	-1.71	-1.15	-0.787	0.158	0.257
HISTO_Kurtosis	3.41	4.9	7.37	8.31	10.4	12.5	9.2	4.48	2.96	4.67
HISTO_Entropy_log10	1.49	1.39	1.36	1.25	1.38	1.26	1.31	1.44	1.57	1.37
HISTO_Energy	0.0382	0.0501	0.06	0.0709	0.0625	0.0698	0.0641	0.0436	0.0307	0.0525
SHAPE_Volume	13.5	4.66	2.3	4.1	2.4	2.68	12.9	11.5	4.59	3.74
GLCM_Homogeneity	0.401	0.308	0.389	0.438	0.335	0.417	0.364	0.33	0.303	0.347
GLCM_Energy	0.00371	0.00347	0.00702	0.00961	0.00739	0.00895	0.00624	0.00362	0.00248	0.00501
GLCM_Contrast	18.5	33.4	26	14.8	77.1	21.6	29.7	40.3	106	29.4
GLCM_Correlation	0.817	0.481	0.585	0.596	0.321	0.448	0.388	0.49	0.378	0.496
GLCM_Entropy_log10	2.61	2.61	2.43	2.27	2.42	2.29	2.44	2.65	2.81	2.52
GLCM_Dissimilarity	2.96	4.33	3.41	2.59	5.52	2.93	3.69	4.46	6.8	3.96
GLRLM_SRE	0.914	0.946	0.912	0.888	0.935	0.9	0.925	0.934	0.943	0.928
GLRLM_LRE	1.45	1.24	1.61	1.81	1.51	1.68	1.42	1.38	1.35	1.45
GLRLM_LGRE	0.00135	0.00197	0.00167	0.000805	0.00749	0.000641	0.00117	0.000862	0.0049	0.00142
GLRLM_HGRE	1160	908	886	1560	443	2370	1400	1740	684	910
GLRLM_SRLGE	0.00127	0.00189	0.00155	0.00073	0.00711	0.000599	0.00111	0.000816	0.00471	0.00133
GLRLM_SRHGE	1060	861	808	1380	419	2130	1290	1620	644	844
GLRLM_LRLGE	0.00179	0.0023	0.00245	0.00134	0.0104	0.000929	0.00155	0.00111	0.00612	0.00198
GLRLM_LRHGE	1710	1120	1420	2810	619	4000	1970	2430	907	1310
GLRLM_GLNUr	464	219	733	1560	117	968	997	487	112	1080
GLRLM_RLNU	9830	3850	10300	17600	1700	11400	13300	9750	3250	17700
GLRLM_RP	0.883	0.929	0.878	0.846	0.905	0.862	0.898	0.91	0.92	0.9
NGLDM_Coarseness	0.00112	0.00169	0.000631	0.000358	0.00258	0.000513	0.000449	0.000704	0.0021	0.000332
NGLDM_Contrast	0.0597	0.0778	0.0453	0.0201	0.133	0.0305	0.041	0.0934	0.25	0.0472
NGLDM_Busyness	0.325	0.28	0.569	0.703	0.246	0.448	0.61	0.51	0.307	1.08
GLZLM_SZE	0.712	0.677	0.58	0.587	0.615	0.616	0.624	0.629	0.598	0.567
GLZLM_LZE	477	51.3	650	4870	93.6	1830	218	68	32.7	153
GLZLM_LGZE	0.00227	0.00288	0.0027	0.00149	0.00988	0.00145	0.00162	0.0011	0.00661	0.00186
GLZLM_HGZE	1030	945	894	1510	597	2200	1410	1610	711	923
GLZLM_SZLGE	0.0019	0.00236	0.00199	0.00119	0.0069	0.00126	0.00106	0.000653	0.00428	0.00127
GLZLM_SZHGE	709	649	505	848	432	1290	877	969	436	522
GLZLM_LZLGE	0.433	0.0684	0.803	3.33	0.404	0.789	0.165	0.0386	0.093	0.187
GLZLM_LZHGE	589000	42300	541000	7180000	30200	4290000	300000	129000	21800	132000
GLZLM_GLNUz	102	67.9	99.3	149	22.1	120	187	130	36.2	213
GLZLM_ZLNU	1630	740	878	1200	219	1030	1680	1460	457	1730
GLZLM_ZP	0.25	0.366	0.194	0.138	0.289	0.175	0.261	0.318	0.339	0.246

Patients with meningioma	41	42	43	44	45	46	47	48	49	50
minVaule	315	78	710	51	277	207	439	1510	88	75
meanValue	931	203	5000	197	853	1300	1380	3420	449	640
stdValue	198	46.6	1320	60.5	136	145	132	371	47.4	120
maxValue	2070	643	7230	582	1310	2340	2170	5100	808	1670
HISTO_Skewness	0.442	2.34	-0.751	1.08	-0.422	-1.12	-0.317	-0.569	-0.51	0.602
HISTO_Kurtosis	3.92	13.8	3.36	4.83	3.33	8.58	5.52	6.67	5.66	4.18
HISTO_Entropy_log10	1.46	1.24	1.55	1.43	1.53	1.18	1.29	1.36	1.22	1.28
HISTO_Energy	0.0416	0.0781	0.0359	0.0473	0.0344	0.0906	0.0648	0.0598	0.0755	0.0616
SHAPE_Volume	9.9	0.529	0.465	6.52	11.9	28.1	23.8	3.96	9.74	9.1
GLCM_Homogeneity	0.312	0.399	0.226	0.319	0.282	0.484	0.401	0.299	0.469	0.443
GLCM_Energy	0.00335	0.0107	0.0181	0.00482	0.00217	0.0181	0.00798	0.00581	0.0122	0.00904
GLCM_Contrast	54.9	27.4	195	68.3	72.6	15	22.6	58.5	12.3	13.1
GLCM_Correlation	0.426	0.32	0.24	0.288	0.42	0.495	0.436	0.181	0.594	0.7
GLCM_Entropy_log10	2.69	2.21	1.85	2.56	2.85	2.1	2.35	2.41	2.17	2.28
GLCM_Dissimilarity	5.33	3.31	9.59	5.53	6.04	2.4	3.27	5.27	2.34	2.58
GLRLM_SRE	0.939	0.916	0.983	0.941	0.952	0.845	0.899	0.959	0.862	0.872
GLRLM_LRE	1.38	1.51	1.07	1.38	1.26	3.05	1.75	1.18	2.25	2.21
GLRLM_LGRE	0.00325	0.00703	0.00895	0.00653	0.00131	0.00112	0.001	0.00207	0.00106	0.0022
GLRLM_HGRE	581	252	1980	392	1370	1100	1270	1240	1080	562
GLRLM_SRLGE	0.00308	0.00647	0.00893	0.00608	0.00126	0.000974	0.000917	0.00203	0.000928	0.00192
GLRLM_SRHGE	547	236	1950	375	1310	923	1140	1190	924	493
GLRLM_LRLGE	0.00431	0.0104	0.00901	0.00917	0.00157	0.00297	0.00162	0.00222	0.00225	0.00481
GLRLM_LRHGE	789	349	2130	497	1740	3470	2210	1480	2440	1210
GLRLM_GLNUr	325	217	4.36	204	278	7310	1070	59.3	2410	1900
GLRLM_RLNU	6910	2380	120	3920	7290	64800	13800	922	24700	24400
GLRLM_RP	0.915	0.885	0.978	0.918	0.934	0.786	0.864	0.946	0.815	0.829
NGLDM_Coarseness	0.000934	0.00232	0	0.00157	0.000945	6.72E-05	0.000458	0.00598	0.000219	0.000258
NGLDM_Contrast	0.109	0.0526	0	0.124	0.15	0.0182	0.0349	0.127	0.0187	0.0278
NGLDM_Busyness	0.634	0.42	0	0.569	0.388	4.21	0.836	0.0905	1.64	2.16
GLZLM_SZE	0.588	0.636	0.854	0.647	0.661	0.557	0.566	0.703	0.522	0.451
GLZLM_LZE	48.1	198	2.26	50.4	20.3	73500	957	14.4	18000	6800
GLZLM_LGZE	0.00394	0.00753	0.011	0.00574	0.00155	0.00203	0.00157	0.00327	0.00175	0.00281
GLZLM_HGZE	629	378	1960	522	1350	900	1250	1200	949	616
GLZLM_SZLGE	0.00237	0.00401	0.0109	0.00326	0.001	0.00122	0.00117	0.003	0.00118	0.00145
GLZLM_SZHGE	391	283	1670	377	876	473	699	824	473	290
GLZLM_LZLGE	0.111	1.29	0.0121	0.304	0.0182	62.7	0.794	0.0137	16	14.3
GLZLM_LZHGE	27400	32900	4570	11500	28000	86700000	1170000	18900	20500000	3490000
GLZLM_GLNUz	88.4	34.3	2.84	58.8	112	396	150	20.4	148	129
GLZLM_ZLNU	852	302	68.1	668	1510	2670	1100	236	902	609
GLZLM_ZP	0.305	0.245	0.773	0.355	0.426	0.0834	0.184	0.478	0.0862	0.0808

Patients with meningioma	51	52	53	54	55	56	57	58	59	60
minVaule	220	170	3040	199	143	140	31	343	353	245
meanValue	695	677	5100	727	703	560	513	749	786	730
stdValue	111	69.7	428	83.1	97.6	65	59.2	87.9	64.9	77.6
maxValue	1250	1020	6580	1870	1490	965	946	1220	1140	1130
HISTO_Skewness	0.329	-0.395	-0.431	0.534	-0.0235	0.3	-0.206	0.156	-0.0668	-0.334
HISTO_Kurtosis	3.77	7.16	5.44	15.9	4.6	5.69	6.75	4.21	4.16	5.04
HISTO_Entropy_log10	1.45	1.3	1.46	1.08	1.27	1.3	1.21	1.41	1.33	1.35
HISTO_Energy	0.0427	0.0663	0.042	0.107	0.067	0.0647	0.0786	0.0501	0.056	0.0579
SHAPE_Volume	4.87	4.63	3.15	6.68	19	3.42	6.76	7	11.9	10.3
GLCM_Homogeneity	0.253	0.442	0.241	0.518	0.412	0.421	0.439	0.38	0.414	0.415
GLCM_Energy	0.00226	0.00952	0.0036	0.0204	0.00834	0.00824	0.0108	0.00545	0.00658	0.0073
GLCM_Contrast	68.8	17.4	68.7	7.8	16.8	16.2	12	27	17.2	15.8
GLCM_Correlation	0.254	0.617	0.278	0.552	0.553	0.625	0.579	0.634	0.658	0.698
GLCM_Entropy_log10	2.81	2.3	2.54	1.94	2.32	2.33	2.2	2.54	2.41	2.39
GLCM_Dissimilarity	6.27	2.69	6.42	1.8	2.9	2.8	2.46	3.57	2.87	2.81
GLRLM_SRE	0.962	0.878	0.971	0.841	0.897	0.895	0.887	0.911	0.895	0.897
GLRLM_LRE	1.18	1.96	1.14	2.35	1.73	1.76	1.81	1.61	1.72	1.71
GLRLM_LGRE	0.00158	0.000838	0.00235	0.00265	0.00158	0.00111	0.00097	0.00148	0.000868	0.000916
GLRLM_HGRE	959	1530	1500	440	757	1120	1190	960	1320	1300
GLRLM_SRLGE	0.00151	0.00075	0.00233	0.00227	0.00143	0.001	0.000869	0.00137	0.000781	0.000833
GLRLM_SRHGE	925	1340	1450	369	679	1000	1050	876	1180	1160
GLRLM_LRLGE	0.00189	0.00152	0.00246	0.00588	0.00266	0.00184	0.00169	0.00224	0.00146	0.00148
GLRLM_LRHGE	1120	2990	1700	1040	1310	1960	2150	1530	2260	2250
GLRLM_GLNUr	638	1530	38.3	3310	4450	1160	2700	1480	3510	3120
GLRLM_RLNU	13700	18400	858	22700	53900	14500	27300	24900	50100	43800
GLRLM_RP	0.949	0.835	0.961	0.786	0.859	0.855	0.845	0.877	0.856	0.859
NGLDM_Coarseness	0.000439	0.000325	0.00701	0.00022	0.00011	0.000404	0.000218	0.000273	0.000136	0.000151
NGLDM_Contrast	0.106	0.0274	0.154	0.00729	0.0246	0.0325	0.0166	0.0518	0.0308	0.0326
NGLDM_Busyness	0.978	0.823	0.0662	2.1	3.54	1.06	1.53	1.32	2.3	2
GLZLM_SZE	0.684	0.552	0.756	0.533	0.51	0.549	0.571	0.566	0.55	0.55
GLZLM_LZE	9.88	7880	4.94	37900	11400	2960	11400	1150	8380	7710
GLZLM_LGZE	0.0015	0.00139	0.00329	0.00412	0.00201	0.00149	0.0013	0.00217	0.00105	0.00134
GLZLM_HGZE	1010	1520	1510	418	749	1120	1160	964	1380	1150
GLZLM_SZLGE	0.000953	0.000771	0.0031	0.00256	0.0011	0.000769	0.000748	0.00139	0.000647	0.000877
GLZLM_SZHGE	712	820	1160	221	382	609	643	526	761	599
GLZLM_LZLGE	0.015	5.26	0.00619	87.3	14.9	2.74	10	1.41	7.12	5.68
GLZLM_LZHGE	8550	11900000	7330	16800000	8770000	3260000	13200000	971000	10000000	10500000
GLZLM_GLNUz	290	119	21.2	168	451	123	230	212	397	342
GLZLM_ZLNU	3480	902	307	851	2540	884	1600	1980	2870	2720
GLZLM_ZP	0.502	0.109	0.605	0.0763	0.128	0.144	0.125	0.189	0.134	0.147

Patients with meningioma	61	62	63	64
minVaule	360	74	255	8
meanValue	1050	908	913	1190
stdValue	169	155	133	153
maxValue	2250	1720	1310	2250
HISTO_Skewness	0.873	-0.401	-0.885	-0.759
HISTO_Kurtosis	6.31	5.13	6.41	6.87
HISTO_Entropy_log10	1.35	1.38	1.48	1.23
HISTO_Energy	0.0563	0.052	0.0414	0.0714
SHAPE_Volume	6.21	7.48	1.56	21.3
GLCM_Homogeneity	0.366	0.366	0.299	0.421
GLCM_Energy	0.00534	0.00479	0.00322	0.0081
GLCM_Contrast	23.4	24.1	60.9	12.9
GLCM_Correlation	0.564	0.614	0.444	0.597
GLCM_Entropy_log10	2.48	2.53	2.69	2.28
GLCM_Dissimilarity	3.47	3.49	5.35	2.54
GLRLM_SRE	0.922	0.923	0.951	0.9
GLRLM_LRE	1.43	1.41	1.25	1.52
GLRLM_LGRE	0.00241	0.00118	0.00209	0.00103
GLRLM_HGRE	605	1120	1710	1210
GLRLM_SRLGE	0.00225	0.0011	0.00202	0.000947
GLRLM_SRHGE	561	1040	1620	1090
GLRLM_LRLGE	0.00334	0.0016	0.0025	0.00149
GLRLM_LRHGE	844	1580	2120	1820
GLRLM_GLNUr	1030	1150	202	1360
GLRLM_RLNU	15500	18700	4430	15000
GLRLM_RP	0.896	0.899	0.935	0.867
NGLDM_Coarseness	0.000414	0.000364	0.00155	0.000437
NGLDM_Contrast	0.0389	0.0454	0.114	0.0209
NGLDM_Busyness	1.06	0.892	0.184	0.755
GLZLM_SZE	0.608	0.596	0.664	0.69
GLZLM_LZE	408	345	19.4	3980
GLZLM_LGZE	0.00284	0.00157	0.00252	0.00172
GLZLM_HGZE	687	1130	1700	1170
GLZLM_SZLGE	0.0018	0.00111	0.00171	0.00144
GLZLM_SZHGE	438	667	1120	783
GLZLM_LZLGE	0.837	0.321	0.0217	3.42
GLZLM_LZHGE	209000	390000	31700	4700000
GLZLM_GLNUz	210	236	72.4	168
GLZLM_ZLNU	1840	2050	922	1700
GLZLM_ZP	0.254	0.246	0.424	0.171

Patients with Rathke cleft cyst	1	2	3	4	5	6	7	8	9	10
minVaule	93	60	75	307	63	259	58	65	67	92
meanValue	388	289	533	690	173	477	427	531	823	196
stdValue	88.4	69.6	67	108	36.8	155	165	120	108	31.7
maxValue	881	1050	1050	1340	415	1060	1350	1690	2020	412
HISTO_Skewness	0.561	2.9	0.411	0.468	2.04	1.76	0.762	0.783	-0.866	1.46
HISTO_Kurtosis	4.3	19.3	11.8	6.08	9.21	5.37	3.91	5.31	8.89	9
HISTO_Entropy_log10	1.46	1.14	1.11	1.41	1.29	1.45	1.47	1.24	1.11	1.36
HISTO_Energy	0.0429	0.104	0.126	0.0466	0.0765	0.0604	0.0394	0.0762	0.101	0.056
SHAPE_Volume	6.88	4.6	7.3	3.71	1.22	0.417	0.965	1.87	22.1	0.989
GLCM_Homogeneity	0.371	0.5	0.547	0.248	0.424	0.395	0.286	0.524	0.568	0.353
GLCM_Energy	0.004	0.0205	0.0358	0.00393	0.0124	0.0125	0.00294	0.0172	0.0227	0.00537
GLCM_Contrast	30.1	14.2	13.3	62	33.2	64	52.1	6.81	4.54	39.7
GLCM_Correlation	0.684	0.481	0.519	0.0929	0.395	0.654	0.554	0.819	0.752	0.411
GLCM_Entropy_log10	2.66	2.02	1.92	2.51	2.25	2.31	2.66	2.09	1.9	2.49
GLCM_Dissimilarity	3.77	2.2	2.03	6.07	3.49	4.64	5.3	1.75	1.39	4.11
GLRLM_SRE	0.917	0.853	0.821	0.976	0.888	0.921	0.956	0.845	0.804	0.93
GLRLM_LRE	1.55	2.26	3.28	1.1	1.87	1.6	1.2	2.58	2.58	1.42
GLRLM_LGRE	0.00243	0.00616	0.0013	0.00364	0.00402	0.00846	0.00762	0.00386	0.00195	0.00339
GLRLM_HGRE	661	265	963	637	489	512	423	382	649	501
GLRLM_SRLGE	0.00223	0.00534	0.0011	0.00359	0.00359	0.00777	0.0074	0.00336	0.00162	0.0032
GLRLM_SRHGE	611	233	794	623	447	493	406	324	517	469
GLRLM_LRLGE	0.00371	0.0128	0.00378	0.00382	0.00725	0.0139	0.00858	0.00899	0.00449	0.00456
GLRLM_LRHGE	981	539	3100	700	812	637	500	944	1710	696
GLRLM_GLNUr	1580	2170	3440	43	449	125	40.3	644	7910	303
GLRLM_RLNU	30900	16400	22200	880	5000	1960	928	6600	52500	4700
GLRLM_RP	0.883	0.798	0.735	0.968	0.845	0.883	0.941	0.784	0.738	0.903
NGLDM_Coarseness	0.000254	0.000306	0.000187	0.00685	0.000951	0.00353	0.00779	0.00116	0.000125	0.00129
NGLDM_Contrast	0.0703	0.0188	0.0152	0.182	0.061	0.226	0.206	0.0262	0.00662	0.0628
NGLDM_Busyness	2.02	2.22	1.59	0.126	0.552	0.277	0.149	0.709	3.16	0.439
GLZLM_SZE	0.598	0.612	0.631	0.795	0.645	0.699	0.717	0.579	0.556	0.607
GLZLM_LZE	690	16300	34400	3.39	1370	257	13.5	6590	129000	132
GLZLM_LGZE	0.00251	0.00774	0.00212	0.00437	0.00386	0.00676	0.0105	0.00707	0.00361	0.00426
GLZLM_HGZE	759	453	992	646	766	826	451	437	594	573
GLZLM_SZLGE	0.00141	0.00393	0.00161	0.00399	0.00179	0.00359	0.00922	0.00536	0.00218	0.00253
GLZLM_SZHGE	469	319	616	516	566	615	334	262	334	381
GLZLM_LZLGE	1.43	81.7	37.2	0.00877	4.13	2.12	0.0794	20.2	192	0.33
GLZLM_LZHGE	366000	3350000	31900000	2080	469000	34500	4670	2240000	88500000	59400
GLZLM_GLNUz	331	139	184	26.5	45.4	20.7	18.2	45.7	307	68.2
GLZLM_ZLNU	3330	1190	2090	389	565	398	256	339	2200	607
GLZLM_ZP	0.233	0.119	0.124	0.684	0.193	0.33	0.486	0.0914	0.0658	0.287

Patients with Rathke cleft cyst	11	12	13	14	15	16	17	18	19	20
minVaule	71	143	199	100	159	59	111	118	221	166
meanValue	253	619	504	250	373	221	430	234	477	973
stdValue	52.2	112	84.1	46.8	62.7	42.9	108	37.7	86.6	131
maxValue	781	859	882	571	819	591	887	572	887	1770
HISTO_Skewness	2.36	-0.808	1.01	2.02	2.49	1.56	0.43	2.8	1.47	-1.28
HISTO_Kurtosis	14.1	3.31	5.96	10.5	11.4	8.56	2.97	15.5	6.16	7.72
HISTO_Entropy_log10	1.17	1.57	1.43	1.28	1.22	1.25	1.53	1.19	1.42	1.23
HISTO_Energy	0.0972	0.0325	0.0528	0.0805	0.0906	0.0784	0.0338	0.092	0.0568	0.0833
SHAPE_Volume	1.34	5.26	2.11	1.48	3.84	2.98	4.06	2.07	1.71	23.1
GLCM_Homogeneity	0.444	0.277	0.301	0.442	0.434	0.424	0.364	0.425	0.358	0.529
GLCM_Energy	0.0184	0.00241	0.00546	0.014	0.0152	0.0121	0.00326	0.015	0.00824	0.0188
GLCM_Contrast	23.6	76.8	67.5	30.6	26.7	25.4	37.6	28.5	66.1	17.4
GLCM_Correlation	0.415	0.492	0.168	0.439	0.426	0.369	0.753	0.311	0.378	0.549
GLCM_Entropy_log10	2.1	2.85	2.47	2.24	2.17	2.23	2.73	2.14	2.49	2.09
GLCM_Dissimilarity	2.94	6.35	5.54	3.21	3.08	3.15	4.13	3.14	5.11	2.29
GLRLM_SRE	0.89	0.953	0.953	0.889	0.892	0.898	0.918	0.902	0.924	0.839
GLRLM_LRE	1.92	1.28	1.25	1.83	1.79	1.75	1.58	1.66	1.47	2.28
GLRLM_LGRE	0.00441	0.00118	0.00312	0.00386	0.0027	0.00339	0.00213	0.00447	0.00262	0.00135
GLRLM_HGRE	320	1930	918	493	508	439	818	325	721	1070
GLRLM_SRLGE	0.00391	0.00114	0.00305	0.00351	0.00239	0.00306	0.00194	0.00402	0.00245	0.0012
GLRLM_SRHGE	292	1830	881	448	466	400	761	301	680	888
GLRLM_LRLGE	0.00852	0.00142	0.00347	0.00637	0.0049	0.00573	0.00351	0.00748	0.00364	0.00249
GLRLM_LRHGE	548	2560	1110	826	811	720	1190	488	970	2550
GLRLM_GLNUr	492	254	77.3	566	1340	644	742	769	146	525
GLRLM_RLNU	4380	7140	1360	5980	12600	6980	18500	7140	2360	4820
GLRLM_RP	0.843	0.933	0.935	0.844	0.848	0.858	0.884	0.865	0.894	0.773
NGLDM_Coarseness	0.00106	0.000997	0.00437	0.000887	0.000372	0.00077	0.000428	0.000713	0.0029	0.0011
NGLDM_Contrast	0.0298	0.211	0.132	0.0543	0.0438	0.0411	0.121	0.0365	0.13	0.0289
NGLDM_Busyness	0.632	0.303	0.103	0.608	1.3	0.799	1.18	0.946	0.188	0.297
GLZLM_SZE	0.58	0.691	0.713	0.647	0.672	0.647	0.612	0.658	0.714	0.704
GLZLM_LZE	1900	25.4	21.9	1870	5320	1920	615	1980	242	3270
GLZLM_LGZE	0.0049	0.00133	0.00499	0.00461	0.00247	0.00359	0.00211	0.00411	0.00326	0.0026
GLZLM_HGZE	466	1700	1050	721	807	600	1010	514	968	900
GLZLM_SZLGE	0.00333	0.000856	0.0046	0.00214	0.00162	0.00196	0.00129	0.00228	0.0026	0.00216
GLZLM_SZHGE	305	1090	793	528	604	428	649	395	747	618
GLZLM_LZLGE	8.6	0.0155	0.0341	4.87	14.9	5.57	1.99	8.71	0.502	2.83
GLZLM_LZHGE	424000	58800	16700	731000	1920000	675000	268000	456000	120000	3820000
GLZLM_GLNUz	59.2	102	23.7	56.2	133	78.6	177	95.9	30	58.5
GLZLM_ZLNU	381	1630	340	703	1570	798	2130	905	499	745
GLZLM_ZP	0.182	0.433	0.445	0.198	0.197	0.2	0.242	0.217	0.334	0.174

Patients with Rathke cleft cyst	21	22	23	24	25	26	27	28	29	30
minVaule	272	132	141	15	49	40	374	157	9	170
meanValue	407	460	498	435	328	611	1100	406	227	350
stdValue	77.3	101	92.1	75.1	61	169	137	93.4	149	65.1
maxValue	796	1100	1410	1140	856	1570	1460	942	1420	727
HISTO_Skewness	2.08	1.31	2.79	1.61	3.23	1.23	-0.709	1.91	2.18	1.68
HISTO_Kurtosis	7.41	9.16	21.3	15.3	17.4	5.29	4.05	7.23	10	7.17
HISTO_Entropy_log10	1.39	1.33	1.18	1.17	1.07	1.39	1.5	1.34	1.29	1.4
HISTO_Energy	0.062	0.0697	0.0843	0.095	0.129	0.0533	0.0397	0.0701	0.0703	0.0544
SHAPE_Volume	0.662	4.29	1.49	1.36	3.05	1.82	22.2	0.844	11	1.02
GLCM_Homogeneity	0.363	0.394	0.411	0.502	0.525	0.417	0.382	0.416	0.355	0.344
GLCM_Energy	0.0078	0.0113	0.0111	0.0197	0.0309	0.0082	0.00558	0.011	0.00895	0.00573
GLCM_Contrast	98.5	43.3	15.7	13.4	17.8	33.6	54.5	50.6	40.5	56.3
GLCM_Correlation	0.236	0.451	0.356	0.565	0.423	0.645	0.474	0.329	0.368	0.319
GLCM_Entropy_log10	2.45	2.33	2.14	2.05	1.87	2.48	2.62	2.31	2.32	2.52
GLCM_Dissimilarity	5.57	3.94	2.72	2.23	2.19	3.69	4.68	3.97	4.26	4.75
GLRLM_SRE	0.922	0.906	0.916	0.85	0.835	0.897	0.893	0.9	0.933	0.93
GLRLM_LRE	1.48	1.78	1.46	2.41	2.59	1.88	2.38	1.7	1.35	1.43
GLRLM_LGRE	0.00779	0.00452	0.00372	0.00215	0.00222	0.00254	0.000798	0.00356	0.0235	0.00325
GLRLM_HGRE	403	655	367	619	559	669	1940	515	162	518
GLRLM_SRLGE	0.00715	0.00411	0.00344	0.00188	0.00186	0.00226	0.000732	0.00322	0.0216	0.00303
GLRLM_SRHGE	387	598	338	530	480	616	1720	479	157	491
GLRLM_LRLGE	0.0117	0.00694	0.00524	0.00464	0.00573	0.0048	0.00156	0.00588	0.0333	0.00462
GLRLM_LRHGE	501	1120	519	1460	1300	1100	4730	766	186	684
GLRLM_GLNUr	213	851	377	576	1650	371	489	288	148	303
GLRLM_RLNU	3060	8660	3770	4760	9980	6000	10700	3550	1890	4920
GLRLM_RP	0.89	0.861	0.888	0.791	0.77	0.845	0.842	0.858	0.91	0.902
NGLDM_Coarseness	0.00144	0.00169	0.00155	0.00116	0.000378	0.000986	0.000595	0.00126	0.00268	0.00117
NGLDM_Contrast	0.218	0.104	0.025	0.0162	0.0239	0.0757	0.143	0.109	0.132	0.112
NGLDM_Busyness	0.588	0.838	0.414	0.363	1.38	0.586	0.633	0.512	0.74	0.516
GLZLM_SZE	0.741	0.662	0.608	0.616	0.635	0.657	0.652	0.655	0.696	0.661
GLZLM_LZE	307	6720	425	4160	16300	792	1460	794	128	179
GLZLM_LGZE	0.00595	0.00476	0.00473	0.00386	0.0024	0.00246	0.00121	0.00372	0.0198	0.00337
GLZLM_HGZE	702	778	454	722	895	830	1670	838	276	732
GLZLM_SZLGE	0.00352	0.00312	0.00325	0.00306	0.00165	0.00142	0.000692	0.00196	0.0133	0.0023
GLZLM_SZHGE	588	543	310	475	633	557	1030	629	225	550
GLZLM_LZLGE	1.89	14.3	1.38	7.24	35.9	2.07	0.74	2.42	3.64	0.566
GLZLM_LZHGE	54900	4070000	135000	2420000	7440000	315000	2960000	267000	5520	61700
GLZLM_GLNUz	35.6	85.7	61.4	42.1	82	76.8	76.9	31.9	36.7	60.8
GLZLM_ZLNU	671	955	407	371	825	889	1140	475	394	775
GLZLM_ZP	0.316	0.268	0.227	0.124	0.116	0.245	0.189	0.228	0.359	0.298

Patients with Rathke cleft cyst	31	32	33	34	35	36	37	38	39	40
minVaule	282	24	168	103	184	62	356	85	69	7
meanValue	470	388	240	288	368	314	1610	644	350	178
stdValue	122	106	28.5	49.5	63.6	108	789	89.2	81.4	39.3
maxValue	1200	1110	388	536	768	785	4450	922	968	633
HISTO_Skewness	2.52	1.4	1.11	1.23	1.04	1.11	0.879	-1.72	2.07	2.71
HISTO_Kurtosis	10.9	6.88	5.82	7.3	5.18	3.43	2.91	8.29	14.2	17.3
HISTO_Entropy_log10	1.35	1.36	1.48	1.42	1.42	1.41	1.59	1.32	1.28	1.08
HISTO_Energy	0.0662	0.0545	0.0439	0.0498	0.0469	0.062	0.0346	0.0726	0.0713	0.122
SHAPE_Volume	0.987	4.13	0.204	0.645	1.36	6.42	1.86	2.68	2.43	5.11
GLCM_Homogeneity	0.373	0.444	0.269	0.319	0.387	0.381	0.262	0.385	0.332	0.498
GLCM_Energy	0.00934	0.00854	0.00375	0.00459	0.0053	0.0126	0.00332	0.0111	0.00701	0.0259
GLCM_Contrast	68	17.5	65.3	50.1	38.4	79.6	155	60.5	37.6	11.6
GLCM_Correlation	0.287	0.714	0.286	0.289	0.47	0.525	0.45	0.114	0.205	0.41
GLCM_Entropy_log10	2.33	2.35	2.57	2.55	2.52	2.52	2.85	2.32	2.36	1.91
GLCM_Dissimilarity	4.71	2.75	5.9	4.93	3.87	5.64	8.71	4.56	4.11	2.08
GLRLM_SRE	0.927	0.88	0.962	0.941	0.915	0.901	0.952	0.919	0.941	0.857
GLRLM_LRE	1.48	2.19	1.19	1.34	1.57	1.91	1.26	1.54	1.28	2.25
GLRLM_LGRE	0.0147	0.00285	0.00602	0.00211	0.00395	0.00279	0.00793	0.00101	0.0036	0.00365
GLRLM_HGRE	272	550	541	833	485	663	578	1910	458	349
GLRLM_SRLGE	0.0139	0.0025	0.00581	0.00202	0.00366	0.00242	0.0074	0.000969	0.00344	0.00314
GLRLM_SRHGE	261	497	526	788	451	626	565	1750	434	305
GLRLM_LRLGE	0.0203	0.00638	0.00697	0.00262	0.00594	0.00638	0.0109	0.00129	0.00436	0.00808
GLRLM_LRHGE	338	1050	620	1090	720	965	641	2980	571	723
GLRLM_GLNUr	102	1100	51.6	180	338	1360	115	209	156	2810
GLRLM_RLNU	1380	16500	1100	3220	6110	21600	3180	2590	1950	18400
GLRLM_RP	0.895	0.828	0.947	0.919	0.881	0.849	0.933	0.887	0.921	0.802
NGLDM_Coarseness	0.00384	0.000489	0.00584	0.00178	0.00112	0.000172	0.0015	0.00214	0.00278	0.000235
NGLDM_Contrast	0.146	0.034	0.176	0.0943	0.0852	0.193	0.525	0.0892	0.0613	0.0144
NGLDM_Busyness	0.275	1.02	0.12	0.229	0.593	3.66	0.79	0.127	0.211	2.38
GLZLM_SZE	0.68	0.589	0.72	0.638	0.663	0.687	0.749	0.68	0.736	0.586
GLZLM_LZE	143	4190	8.81	54.2	369	3680	51.5	353	158	22700
GLZLM_LGZE	0.0167	0.00282	0.00627	0.00311	0.00438	0.00189	0.00557	0.00201	0.0049	0.0042
GLZLM_HGZE	485	808	651	925	651	930	773	1730	545	539
GLZLM_SZLGE	0.00969	0.00145	0.00367	0.00256	0.00262	0.00121	0.0033	0.00181	0.00399	0.00271
GLZLM_SZHGE	399	511	535	610	476	651	615	1110	435	359
GLZLM_LZLGE	1.42	13.3	0.0371	0.0794	0.994	15.6	0.623	0.177	0.388	81.2
GLZLM_LZHGE	16500	1390000	3820	40000	164000	880000	6140	712000	66100	6400000
GLZLM_GLNUz	18.7	139	21.2	47.1	65.8	281	44.1	30.6	35.1	161
GLZLM_ZLNU	246	1160	313	520	861	4100	981	428	430	1120
GLZLM_ZP	0.313	0.141	0.514	0.343	0.25	0.294	0.493	0.284	0.346	0.11
