# Supplementary figures and images for: Machine-Learning Classifiers in Discrimination of Lesions Located in the Anterior Skull Base
Source: Front Oncol. 2020 May 28;10:752. doi: 10.3389/fonc.2020.00752 (PMC7270197; doi:10.3389/fonc.2020.00752)

Craniopharyngioma

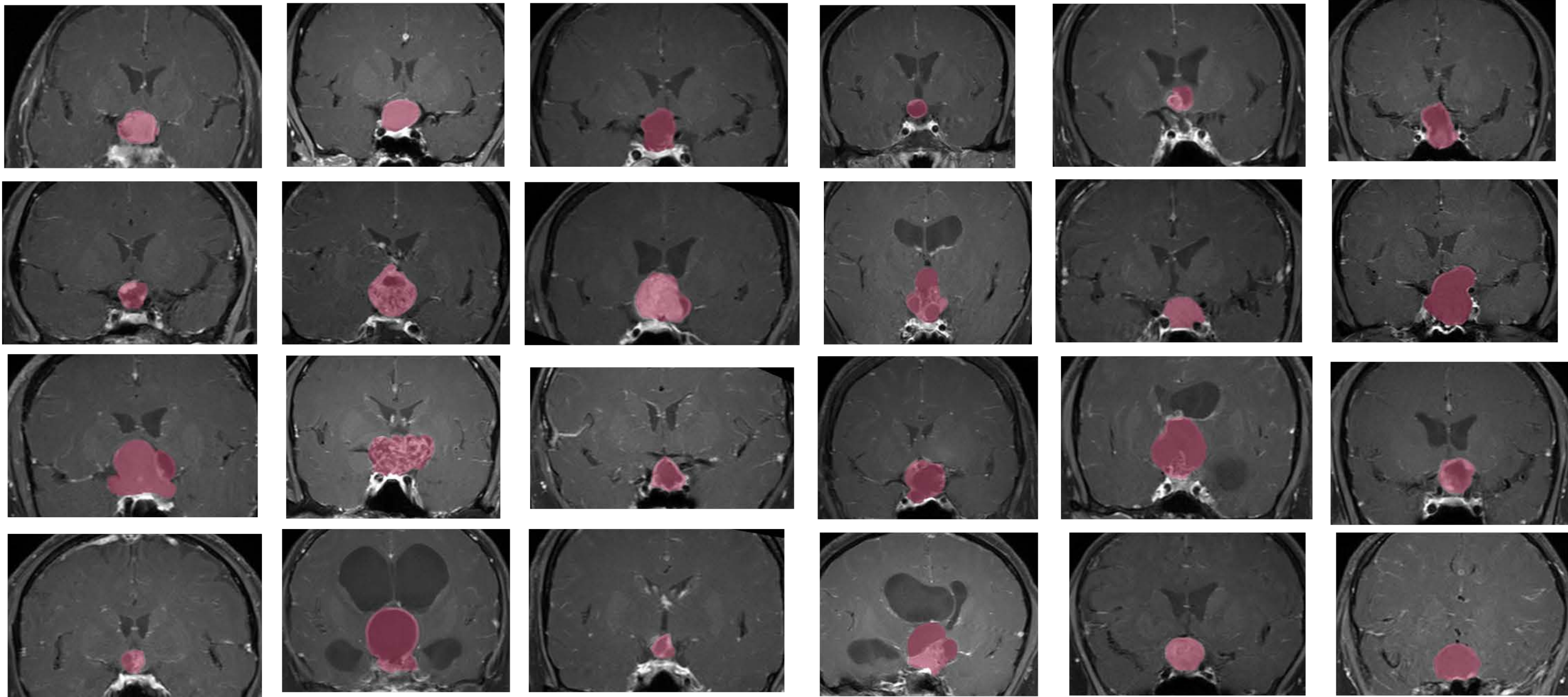

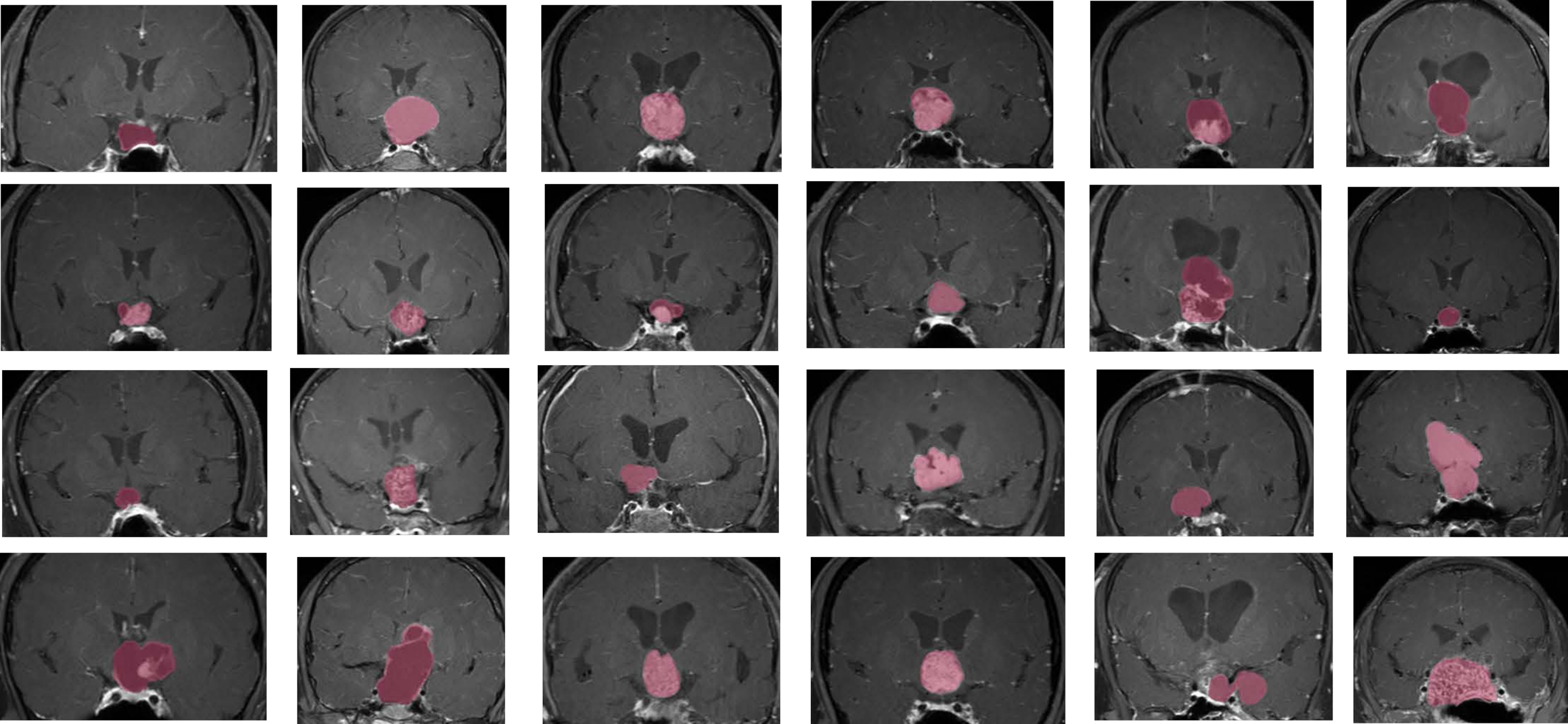

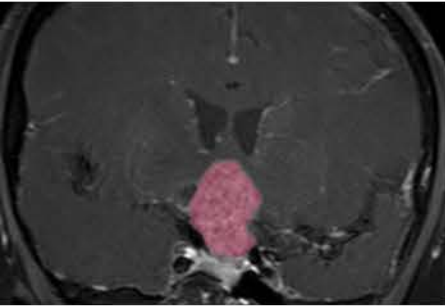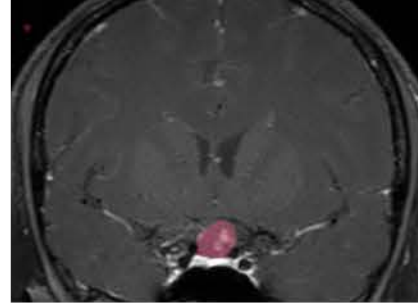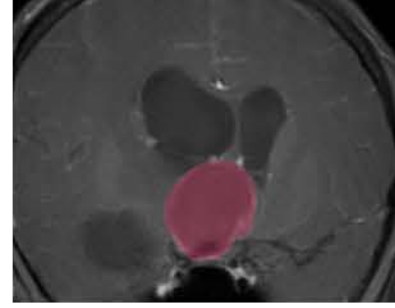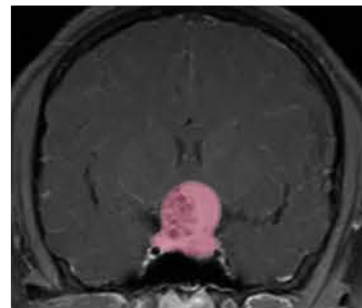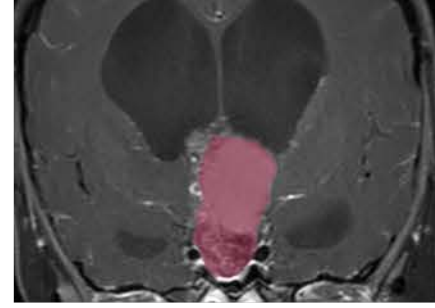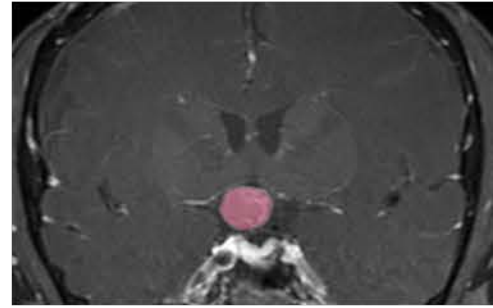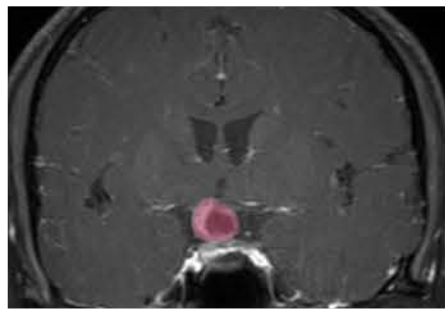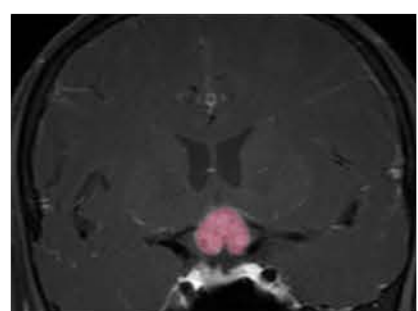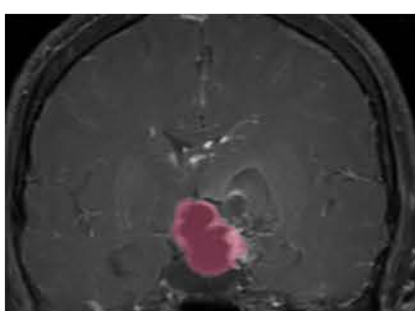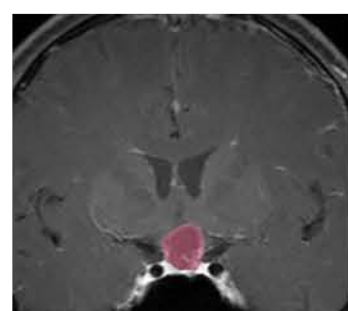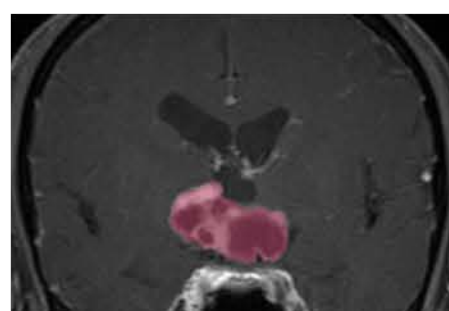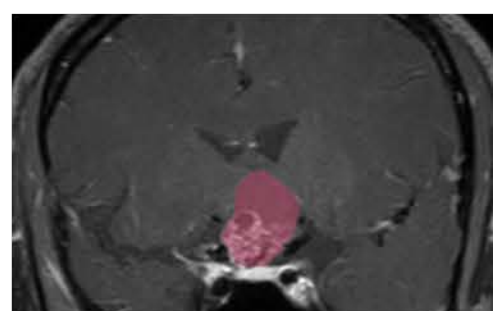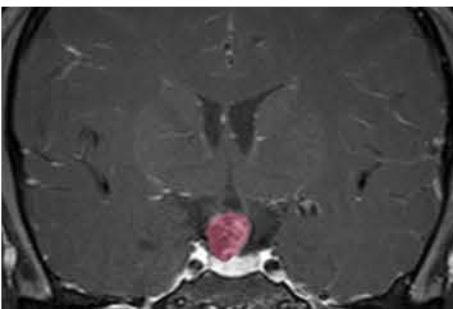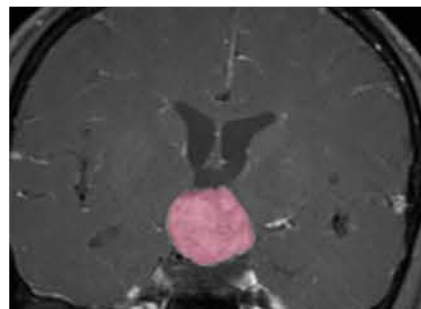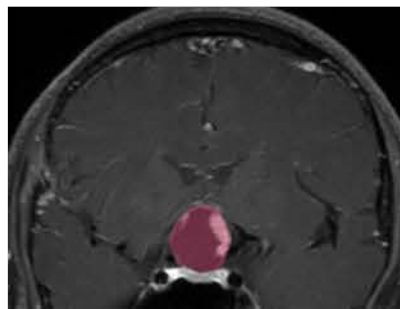

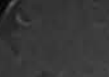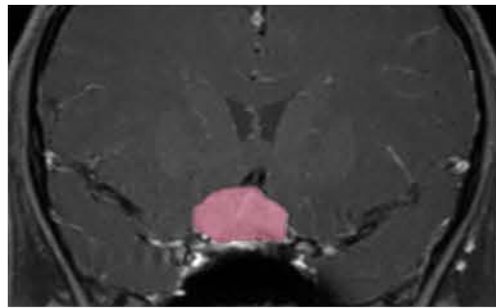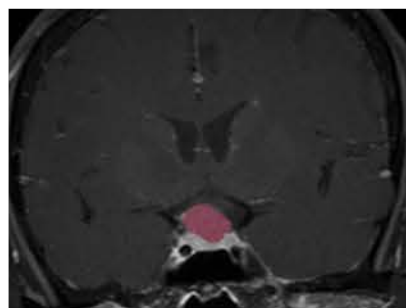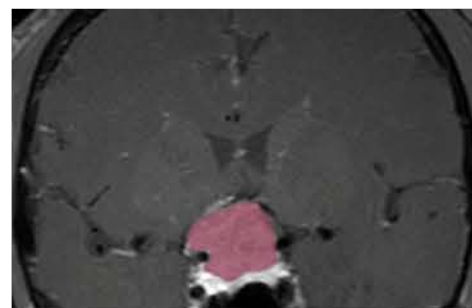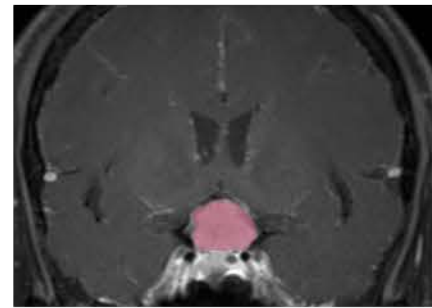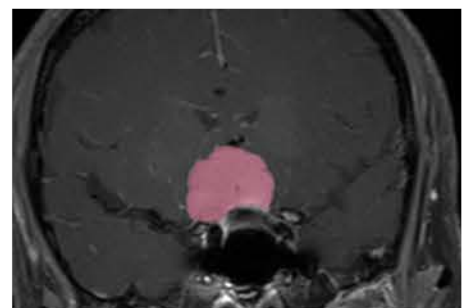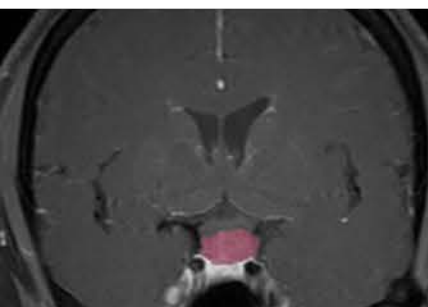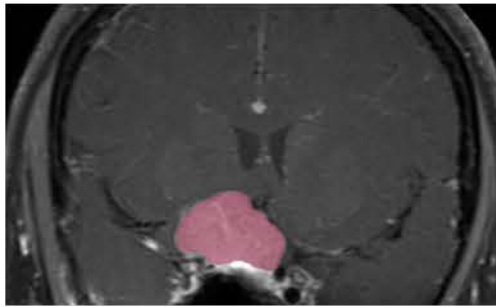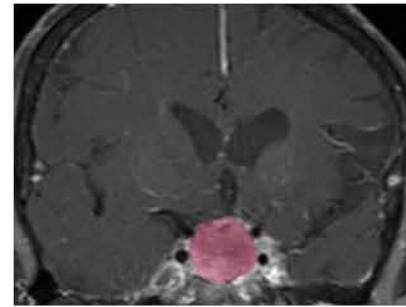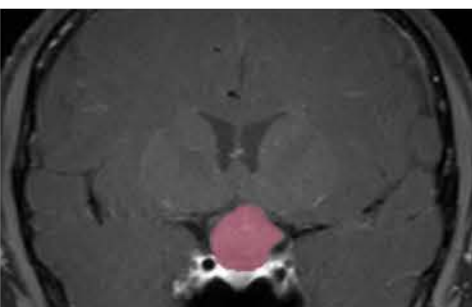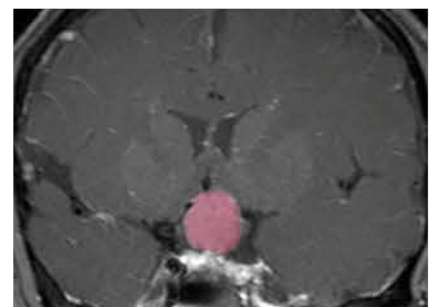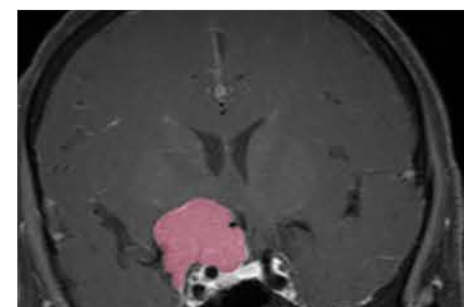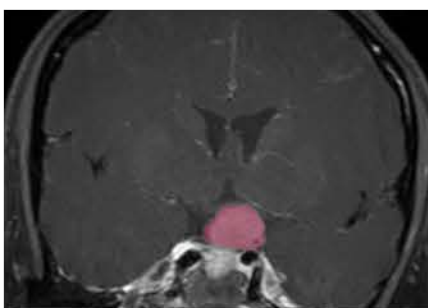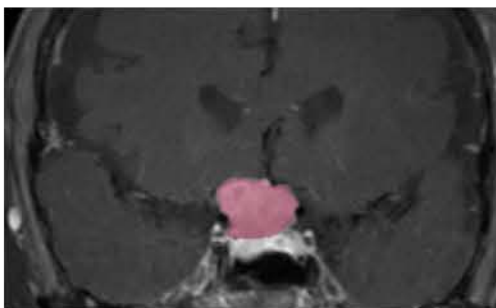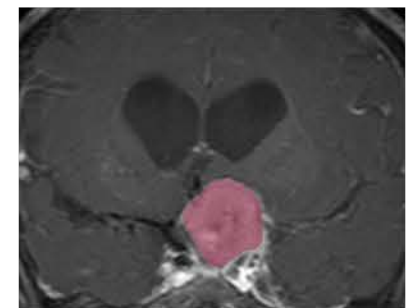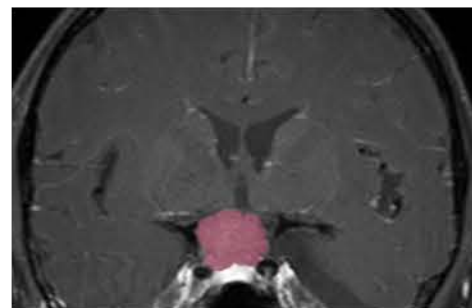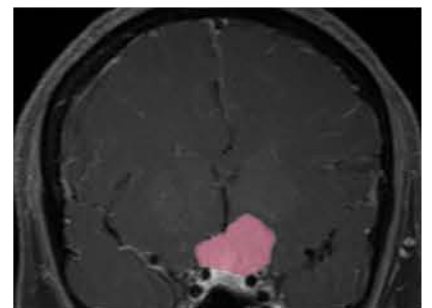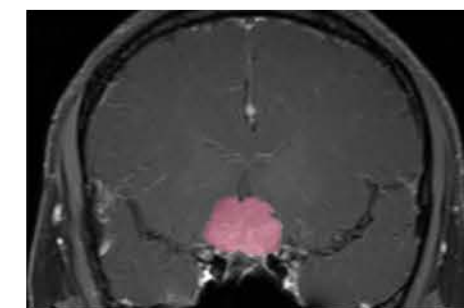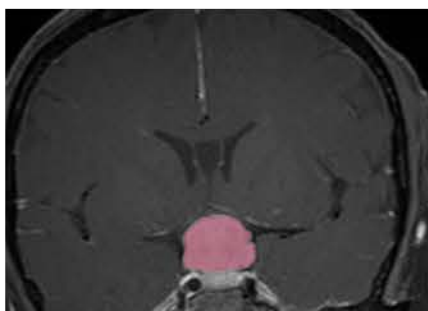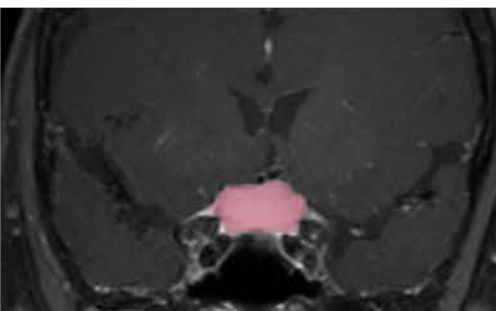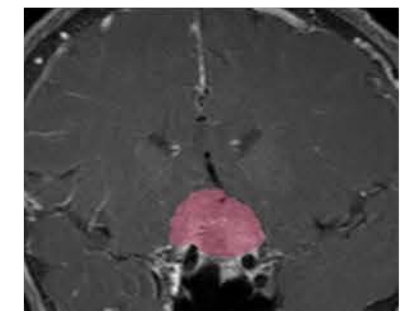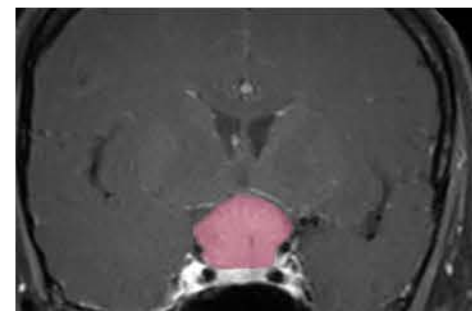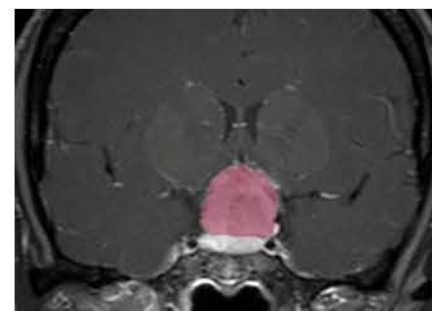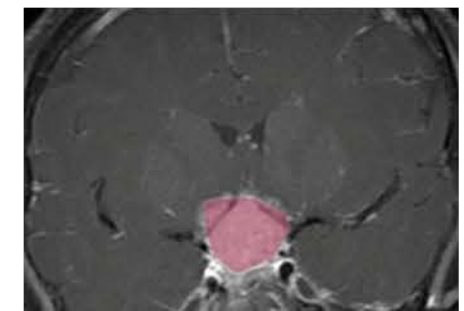

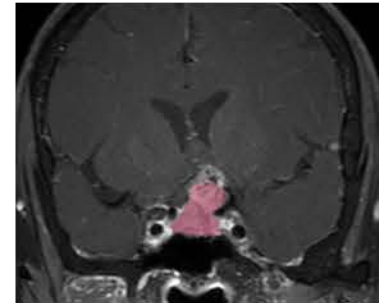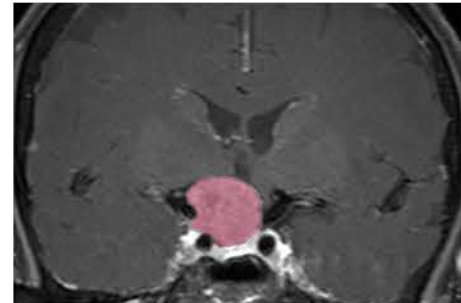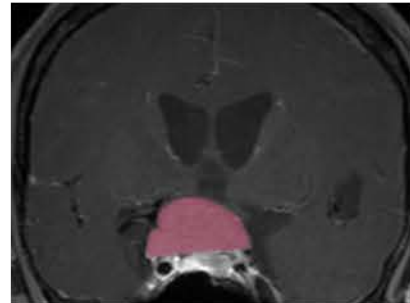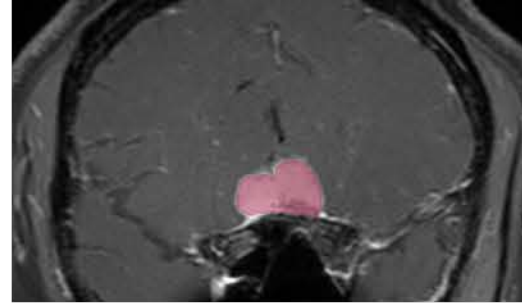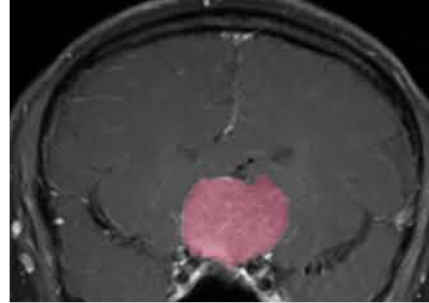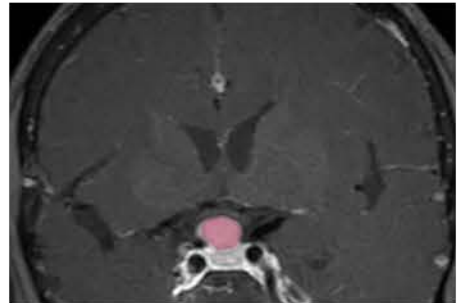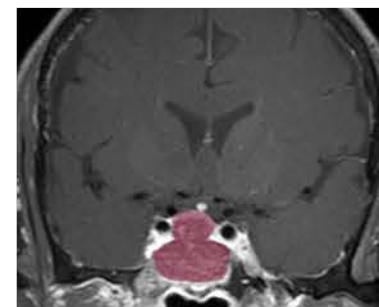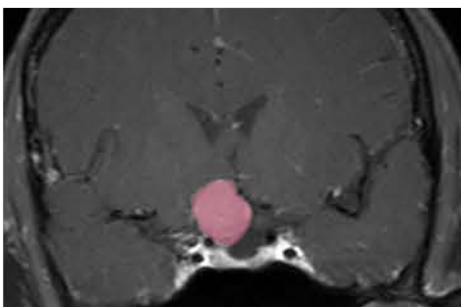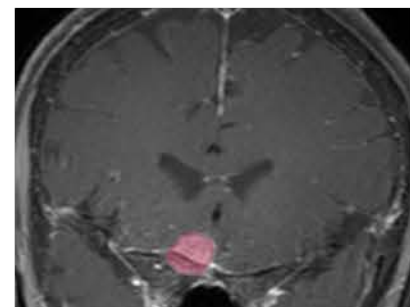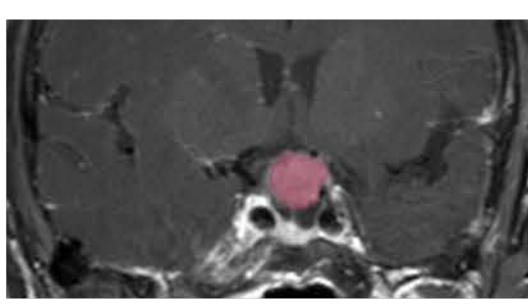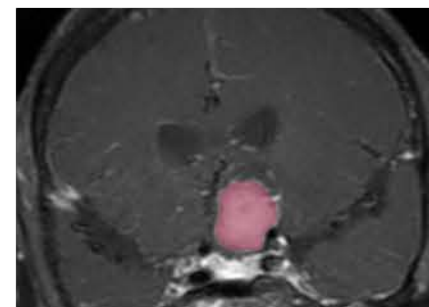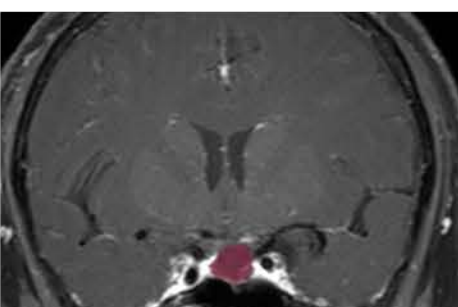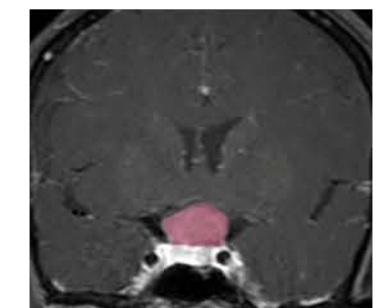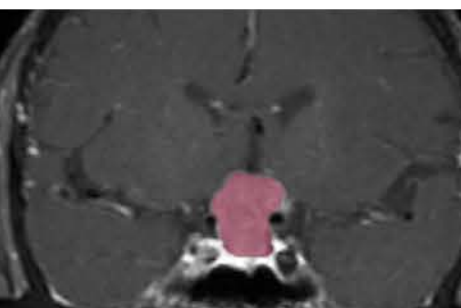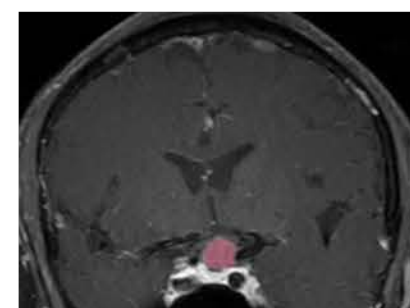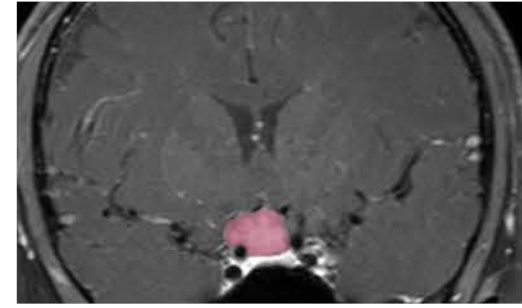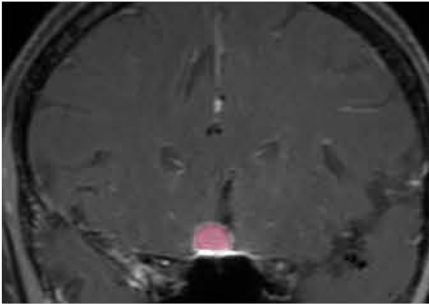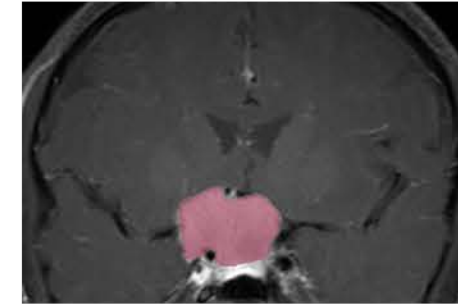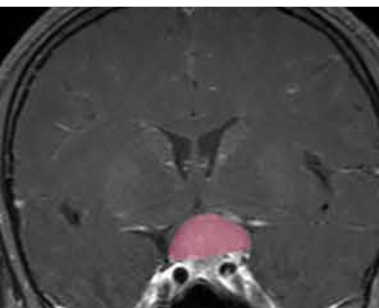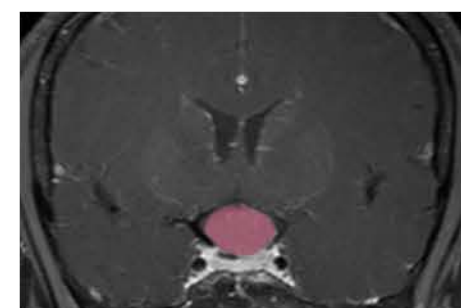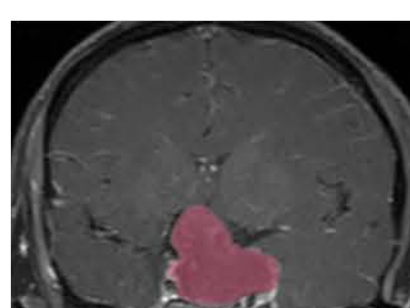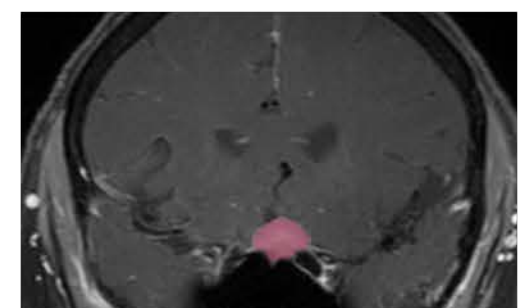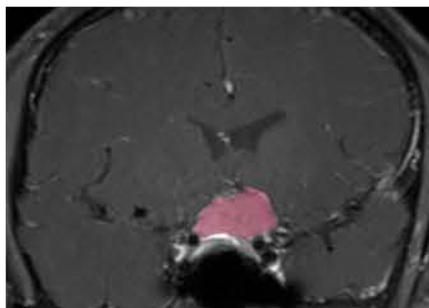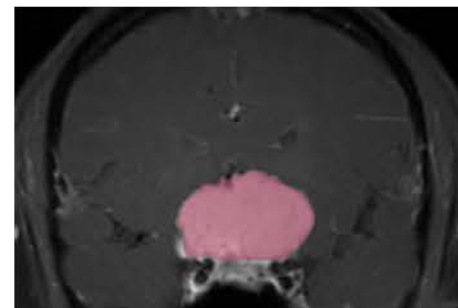

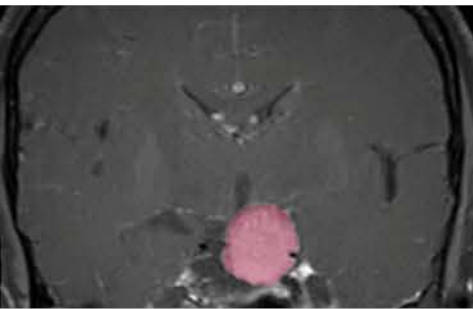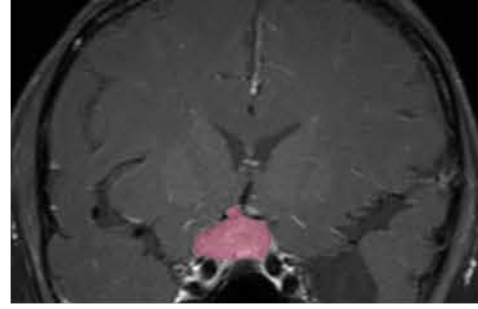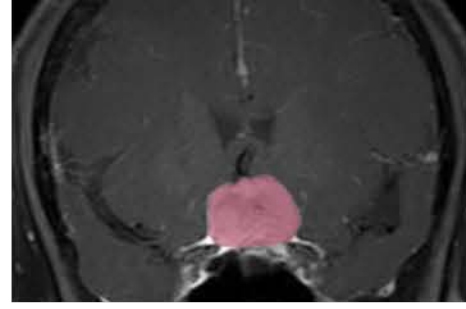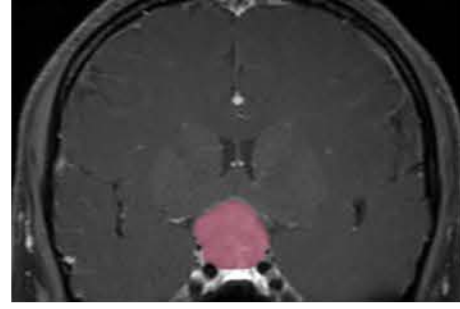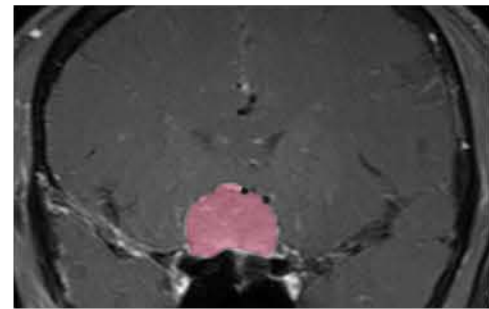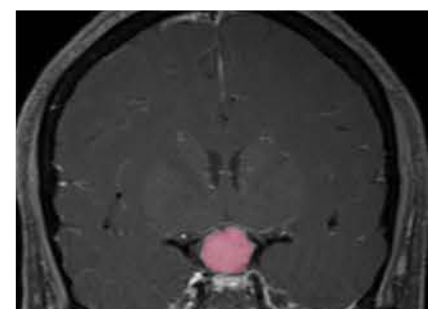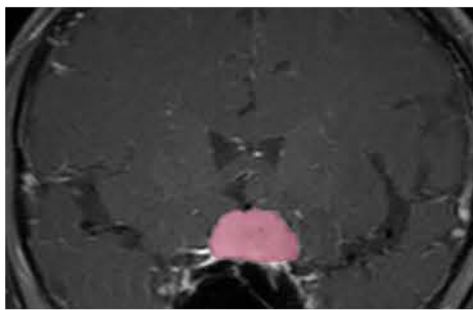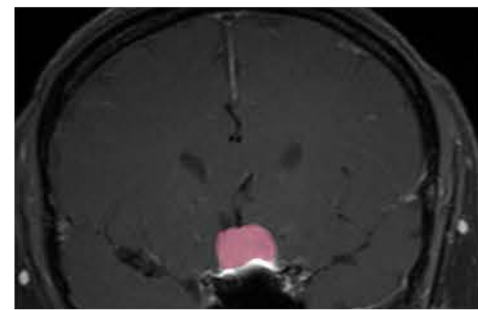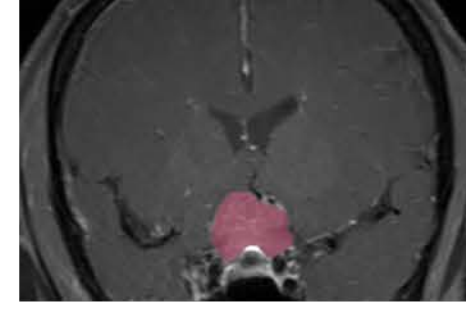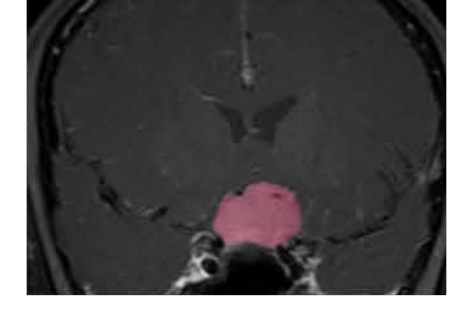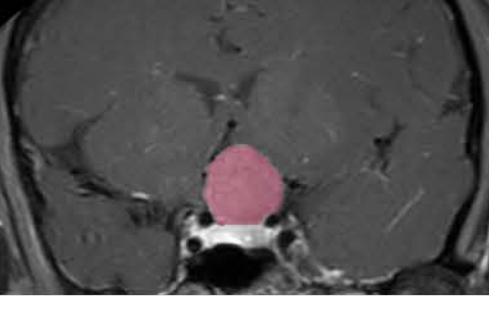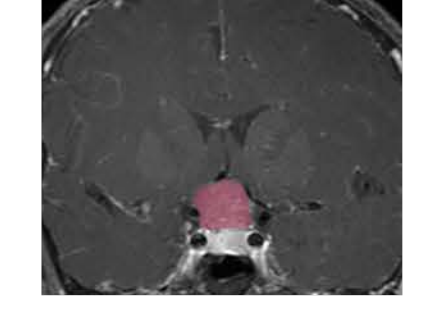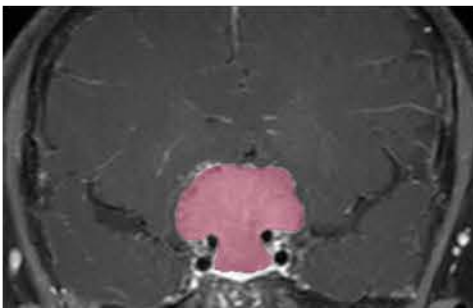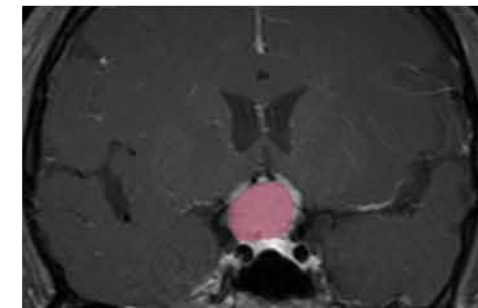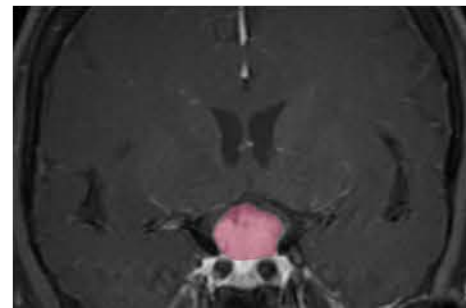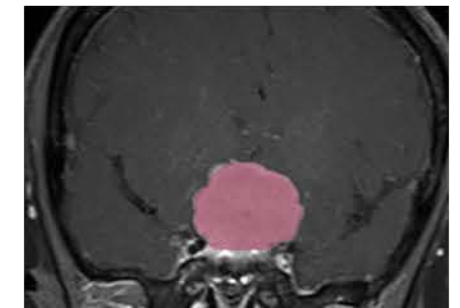

## Pituitary adenoma

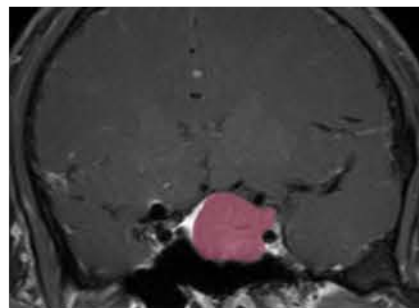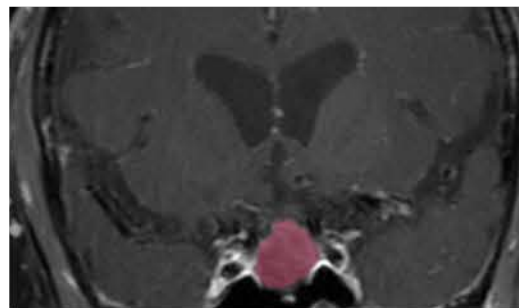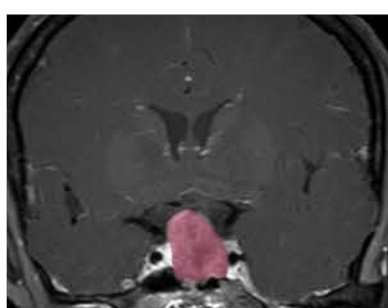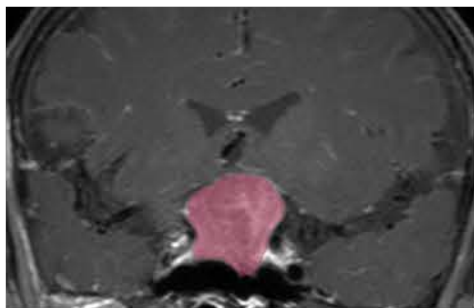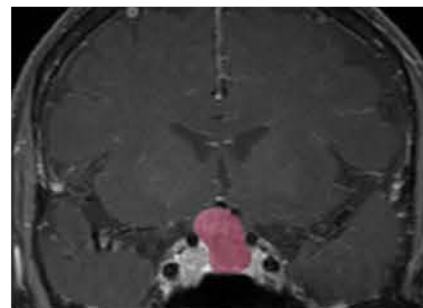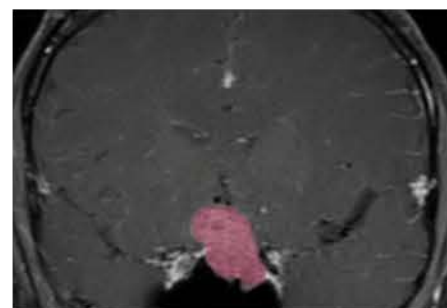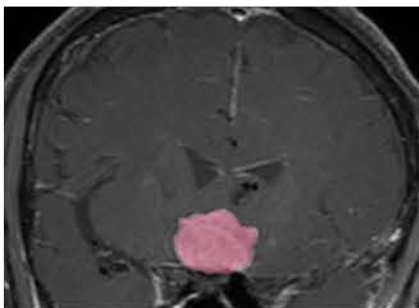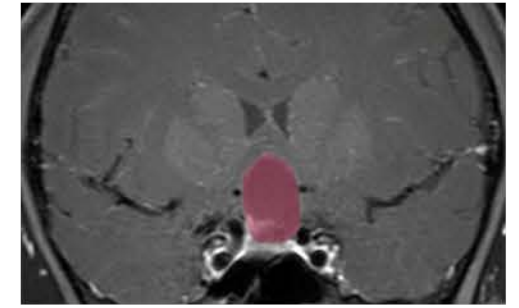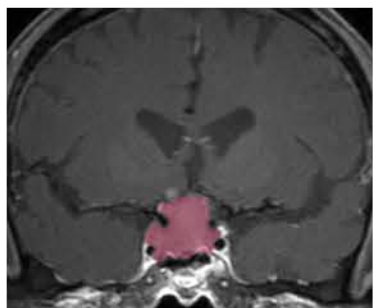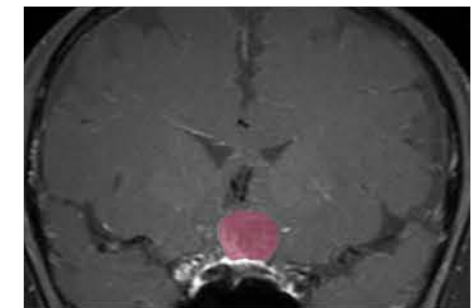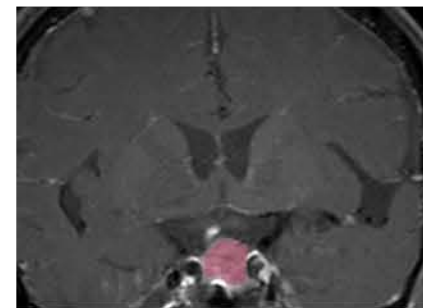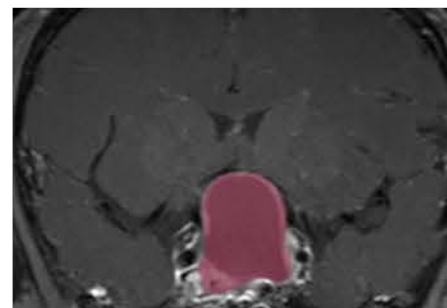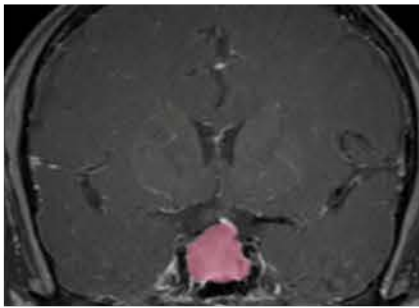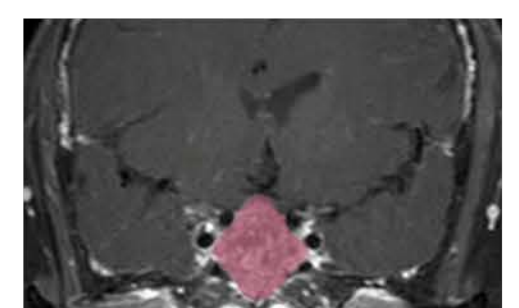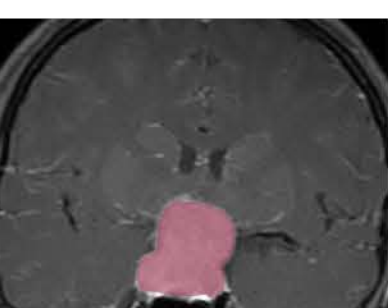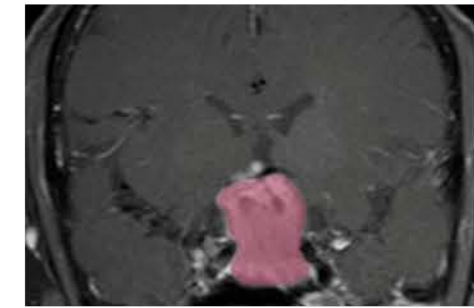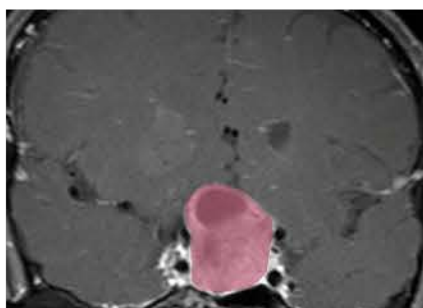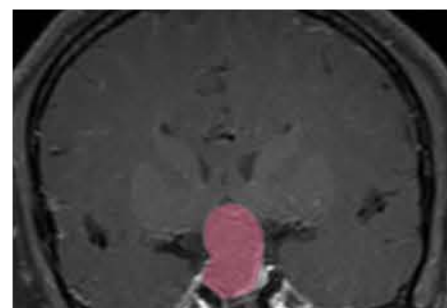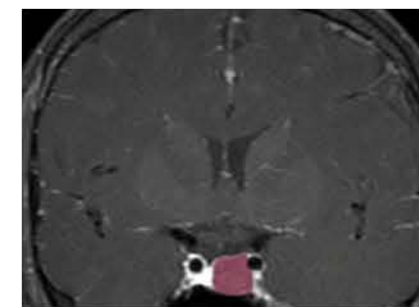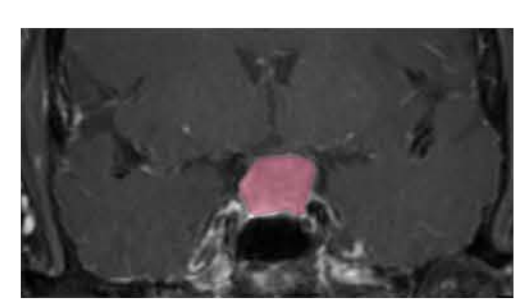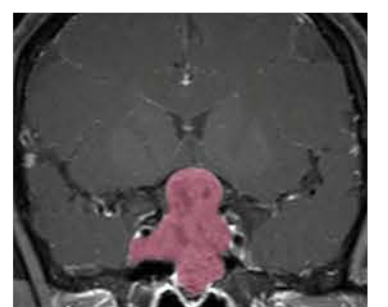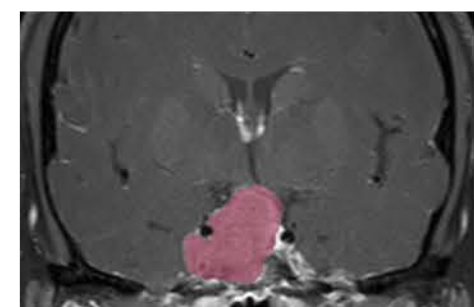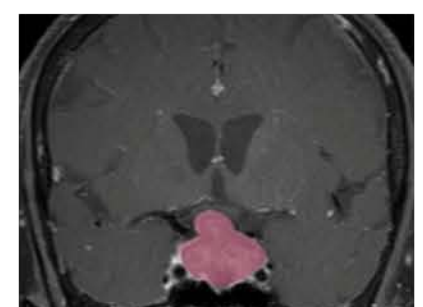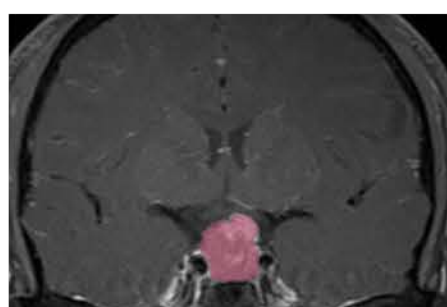

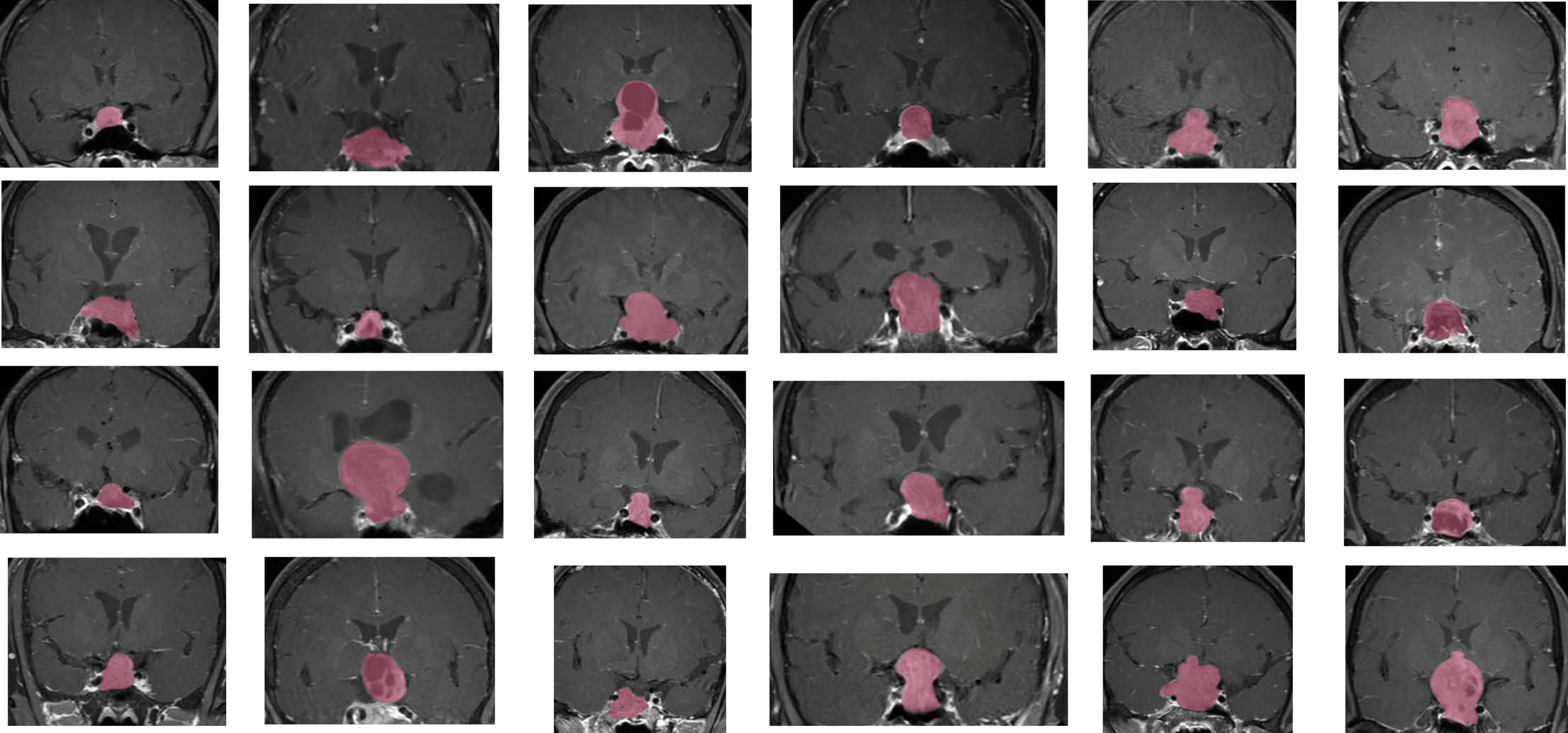

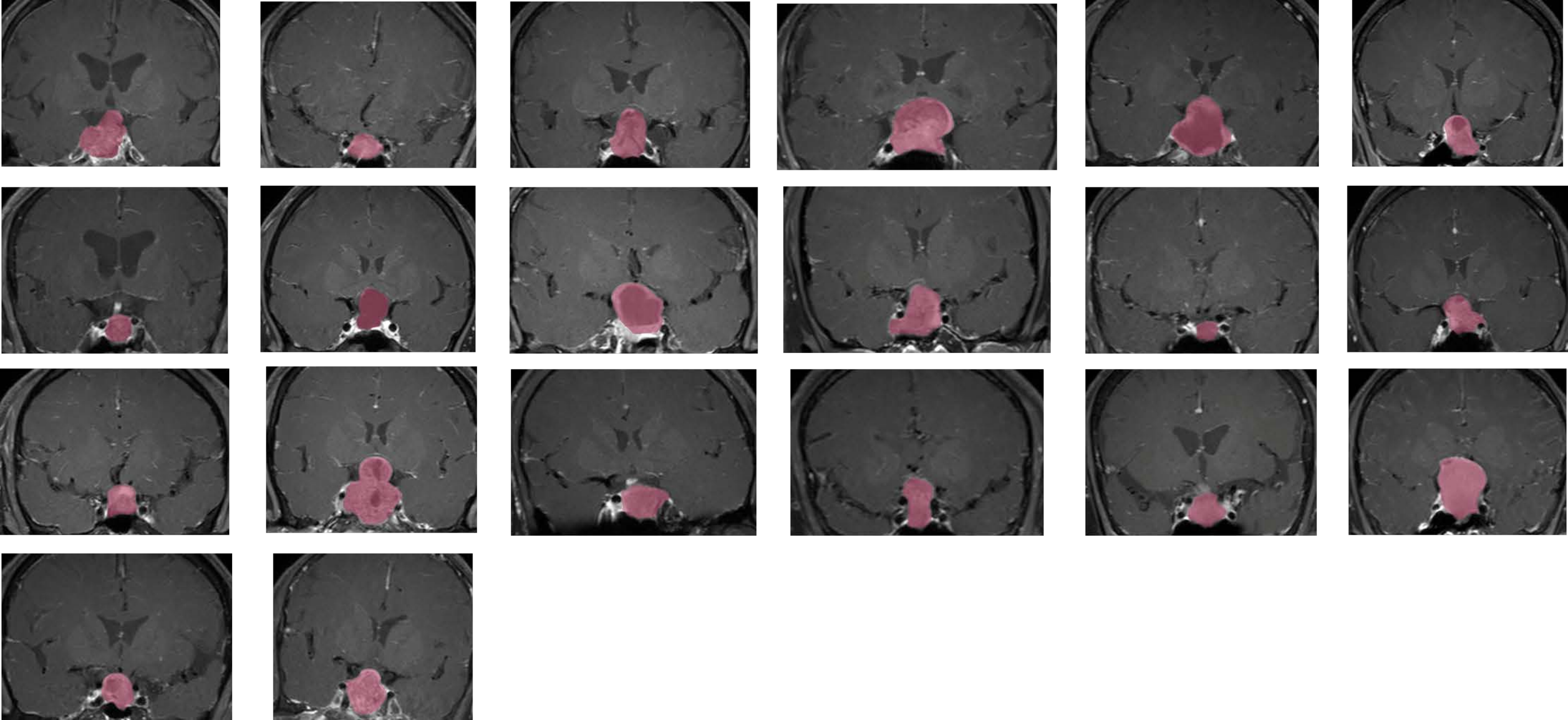

Rathke cleft cyst

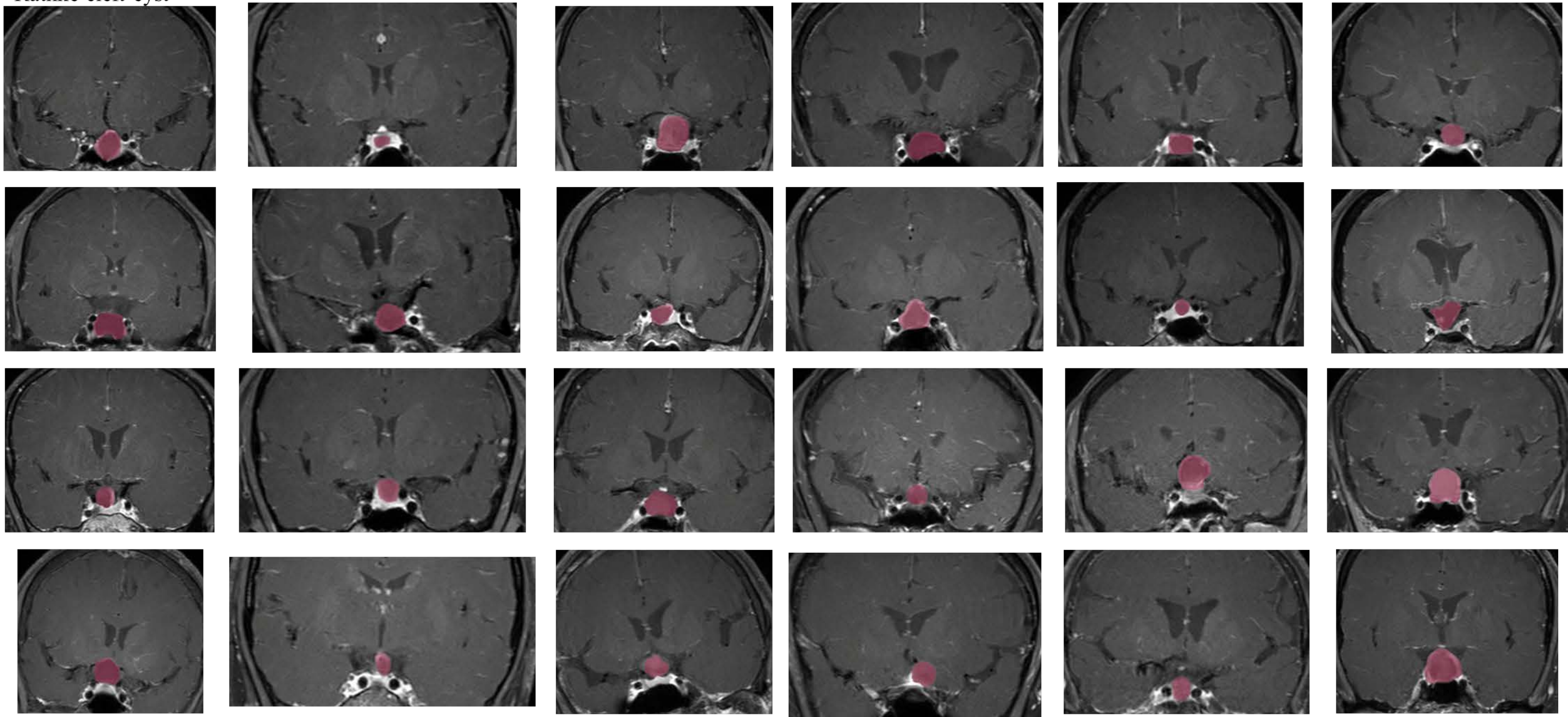

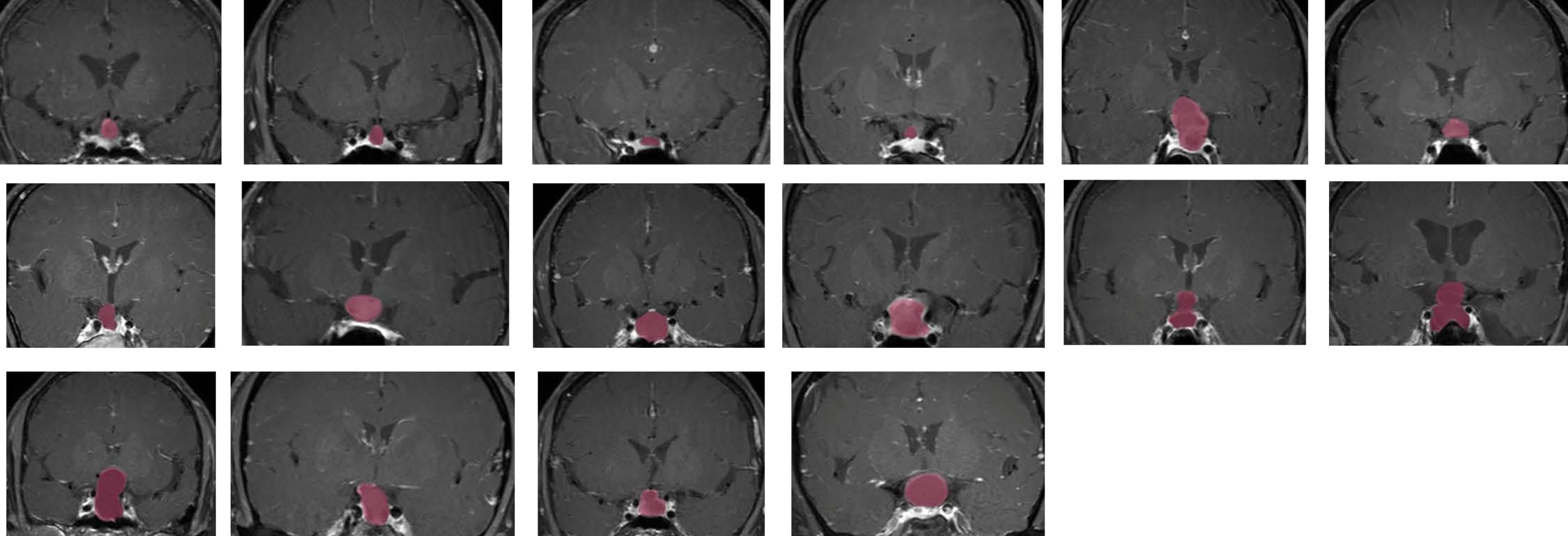

Supplement: Supplementary Material 3 — Examples of ROI delineation in MR images. [file Data_Sheet_3.PDF]
